# Physical Activity Following Hip Arthroscopy in Young and Middle-Aged Adults: A Systematic Review

**DOI:** 10.1186/s40798-020-0234-8

**Published:** 2020-01-28

**Authors:** Denise M. Jones, Kay M. Crossley, Ilana N. Ackerman, Harvi F. Hart, Karen L. Dundules, Michael J. O’Brien, Benjamin F. Mentiplay, Joshua J. Heerey, Joanne L. Kemp

**Affiliations:** 1 La Trobe Sport and Exercise Medicine Research Centre, School of Allied Health, College of Science, Health and Engineering, La Trobe University, Melbourne, Victoria Australia; 2 Monash University, Melbourne, Victoria Australia; 3 Department of Physical Therapy, The University of Western Ontario, London, Ontario Canada

**Keywords:** Outcomes, Hip-arthroscopy, Activity, Sport, Rehabilitation

## Abstract

**Background:**

Hip arthroscopy is a common surgical intervention for young and middle-aged adults with hip-related pain and dysfunction, who have high expectations for returning to physical activity following surgery. The purpose of this review was to evaluate the impact of hip arthroscopy on physical activity post-arthroscopy.

**Methods:**

A systematic search of electronic databases was undertaken in identifying studies from January 1st 1990 to December 5th 2019. The search included English language articles reporting physical activity as an outcome following hip arthroscopy in adults aged 18-50 years. Quality assessment, data extraction and synthesis of included studies were undertaken.

**Results:**

Full text articles (*n* = 234) were assessed for eligibility following screening of titles and abstracts (*n* = 2086), yielding 120 studies for inclusion. The majority (86%) of the studies were level 4 evidence. One study reported objective activity data. The most frequently occurring patient-reported outcome measure was the Hip Outcome Score-sport-specific subscale (HOS-SS, 84% of studies). Post--arthroscopy improvement was indicated by large effect sizes for patient-reported outcome measures (standard paired difference [95% confidence interval] −1.35[−1.61 to −1.09] at more than 2 years post-arthroscopy); however, the majority of outcome scores for the HOS-SS did not meet the defined level for a patient-acceptable symptom state.

**Conclusion:**

The current level of available information regarding physical activity for post arthroscopy patients is limited in scope. Outcomes have focused on patients’ perceived difficulties with sport-related activities with a paucity of information on the type, quality and quantity of activity undertaken.

**Level of Evidence:**

Level IV, systematic review of Level 2 through to Level 4 studies

## Key Points

- The systematic collection of a range of physical activity outcomes is required in both clinical and research settings to effectively monitor and support post-arthroscopy recovery, building a more comprehensive activity profile of patients that moves beyond athletic classification.
- Physical activity outcomes are important but diverse and poorly captured in the current literature. The appropriateness of the patient-reported outcomes most commonly employed to measure physical activity is questionable and the range limited.
- The majority of patients feel better in relation to their ability to undertake physically active tasks including sports, but fail to progress to ‘feeling good’ or a patient-acceptable symptom state.

## Background

Hip arthroscopy is an increasingly common surgical intervention for young and middle-aged adults with hip-related pain or dysfunction [1–4]. Indications for hip arthroscopy most frequently include persistent pain and altered bony morphology associated with femoroacetabular impingement syndrome (FAIS) in addition to labral tears, chondral defects and ligamentum teres injuries [5, 6]. Young and middle-aged adults undergoing hip arthroscopy have high expectations for returning to physical activity to support their social and cultural roles [7]. Despite this expectation, physical activity-related outcomes are only reported in approximately a quarter of studies investigating surgical intervention for FAIS [8], returning to sport or play being the predominant outcome assessed. A high level of return to sport/ return to play following hip arthroscopy (88–91%) has been reported in a number of systematic reviews [9–16] ; however, recent study findings suggest the need for a more expansive analysis, beyond these simplified nominal criteria, to assess the wider impact of hip arthroscopy on physical activity. When adding the further consideration of level to sports status, Ishøi et al. [17] identified a relatively low return to pre-injury sport at pre-injury level of 57%, and Thorborg et al. [18] identified that at 1 year post-arthroscopy, only 25% of patients that met physical activity reference scores commensurate with those expected in a healthy population.

Dichotomous return-to-sport or return-to-play outcomes only provide a narrow perspective of physical activity which comprises multiple constructs such as the type, quantity, intensity and quality of activity, as well as physical activity-related impairments such as pain or discomfort. As these multiple dimensions imply, capturing comprehensive physical activity data is challenging and unlikely to be attained using a single measure [19]. One potential method of capturing data is through the use of patient-reported outcome measures (PROMs). Recommended PROMs with adequate clinometric properties for patients following hip arthroscopy include the Copenhagen Hip and Groin Outcome Score (HAGOS), International Hip Outcome Tool (iHOT-33) and Hip Outcome Score (HOS) [20–22]. While subscales of these PROMs primarily provide information on the degree of difficulty that patients experience with sport-related activities, other PROMs such as the Hip Sport Activity Scale (HSAS) provide information on the level of activity undertaken [23]. In addition to questionnaires, with advancing technology, potential exists to gather objective information relating to physical activity. Duration and intensity of physical activity may be captured through the use of motion sensors, accelerometry and mobile phone applications. Although an overview from ClinicalTrials.gov [24] lists over 1500 trials using accelerometry as an outcome measure, only 118 of these are related to musculoskeletal problems and less than 5 are related to the hip. The extent to which these newer technologies are being used and reported in relation to the outcomes following hip arthroscopic surgery has yet to be described.

To gain a comprehensive understanding of the impact of hip arthroscopy on the physical activity of patients, it is necessary to consider a range of outcomes and include both competitive and non-competitive (recreational) physical activity. Within the context of this review, physical activity is deemed to be an activity exceeding that which is required for normal activities of daily living, interpreting sport in a wider community context [25]. While arthroscopic interventions continue to evolve and increase in popularity [2, 4, 26], our current understanding of post-arthroscopy outcomes, in terms of physical activity, remains limited.

### Review Aim:

The primary aim of this systematic review is to examine quantitative primary research, reporting level IV evidence or above, to assess the impact of hip arthroscopy, undertaken for hip-related pain and dysfunction, on the physical activity of young and middle-aged adults. This will be assessed via the study outcomes presented. In addition, an overview of the outcomes used will be described.

## Methods

### Protocol and Registration

The protocol for this review was registered with the International Prospective Register of Systematic Reviews (PROSPERO, registration no. CRD42017080527). Amendments were made to the original protocol to (i) clarify exclusion criteria and (ii) modify outcomes in light of literature published during completion of the current review.

### Eligibility Criteria for Inclusion in the Review

Pre-specified inclusion and exclusion criteria are identified in Table 1.

**Table 1:** Inclusion and exclusion criteria

	Inclusion	Exclusion
Participants	18–50 years (Average age to fall in this range)	▪ Evidence of OA (> 10% of cohort with Tönnis grade 2 and above or joint space width of > 2 mm)
		▪ Dysplasia (LCEA mean for cohort < 20° &/or > 10% of the group with LCEA < 20°)
Intervention	Primary hip arthroscopy	▪ Secondary hip arthroscopy
		▪ Arthroscopy following hip joint arthroplasty
		▪ Studies in which arthroscopic and open procedures are combined
		▪ Studies in which primary focus is non-articular surgery
		▪ Studies in which periarticular osteotomy forms part of the procedure
Study types	Level IV evidence or above (RCT; prospective and retrospective observational studies)	▪ Case series < 5 participants
		▪ Published abstracts and non-peer-reviewed studies
		▪ Non-English language papers
Outcomes	Report change in physical activity and/or volume of sport participation	▪ Papers solely reporting prevalence of return to sport/return to play and/or sport-specific measures such as number of goals scored/career length
		▪ Return to work (including military service)
		▪ PROMs in which physical activity-related outcomes do not exceed normal activities of daily living

*OA* osteoarthritis, *LCEA* lateral centre edge angle, *PROM* patient-reported outcome measure, *RCT* randomised controlled trial

### Literature Search Strategy and Study Selection

A comprehensive search strategy was developed for the following databases: Scopus, MEDLINE, CINAHL, PubMed, AUSPORT, SPORTDiscus, PEDro and PsycINFO. The search was restricted to articles from January 1st 1990, due to the limited literature on hip arthroscopic surgery prior to this date, through to January 16th 2018. The search was updated through to December 5th 2019.

The search was conducted independently by two reviewers (DMJ, JJH), with the strategy adapted as appropriate for the requirements of each database. An example of the full search strategy is given in Additional file 1. Citation tracking of key articles was undertaken using Web of Science and Google Scholar. A manual check of reference lists of key articles was also undertaken. References were imported into Endnote X6 (Thomson Reuters, Carlsbad, California, USA) and duplicates removed. Title, abstract and full text screen were undertaken by two teams of independent reviewers (DMJ, JJH, BFM). Any disagreements were resolved by a fourth independent reviewer (JLK).

### Study appraisal

All included papers were assessed using an adaptation of the assessment form for observational studies created by Siegfried et al. [27], utilising further examples from Ganderton et al. [28, 29]. Copies of the appraisal form are given in Additional file 2. The tool considers biases relevant to observational studies in general and those specific to the research question. To address the research-specific biases, four authors (DMJ, JLK, KMC, JJH) compiled a list of potential confounding factors such as age, sex and the degree of degenerative change in the hip joint. As the majority of studies were non-randomised controlled trials, this approach was undertaken to align with good practice guidelines outlined by the non-randomised studies methods group of the Cochrane Collaboration [30]. This tool was used to assess methodological quality of all included studies by two teams of reviewers (DMJ, KD, MO, BM). Disagreements were resolved through discussion and, where necessary, consensus agreed with an independent arbitrator (JLK). Agreement between raters was determined using percentage-observed agreement and Cohen’s Kappa (κ). Itemisation and display of each aspect was presented in its raw form for each study. An assessment of level of evidence was made against the Oxford Centre for Evidence-Based Medicine criteria [31]

### Data extraction, synthesis and analyses

Data for each included study were extracted independently by two teams of reviewers (DJ, KD, MO, BM) using a standardised form adapted from the Cochrane Effective Practice and Organisation of Care (EPOC) criteria [32]. Inconsistencies were resolved by consensus discussion with arbitration from a third reviewer (JLK) if needed. Study authors were approached by email with requests for further data if required.

Data regarding study design, participant demographics (age, sex, physical activity attributes), outcome measures, duration of follow-up, arthroscopic findings and intervention were extracted and collated. The primary indication for surgery was noted (if specified). Where sufficient data were available, sports activities were categorised using previously established criteria in which activities are grouped based on the mechanical load placed on the hip joint (Table 2) [33, 34].

**Table 2:** Categories of sports activities, based on hip joint load

Category	Included activities
Cutting	Soccer, basketball, lacrosse, field hockey, downhill skiing, snowboarding
Flexibility	Dancing, gymnastics, yoga, cheerleading, figure skating, synchronized swimming, martial arts, rock climbing
Contact	Football, rugby, wrestling
Impingement	Ice hockey, crew/rowing, baseball catching, water polo, equestrian polo, breaststroke swimming, weight lifting, bobsled, crossfit, horseback riding
Asymmetric/overhead	Baseball, softball, tennis, golf, volleyball, athletic field events, fencing, badminton, cricket, squash, racquetball, handball
Endurance	Track, cross-country, other running, cycling, swimming (not breaststroke), cross-country skiing, biathlon, aerobics

To accommodate heterogeneity in the reporting of duration of follow-up, data collection points were collated under the following time frames: ≤ 6 months, 7–12 months, 13–18 months, 19–24 months, ≥ 25 months. Improvements in activity-specific subscales are known to be limited beyond 2 years post-arthroscopy [11, 35].

Reported outcomes were assessed to identify the direction and consistency of effect, and where appropriate data were available, standard paired differences (SPD) were calculated to present a magnitude of effect between time points. This was determined by the within-group difference between time points, divided by the pre-score standard deviation (SD). Where standard errors (SE) were reported, SD was calculated (SD = SE*√number of participants). The magnitude of SPDs was interpreted as large effect (≥ 0.8), moderate effect (0.5–0.79) and weak effect (0.2–0.49) [36]. The 95% confidence intervals for SPDs were calculated. Where appropriate summary scores were available for whole cohorts in studies with more than one arm, these data were used in preference to group data. Where data were insufficient for SPDs to be calculated, relevant study conclusions were reported where available.

To provide a visual representation of HOS-SS outcome scores, all data points from study groups were plotted against the minimal clinically important difference (MCID) and patient-acceptable symptom state (PASS) for this subscale (a change of 6 points and score of 75 points, respectively [21, 37, 38]). These scores were interpreted as ‘feeling better’ (MCID) and ‘feeling good’ (PASS) [39].

Pooling of data was undertaken where outcomes were statistically and clinically homogeneous. Any studies with potential replication of participants were excluded from this analysis. Where no responses were offered from authors to enable discrete cohorts to be identified, the study encompassing the widest time frame with the greatest number of participants was chosen from studies generated within the same research setting, utilising the same outcome measures and database. Where more than one outcome was reported in a study, the most frequently occurring outcome score across all studies was chosen to be reported in pooled data. Studies reporting number of participants or number of hips were included in the pooled data. Where reporting was unclear, a conservative approach was taken with calculations being made in relation to the lowest number of potential participants. Pooled data were examined using forest plots (Review Manager (RevMan) [Computer program]. Version 5.3. Copenhagen: The Nordic Cochrane Centre, The Cochrane Collaboration, 2014). Duration of follow-up categories were further merged to provide pooled data for the following time frames: 6 to 12 months, 13 to 24 months and ≥ 25 months. Studies were only reported once in each time frame.

## Results

### Search Strategy

**Fig. 1:**
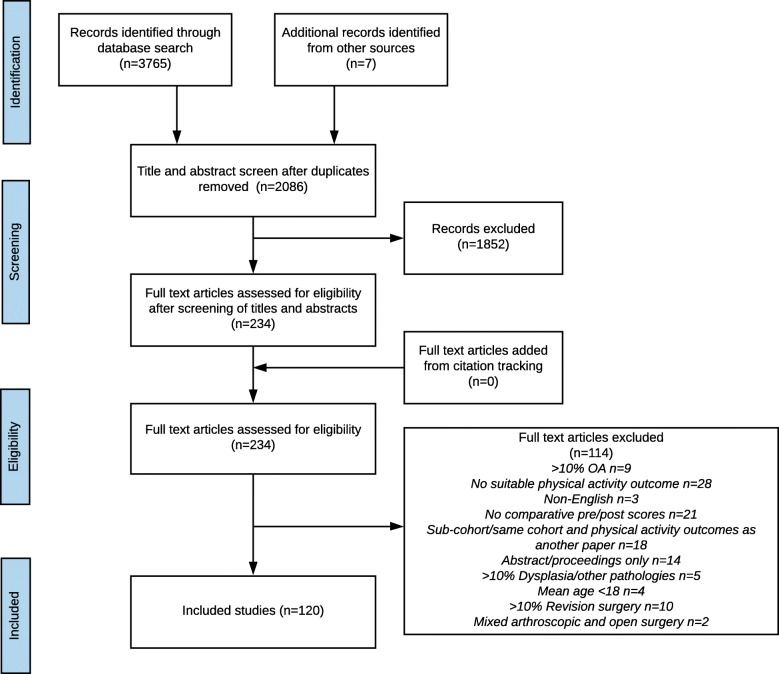
PRISMA flow chart

The number of records considered at each stage of the review and the reason for exclusions are shown in Fig. 1. In total, 120 studies were included in the review. A list of excluded studies is provided in Additional file 3.

### Study Characteristics

The included studies [6, 17, 18, 35, 37, 40–154] comprised two randomised controlled trials (RCTs), 24 prospective studies and 94 retrospective studies, of which 41 were single-arm case series (Additional file 4: Characteristics and outcomes of included studies). Author requests were made in relation to 51 (43%) studies to attain unreported data and query potential replication of participant data between studies. Additional information was supplied for five studies [18, 89, 99, 100, 112].

One hundred and twelve (93%) studies were conducted on a single site and/or involved the patients of one surgeon (Table 3). One hundred studies (83%) were from North America, 12 from Europe (10%) and 3 from Australia (2.5%). Three studies were from Korea, 1 from China and 1 from Israel.

**Table 3:** Summary of study quality assessment

			Internal Validity												
	External Validity		Performance	Detection				Attrition	Selection bias/control of confounding						
Study	Representative ✓	^1^Participation rate ✓	Direct observation ✓	PROM-validity/reliability ✓	^2^Direct measure - validity/ reliability	Blinded assessors ✓	^3^Outcome measure ✓	^1^Completeness ✓	^4^Age ✓	Location ✓	^5^Sex ✓	^6^Severity of Joint disease ✓	^7^Follow-up ✓	Single site &/or surgeon(YES)	LOE
RCTs															
n=2	2	2	2	2	NA	2	2	0	1	2	0	2	1	1	2
Prospective studies, more than 1 arm															
n=13	10	13	13	9	1	1	13	11	3	12	8	10	1	12	3
Prospective studies, Single-arm															
*n*=11	5	6	10	7	NA	1	11	8	2	11	3	7	1	9	3/4
Retrospective studies, more than1 arm															
n=53	41	48	53	2	NA	1	49	43	3	52	37	44	2	51	4
Retrospective studies, Single-arm															
n=41	32	40	40	6	NA	1	40	35	3	40	7	35	0	38	4

*RCTs* randomised controlled trials, *LOE* level of evidence (Oxford Centre for Evidence-Based Medicine [31]), *PROM* patient-reported outcome measure.

✓ indicates the measure was adequately addressed in the study

^1^✓ percent participation/ completion was 80% or more.

^2^NA indicates no direct measure of PA used

^3^✓ indicates same method of ascertainment was used for all participants

^4^✓ if range within 18–50

^5^✓ if sex is balanced (10% or less difference) or adjusted for in analysis

^6^✓ if severity of OA identified in the study

^7^✓ where FU is the same for all study participants or lies within 10%, i.e. the following acceptable ranges: 1 year follow-up = 1 month each way; 2 years follow-up = 2 months; 3 years follow-up = 3 months……10 years = 10 months

LOE=Level of evidence (Oxford Centre for Evidence-Based Medicine [31]); PROM=patient-reported outcome measure

A mix of reporting approaches was used, the majority of studies providing data based on participants (20,154 participants), the remainder recording 1,446 hips/procedures. We were unable to exclude the possibility of participants appearing in more than one study due to the high number of studies retrospectively reviewing databases. The number of participants in studies ranged from 11 to 1835. The mean (± SD) age of participants was 34 ± 7 years with 58% of the data pertaining to women. Seventy-two percent of studies specified FAI/FAIS as the primary inclusion pathology.

**Fig. 2:**
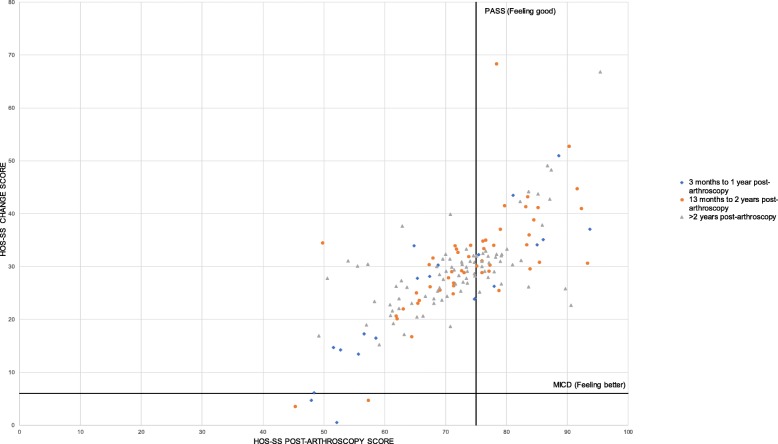
Hip Outcome Score-Sport Scale (HOS-SS) outcome scores for study groups at all time points. Points above the MCID (minimal clinically important difference) line represent a sufficient change in HOS-SS score pre- to post-arthroscopy to identify ‘feeling better’. Points to the right of the PASS (patient acceptable symptom state) represent a sufficiently high HOS-SS score at follow-up to identify ‘feeling better’

One study [154] reported objective measures of physical activity utilising accelerometry. The majority (*n* = 99, 83%) presented the Hip Outcome Score-sport-specific subscale (HOS-SS, Fig. 2). The ‘Function in Sport and Recreation subscale’, subscale of the Hip disability and Osteoarthritis Outcome score (HOOS-SS) and the two relevant subscales (‘Physical Function in Sport and Recreation’, ‘Participation in Physical Activities') of the Copenhagen Hip and Groin Outcome Scores (HAGOS-SR; HAGOS-PA) were presented in 8 (7%) and 8 (7%) of studies, respectively. An overview of PROMs is included in Additional file 5. The ‘Sports and Recreational Activities’ subscale of the International Hip Outcome Tool (iHOT-33 SR), Tegner Activity Scale (Tegner) and Hip Sports Activity Scale (HSAS) were reported in 2 (2%) of studies, while the UCLA Activity Score and Functional Activity Score (FAA) were each reported in a single study (Additional file 4). Outcome scores for studies with multiple time points of data collection can be found in Additional file 6. All but two studies reported pre- and post-arthroscopy results. Kemp et al. [89] provided an assessment of two post-arthroscopy time points; Tijssen et al. [124] reviewed changes from pre-injury to post-arthroscopy.

Thirty four (28%) of the reviewed studies included some assessment of physical activity attributes of the cohort such as type of activity (e.g. ‘recreational’, ‘professional’; work activity or Tegner Activity Scale) with a similar proportion providing sufficient data to enable categorisation of activity type (as identified in Table 2; *n* = 30, 25%). A summary of inclusion/exclusion criteria for each study, arthroscopic intervention and findings are given in Additional file 7.

### Quality assessment scores

Observed agreement between quality assessors was 99.6% (1554 out of 1560 items), where *κ* = 0.53, representing moderate inter-rater agreement [155].

All studies employed PROMs; however, the reporting of validity and reliability of these outcomes was deemed adequate in only 26 (22%) of the studies. Complete quality assessment scores are provided in Additional file 8 and a summary is provided in Table 3. Blinding of those assessing data was poorly addressed in all but six studies (5%) and only six studies (5%) provided clearly identifiable time points in which all follow-up outcomes related to analogous time frames. Although the mean age of participants in all studies met the current inclusion criteria, 108 studies (90%) included some participants outside this age range or failed to report sufficient information.

### Main Findings

Large effect sizes for patient-reported physical activity (where able to be calculated) were seen in all studies at latest follow-up for the HOS, HOOS, HAGOS and iHOT33 subscales, with the exception of ten study groups for the HOS [44, 80, 85, 97, 98, 138, 142, 144, 146, 147]; and one for HAGOS [17] in which effect sizes were moderate pre- to post-arthroscopy. In assessing progress between two post-arthroscopy time points, Kemp et al [89] determined a small effect size for the HOOS-SR. The direction of change was consistently toward improvement across studies. Table 4 shows the summary of the range (minimum SPD and maximum SPD) of effect sizes for each score across all studies for individual outcomes. The full set of results of SPDs are contained in Additional file 4.

**Table 4:** Range of effect sizes for each instrument across all studies (pre- to post-arthroscopy)

Measure	Study	Number (*n*)	Follow-up period	*SPD (95% CI)
HOS-SS	Wu et al .[128] Rhee et al. [115]	68 37	≥ 25 months 7-12 months	− 5.27 (− 5.98 to − 4.55) -0.52 [-0.98 to -0.05]
HOOS-SR	Flores et al. [70]	39	7 to 12 months	-2.02[-2.57 to -1.47]
	Ibrahim et al. [85]	88	≥25 months	-0.63[-0.93 to -0.32]
HAGOS-SR	Bennell et al.[Group 1] [131] Ishoi et al. [17]	11 108	≤6 months≥ 25 months	-2.21 [-3.24 to -1.17] -0.66 [-0.93 to -0.38]
HAGOS-PA	Sansone et al. [118] Lund et al. [99]	85 1835	7 to 12 months 21 to 42 months	-1.48 [-1.82 to -1.14] -0.85 [-0.92 to -0.78]
HSAS	Lund et al. [99] Bennell et al. [131]	1835 11	21 to 42 months ≤6 months	-0.41 [-0.48 to -0.34] 0 [-0.79 to 0.79]
Tegner	Bennell et al. [Group 1] [131] Bennell et al. [Group 2] [131]	11 11	≤6 months ≤6 months	-0.9 [-1.74 to -0.07] -0.64 [-1.43 to 0.15]

*n* number of participants, *SPD* standard paired difference, *CI* confidence interval, *HOS-SS* Hip Outcome Score-Sport Scale, *HOOS-SR* Hip Disability and Osteoarthritis Outcome Score-Function in Sport and Recreation, *HAGOS-SR/PA* The Copenhagen Hip and Groin Outcome Score-Physical Function in Sport and Recreation / Participation in Physical Activities, *HSAS* Hip Sports Activity Scale, *Tegner* Tegner Activity Scale,

*Interpreted as large effect (≥ 0.8), moderate effect (0.5–0.79), and weak effect (0.2–0.49) [36]

Pre- to post-arthroscopy change in the HSAS was assessed in four studies [6, 99, 118, 131]. No effect and small effect were evident at 6 months post-arthroscopy in the RCT conducted by Bennell et al. [131] compared to a moderate effect size at 6 months post-arthroscopy reported by Sansone et al. [118] (SPD [95% CI]; 0 [−0.79 to 0.79]; 0.12 [−0.89 to 0.65]; −0.63 [−0.94 to 0.33] respectively). Two studies [6, 99] showed small effect sizes at approximately 2 years (SPD [95% CI]; −0.33 [−0.49 to 0.16]; −0.41 [−0.48 to 0.34]). Bennell et al. [131] was the only study to assess pre- and post-arthroscopy Tegner scores, finding large-to-moderate effect sizes at 6 months post-arthroscopy (SPD [95% CI]; −0.90 [−1.74 to 0.07]; −0.64 [−1.43 to 0.15]).

A visual representation of all HOS-SS outcome scores is presented in Fig. 2. Two studies [49, 100] had outcome scores sitting below the MCID and PASS scores (3% of all included data points). Sixty percent of outcome data points failed to reach the magnitude required to reach the PASS score. For data points relating to a follow-up duration of ≥ 25 months, 64% failed to reach the PASS score.

**Fig. 3:**
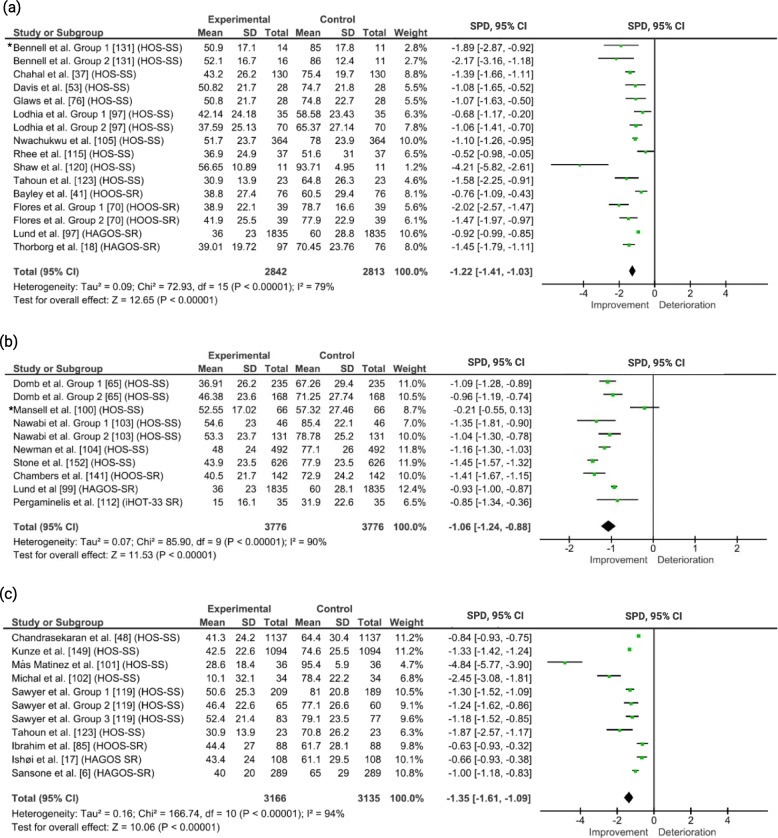
Pooled effect sizes of pre- to post-arthroscopy including Hip Outcome Score-Sport Scale (HOS-SS), Hip disability and Osteoarthritis Outcome Score-Function in Sport and Recreation (HOOS-SR), The Copenhagen Hip and Groin Outcome Score-Physical Function in Sport and Recreation (HAGOS-SR) and International Hip Outcome Tool-Sports and Recreational activities (iHOT-33 SR) at 6−12 months (**a**); 13−24 months post-arthroscopy (**b**) and ≥ 25 months (**c**), showing standard paired difference (SPD) and 95% confidence intervals (CI). Weightings relate to study size. Randomised controlled trials are indicated with *

Data were pooled for HOS-SS, HOOS-SR, HAGOS SR and iHOT-33 SR and grouped according to time frame (Fig. 3). A large effect was evident for SPDs at each time frame (SPD [95% CI]; −1.22 [-1.41 to −1.03]; −1.06 [−1.24 to −0.88] and −1.35 [−1.61 to −1.09] at 6–12 months, 13–24 months and ≥ 25 months, respectively). Considerable heterogeneity was evident between studies in all time frames (*I*^2^ 79% to 92%).

Eight studies [73–75, 95, 116, 124, 126, 154] reported quantified changes in physical activity. Methods used in these studies were largely sport-specific, e.g. change in swimming distances pre- to post-arthroscopy [74] or number of holes of golf played per week [126]. Decreases were evident in all measures, although this change was not significantly different in five of the studies [73–75, 116, 126]. Significant decreases were reported in running mileage [95] (*P* < 0.001) and sport frequency [124] pre-injury to post-arthroscopy. Kierkegaard et al [154] identify a self-reported four-fold increase in hours of physical activity per week but no significant differences were reported for accelerometry-derived activity data such as the percentage of time spent in undertaking moderate or high physical activity, step count or percentage of time running between pre-arthroscopy and 1-year post-arthroscopy (Additional file 4).

## Discussion

This systematic review evaluated the impact of hip arthroscopy, undertaken for hip-related pain and dysfunction, on the physical activity of young and middle-aged adults. A limited range of relevant outcomes were reported, with PROMs, specifically the HOS-SS predominating, and one study using objective measures to monitor physical activity. Consistency was seen across PROMs for improvements post-arthroscopy; however, the majority of HOS-SS scores did not reflect a patient-acceptable symptom state. In interpreting the evidence, it should be noted that considerable heterogeneity was evident between study designs and eligibility criteria. The majority of studies (78%) were retrospective, the preponderance of level 4 evidence, thus having the potential to inflate positive outcomes and effect sizes.

Pooled data showed large effect sizes for the PROM subscales included in the analysis (HOS-SS, HAGOS-SR, iHOT-33 SR), depicting improvements in patients’ perceived difficulties with sport-related activities. This was consistent within each time frame for data covering 6 to ≥ 25 months post-arthroscopy. Across all pooled data, four studies demonstrated extreme positive effects. Three of these studies [54, 101, 120] involved participants undertaking high-level physical activity with elevated post-arthroscopy scores. Conversely, Michal et al. [102] reported very low pre-arthroscopy scores in a cohort who underwent surgery for subspinal decompression. Excluding these studies from the analysis did not impact on the large pooled effect sizes. While the pooled data reflect a positive trend of patient-reported improvements in relation to physical activity impairments, isolated analysis of the HOS-SS raised questions about whether the magnitude of improvement was sufficient to be perceived by patients as satisfactory recovery of physical activity. The failure of 64% of reported HOS-SS scores to meet the PASS level for this scale beyond 2 years post-arthroscopy, echoes previously identified deficits in the HAGOS-SR and HAGOS-PA scores for patients at 1 year post-arthroscopy compared to their healthy peers [18]. These findings should encourage clinicians to monitor and support patients’ return to physical activity for extended time spans following hip arthroscopy. The heterogeneity of the study cohorts, in relation to number of participants, age range, diagnosis, surgical procedures, physical activity background and time point at which data were gathered, potentially underlies the spread of outcomes depicted in Fig. 2, although this speculation also requires further investigation into the suitability of the outcome measure for the population.

Our findings indicate the need for more in-depth analysis of the impact of surgery on sport and activity involvement at an individual level. The limited range of outcomes utilised within studies was insufficient to answer questions about how much activity patients are undertaking and at what level of involvement. Despite the rising interest in and accessibility of wearable technology in health and fitness [156], and the increasing use of activity monitors within health research [24, 157], we found only one study utilising objective monitoring of physical activity for hip arthroscopy patients. Without the collection of more robust data to identify the type and quantity of activity undertaken, we are unable to determine if patients are participating in sufficient physical activity to meet guidelines of minimal activity requirements for health.

The limited range of frequently used PROMs identified in the current review reflects the findings of Reiman et al. [8] and Renouf et al. [158]. Both these reviews identified that PROMs with appropriate clinimetric evidence to support their use in the population of young to middle-aged adults with hip-related pain and dysfunction, such as the iHOT-33 and the HAGOS, were utilised in less than 5% of studies assessing outcomes following hip arthroscopy and surgery for FAIS. The utility of the HOS-SS in this population has yet to be clearly established. In a recent review of PROMs for hip-related pain [20], the HOS was not recommended as it lacked content validity, an issue that likely also applies to the individual subscales. As Kemp et al. [21] also observed ceiling effects for the HAGOS-PA subscale, limiting its ability to identify improvements over time in hip-arthroscopy patients, further research is needed to identify which PROMs are best suited to capture physical activity gains in this cohort. PROMs that provide information on levels of activity, such as the HSAS and the Tegner were also infrequently utilised. The HSAS was assessed in four studies [6, 99, 118, 131], identifying no to moderate effect at 6 months [118, 131] and small effect sizes at approximately 2 years post-arthroscopy [6, 99]. Although the number of studies is limited, the smaller effect sizes may be indicative of less profound changes in relation to improvements in activity levels following surgery. Similarly, although only seven of the included studies sought to quantify the amount of activity undertaken in specific sports, the negative trends depicted indicate the importance of tracking more than one domain of physical activity. This is reiterated in the findings of Kierkegaard et al. [154], with the lack of agreement between objective and subjective reports of activity change. Only a quarter of the studies reported on the activity profile of participants, although information about the type of activity undertaken would be of value in identifying potential barriers and facilitators to physical activity participation post-arthroscopy.

This study offers insights into the effect of hip arthroscopy on physical activity, based on a comprehensive search strategy across eight databases utilising a rigorous screening and review process; however, there are a number of limitations that should be acknowledged. The methodological quality of the included studies was variable, many being retrospective studies with low participant numbers. This may increase potential for bias and magnification of positive effects [159]. Additionally, a number of studies were based on reviews of archived databases. The reliability of evidence emanating from these sources depends upon the quality of the database. National registries such as those developed in Sweden and Denmark, for which criteria, planning, monitoring and ongoing quality assurance are transparent [3, 160], provide data with high external validity. While single site/ single-surgeon registries offer a convenient tool for internal audit, the external validity and applicability of these data in the wider field are limited. When pooling study data in this review, a conservative approach was taken to data that were potentially derived from same database. While this reduced the number of studies contributing to the pooled data, it minimised the potential for data from the same participant to be duplicated in the analysis. It should be noted that in the visual representation of all HOS-SS outcomes, all studies were included. The high incidence of the HOS-SS may be an artefact of the number of studies emanating from North America and the dominance of a limited number of surgical centres, exacerbated by the omission of non-English language studies in this review. The predominance of North American studies also limits the cultural perspective of the data, with potential biases arising from influences on the manner in which participants complete patient-reported outcomes.

## Conclusion

The current level of information regarding physical activity for post-arthroscopy patients is limited in scope. Within the framework of patients’ perceived difficulties with sport-related activities, there is a consistent trend of post-arthroscopy improvement. However, the limited percentage of study participants achieving a score commensurate with ‘feeling good’, rather than ’feeling better’, indicates a need for more in-depth analysis to identify potential barriers and facilitators, both physical and psychological, to achieving a more satisfactory return to physical activity.

Although the HOS-SS was the most frequently utilised PROM in this review, questions remain regarding its utility for this cohort. A greater range of outcome measures is needed to identify changes in other domains of physical activity. The use of objective measures, such as step count data, is currently a resource that is rarely utilised in studies, despite its use in contemporary practice, and warrants further investigation.

This review generates a compelling case for higher quality, sufficiently powered observational studies and RCTs. While RCTs remain the gold standard, purposefully designed, quality controlled, multicentre or population-level databases offer the opportunity for large-scale, comprehensive data collection. However, a more expansive view of physical activity profiles needs to be established with the routine collection of data about type and volume of physical activity undertaken beyond the traditional focus on ‘sport’-related physical activity.

## Supplementary information

**Additional file 1:** Example search strategy.

**Additional file 2:** Study quality assessment forms.

**Additional file 3:** Studies excluded at full text screen.

**Additional file 4:** Characteristics and outcomes of included studies.

**Additional file 5:** Overview of patient-reported outcomes identified in the review.

**Additional file 6:** Data from intermediate time points.

**Additional file 7:** Inclusion criteria, exclusion criteria, arthroscopic findings and interventions.

**Additional file 8:** Risk of bias assessment.

## Data Availability

Supporting data are supplied as electronic supplementary material and referred to within the script.

## Abbreviations

PROMs: Patient-reported outcome measures
HAGOS-PA: The Copenhagen Hip and Groin Outcome Score-Participation in Physical Activities
HAGOS-SR: The Copenhagen Hip and Groin Outcome Score-Physical Function in Sport and Recreation
HOOS-SR: Hip disability and Osteoarthritis Outcome Score-Function in Sport and Recreation
HOS-SS: Hip Outcome Score-Sport Scale
HSAS: Hip Sports Activity Scale
iHOT-33 SR: International Hip Outcome Tool-Sports and Recreational activities
NAHS: Non-Arthritic Hip Score
HHS: Harris Hip Score
mHHS: Modified Harris Hip Score
Tegner: Tegner Activity Scale
UCLA: The University of California at Los Angeles activity score

## References

[1] Griffin D, Wall P, Realpe A, Adams A, Parsons N, Hobson R, et al. UK FASHIoN: feasibility study of a randomised controlled trial of arthroscopic surgery for hip impingement compared with best conservative care. Health Technol Assess. 2016;20(32) 10.3310/hta20320. 10.3310/hta20320 PMC4860559 27117505

[2] Montgomery SR, Ngo SS, Hobson T, Nguyen S, Alluri R, Wang JC (2013). Trends and demographics in hip arthroscopy in the United States. Arthroscopy..

[3] Mygind-Klavsen B, Gronbech Nielsen T, Maagaard N, Kraemer O, Holmich P, Winge S (2016). Danish Hip Arthroscopy Registry: an epidemiologic and perioperative description of the first 2000 procedures. J Hip Preserv Surg..

[4] Palmer A, Malak T, Broomfield J, Holton J, Majkowski L, Thomas G, et al. Past and projected temporal trends in arthroscopic hip surgery in England between 2002 and 2013. BMJ Open Sport Exerc Med. 2016;2. 10.1136/bmjsem-2015-000082 OI 10.1136/bmjsem-2015-000082 PMC5117047 27900161

[5] Kelly BT, Williams RJ, Philippon MJ (2003). Hip arthroscopy: current indications, treatment options, and management issues. Am J Sports Med..

[6] Sansone M, Ahldén M, Jónasson P, Thomeé C, Swärd L, Öhlin A (2017). Outcome after hip arthroscopy for femoroacetabular impingement in 289 patients with minimum 2-year follow-up. Scand J Med Sci Sports..

[7] Mannion A, Impellizzeri F, Naal F, Leunig M (2013). Fulfilment of patient-rated expectations predicts the outcome of surgery for femoroacetabular impingement. Osteoarthritis Cartilage..

[8] Reiman MP, Peters S, Sylvain J, Hagymasi S, Ayeni OR (2018). Prevalence and consistency in surgical outcome reporting for femoroacetabular impingement syndrome: a scoping review. Arthroscopy..

[9] Alradwan H, Philippon MJ, Farrokhyar F, Chu R, Whelan D, Bhandari M (2012). Return to preinjury activity levels after surgical management of femoroacetabular impingement in athletes. Arthroscopy..

[10] Casartelli NC, Leunig M, Maffiuletti NA, Bizzini M (2015). Return to sport after hip surgery for femoroacetabular impingement: a systematic review. Br J Sports Med..

[11] Kierkegaard S, Langeskov-Christensen M, Lund B, Naal FD, Mechlenburg I, Dalgas U (2017). Pain, activities of daily living and sport function at different time points after hip arthroscopy in patients with femoroacetabular impingement: a systematic review with meta-analysis. Br J Sports Med..

[12] Lovett-Carter D, Jawanda AS, Hannigan A. Meta-analysis of the surgical and rehabilitative outcomes of hip arthroscopy in athletes with femoroacetabular impingement. Clin J Sport Med. 2018; 10.1097/JSM.0000000000000623. 10.1097/JSM.0000000000000623 29933279

[13] Memon M, Kay J, Hache P, Simunovic N, Harris JD, O’Donnell J, et al. Athletes experience a high rate of return to sport following hip arthroscopy. Knee Surg Sports Traumatol Arthrosc. 2018; 10.1007/s00167-018-4929-z. 10.1007/s00167-018-4929-z 29627931

[14] Minkara AA, Westermann RW, Rosneck J, Lynch TS (2019). Systematic review and meta-analysis of outcomes after hip arthroscopy in femoroacetabular impingement. Am J Sports Med..

[15] O’Connor M, Minkara AA, Westermann RW, Rosneck J, Lynch TS (2018). Return to play after hip arthroscopy: a systematic review and meta-analysis. Am J Sports Med..

[16] Reiman MP, Peters S, Sylvain J, Hagymasi S, Mather RC, Goode AP (2018). Femoroacetabular impingement surgery allows 74% of athletes to return to the same competitive level of sports participation but their level of performance remains unreported: a systematic review with meta-analysis. Br J Sports Med..

[17] Ishøi L, Thorborg K, Kraemer O, Hölmich P (2018). Return to sport and performance after hip arthroscopy for femoroacetabular impingement in 18-to 30-year-old athletes: a cross-sectional cohort study of 189 athletes. Am J Sports Med..

[18] Thorborg K, Kraemer O, Madsen A-D, Hölmich P (2018). Patient-reported outcomes within the first year after hip arthroscopy and rehabilitation for femoroacetabular impingement and/or labral injury: the difference between getting better and getting back to normal. Am J Sports Med..

[19] Kelly P, Fitzsimons C, Baker G. Should we reframe how we think about physical activity and sedentary behaviour measurement? Validity and reliability reconsidered. Int J Behav Nutr Phys Act. 2016;13(32). 10.1007/s00167-018-4929-z10.1186/s12966-016-0351-4 10.1186/s12966-016-0351-4 PMC4772314 26931142

[20] Impellizerri FM, Jones DM, Griffin D, Harris Hayes M, Thorborg K, Crossley KM, et al. Patient-reported outcome measures for hip-related pain: a review of the available evidence and a Consensus Statement from the International Hip-related Pain Research Network, Zurich 2018. Br J Sports Med. 2019 (awaiting publication). 10.1136/bjsports-2019-101456 32066573

[21] Kemp JL, Collins NJ, Roos EM, Crossley KM (2013). Psychometric properties of patient-reported outcome measures for hip arthroscopic surgery. Am J Sports Med..

[22] Thorborg K, Tijssen M, Habets B, Bartels E, Roos EM, Kemp J (2015). Patient-reported outcome (PRO) questionnaires for young-aged to middle-aged adults with hip and groin disability: a systematic review of the clinimetric evidence. Br J Sports Med..

[23] Naal FD, Miozzari HH, Kelly BT, Magennis EM, Leunig M, Noetzli HP (2013). The Hip Sports Activity Scale (HSAS) for patients with femoroacetabular impingement. Hip Int..

[24] National Library of Medicine (US). Clinical trials data base. 2019 https://clinicaltrials.gov. Accessed 9 Dec 2019

[25] Finch CF (1997). An overview of some definitional issues for sports injury surveillance. Sports Med..

[26] Kremers HM, Schilz SR, Van Houten HK, Herrin J, Koenig KM, Bozic KJ (2017). Trends in utilization and outcomes of hip arthroscopy in the United States between 2005 and 2013. J Arthroplasty..

[27] Siegfried N, Muller M, Deeks J, Volmink J, Egger M, Low N (2005). HIV and male circumcision—a systematic review with assessment of the quality of studies. Lancet Infect Dis..

[28] Ganderton C, Pizzari T (2013). A systematic literature review of the resistance exercises that promote maximal muscle activity of the rotator cuff in normal shoulders. Shoulder & Elbow..

[29] Ganderton C, Semciw A, Cook J, Pizzari T (2016). The effect of female sex hormone supplementation on tendon in pre and postmenopausal women: a systematic review. J Musculoskelet Neuronal Interact..

[30] Reeves B, Deeks J, Higgins J, Wells G. Including non-randomized studies. In: Higgins J, Green S, editors. Cochrane Handbook for Systematic Reviews of Interventions Version 510 (updated March 2011). The Cochrane Collaboration; 2011. http://www.handbook.cochrane.org. .

[31] OCEBM Levels of Evidence Working group. The Oxford 2011 levels of evidence. 2011; http://www.cebm.net/index.aspx?o=5653. .

[32] Cochrane Effective Practice and Organisation of Care (EPOC). Data collection form. 2017; http://epoc.cochrane.org/resources/epoc-specific-resources-review-authors. Accessed 19 October 2017.

[33] Nawabi DH, Bedi A, Tibor LM, Magennis E, Kelly BT (2014). The demographic characteristics of high-level and recreational athletes undergoing hip arthroscopy for femoroacetabular impingement: a sports-specific analysis. Arthroscopy..

[34] Shibata KR, Matsuda S, Safran MR (2017). Arthroscopic hip surgery in the elite athlete: comparison of female and male competitive athletes. Am J Sports Med..

[35] Flores SE, Sheridan JR, Borak KR, Zhang AL. When do patients improve after hip arthroscopy for femoroacetabular impingement? A prospective cohort analysis. Am J Sports Med. 2018;46(13):3111–8. 10.1177/0363546518795696 30226992

[36] Cohen J. Statistical power analyses for the social sciences. 2nd ed: Hillsdale, NJ, Lawrence Erlbauni Associates; 1988.

[37] Chahal J, Van Thiel GS, Mather RC, Lee S, Song SH, Davis AM (2015). The patient acceptable symptomatic state for the modified harris hip score and hip outcome score among patients undergoing surgical treatment for femoroacetabular impingement. Am J Sports Med..

[38] Martin RL, Philippon MJ (2008). Evidence of reliability and responsiveness for the hip outcome score. Arthroscopy..

[39] Tubach F, Dougados M, Falissard B, Baron G, Logeart I, Ravaud P (2006). Feeling good rather than feeling better matters more to patients. Arthrit Care Res..

[40] Barastegui D, Seijas R, Alvarez-Diaz P, Rivera E, Alentorn-Geli E, Steinbacher G (2018). Assessing long-term return to play after hip arthroscopy in football players evaluating risk factors for good prognosis. Knee Surg Sports Traumatol Arthrosc..

[41] Bayley G, Poitras S, Parker G, Beaule PE (2017). Hip arthroscopy in patients less than 25 years of age in the treatment of labral tears: aetiology and clinical outcomes. Hip Int.

[42] Bennett AN, Nixon J, Roberts A, Barker-Davies R, Villar R, Houghton JM. Prospective 12-month functional and vocational outcomes of hip arthroscopy for femoroacetabular impingement as part of an evidence-based hip pain rehabilitation pathway in an active military population. BMJ Open Sport Exerc Med. 2016;2(1): 10.1136/bmjsem-2016-000144. 10.1136/bmjsem-2016-000144 PMC5117082 27900190

[43] Chaharbakhshi EO, Perets I, Ashberg L, Mu B, Lenkeit C, Domb BG (2017). Do ligamentum teres tears portend inferior outcomes in patients with borderline dysplasia undergoing hip arthroscopic surgery? A match-controlled study with a minimum 2-year follow-up. Am J Sports Med..

[44] Chandrasekaran S, Darwish N, Chaharbakhshi EO, Suarez-Ahedo C, Lodhia P, Domb BG (2017). Minimum 2-year outcomes of hip arthroscopic surgery in patients with acetabular overcoverage and profunda acetabulae compared with matched controls with normal acetabular coverage. Am J Sports Med..

[45] Chandrasekaran S, Darwish N, Close MR, Lodhia P, Suarez-Ahedo C, Domb BG (2017). Arthroscopic reconstruction of segmental defects of the hip labrum: results in 22 patients with mean 2-year follow-up. Arthroscopy..

[46] Chandrasekaran S, Darwish N, Martin TJ, Suarez-Ahedo C, Lodhia P, Domb BG (2017). Arthroscopic capsular plication and labral seal restoration in borderline hip dysplasia: 2-year clinical outcomes in 55 cases. Arthroscopy..

[47] Chandrasekaran S, Gui C, Darwish N, Lodhia P, Suarez-Ahedo C, Domb BG (2016). Outcomes of hip arthroscopic surgery in patients with Tonnis grade 1 osteoarthritis with a minimum 2-year follow-up: evaluation using a matched-pair analysis with a control group with Tonnis grade 0. Am J Sports Med..

[48] Chandrasekaran S, Gui C, Walsh JP, Lodhia P, Suarez-Ahedo C, Domb BG. Correlation between changes in Visual Analog Scale and patient-reported outcome scores and patient satisfaction after hip arthroscopic surgery. Orthop J Sports Med. 2017;5(9). 10.1177/2325967117724772 10.1177/2325967117724772 PMC5600309 28932750

[49] Chandrasekaran S, Vemula SP, Lindner D, Lodhia P, Suarez-Ahedo C, Domb BG (2015). Preoperative delayed gadolinium-enhanced magnetic resonance imaging of cartilage (dGEMRIC) for patients undergoing hip arthroscopy: indices are predictive of magnitude of improvement in two-year patient-reported outcomes. J Bone Joint Surg Am..

[50] Chen AW, Yuen LC, Ortiz-Declet V, Litrenta J, Maldonado DR, Domb BG (2018). Selective debridement with labral preservation using narrow indications in the hip: minimum 5-year outcomes with a matched-pair labral repair control group. Am J Sports Med..

[51] Cvetanovich GL, Levy DM, Weber AE, Kuhns BD, Mather RC, Salata MJ (2017). Do patients with borderline dysplasia have inferior outcomes after hip arthroscopic surgery for femoroacetabular impingement compared with patients with normal acetabular coverage?. Am J Sports Med..

[52] Cvetanovich GL, Weber AE, Kuhns BD, Alter J, Harris JD, Mather RC (2018). Hip arthroscopic surgery for femoroacetabular impingement with capsular management: factors associated with achieving clinically significant outcomes. Am J Sports Med..

[53] Davis CC, Ellis TJ, Amesur AK, Hewett TE, Di Stasi S (2016). Improvements in knee extension strength are associated with improvements in self-reported hip function following arthroscopy for femoroacetabular impingement syndrome. Int J Sports Phys Ther..

[54] Degen RM, Fields KG, Wentzel CS, Bartscherer B, Ranawat AS, Coleman SH (2016). Return-to-play rates following arthroscopic treatment of femoroacetabular impingement in competitive baseball players. Phys Sportsmed..

[55] Degen RM, Nawabi DH, Fields KG, Wentzel CS, Kelly BT, Coleman SH (2016). Simultaneous versus staged bilateral hip arthroscopy in the treatment of femoroacetabular impingement. Arthroscopy..

[56] Domb BG, Chaharbakhshi EO, Perets I, Walsh JP, Yuen LC, Ashberg LJ (2018). Patient-reported outcomes of capsular repair versus capsulotomy in patients undergoing hip arthroscopy: minimum 5-year follow-up-a matched comparison study. Arthroscopy..

[57] Domb BG, Chaharbakhshi EO, Perets I, Yuen LC, Walsh JP, Ashberg L (2018). Hip arthroscopic surgery with labral preservation and capsular plication in patients with borderline hip dysplasia: minimum 5-year patient-reported outcomes. American Journal of Sports Medicine..

[58] Domb BG, Close MR, Litrenta J, Perets I, Chaharbakhshi EO, Rybalko D (2017). Outcomes of hip arthroscopic surgery in patients with Tönnis grade 1 osteoarthritis at a minimum 5-year follow-up: a matched-pair comparison with a Tönnis grade 0 control group. Am J Sports Med..

[59] Domb BG, Dunne KF, Martin TJ, Gui C, Finch NA, Vemula SP (2016). Patient reported outcomes for patients who returned to sport compared with those who did not after hip arthroscopy: minimum 2-year follow-up. J Hip Preserv Surg..

[60] Domb BG, El Bitar YF, Lindner D, Jackson TJ, Stake CE (2014). Arthroscopic hip surgery with a microfracture procedure of the hip: clinical outcomes with two-year follow-up. Hip Int..

[61] Domb BG, Gui C, Hutchinson MR, Nho SJ, Terry MA, Lodhia P (2016). Clinical outcomes of hip arthroscopic surgery: a prospective survival analysis of primary and revision surgeries in a large mixed cohort. Am J Sports Med..

[62] Domb BG, Linder D, Finley Z, Botser IB, Chen A, Williamson J (2015). Outcomes of hip arthroscopy in patients aged 50 years or older compared with a matched-pair control of patients aged 30 years or younger. Arthroscopy..

[63] Domb BG, Rybalko D, Mu B, Litrenta J, Chen AW, Perets I (2018). Acetabular microfracture in hip arthroscopy: clinical outcomes with minimum 5-year follow-up. Hip Int..

[64] Domb BG, Stake CE, Botser IB, Jackson TJ (2013). Surgical dislocation of the hip versus arthroscopic treatment of femoroacetabular impingement: a prospective matched-pair study with average 2-year follow-up. Arthroscopy..

[65] Domb BG, Stake CE, Finley ZJ, Chen T, Giordano BD (2015). Influence of capsular repair versus unrepaired capsulotomy on 2-year clinical outcomes after arthroscopic hip preservation surgery. Arthroscopy..

[66] Domb BG, Stake CE, Lindner D, El-Bitar Y, Jackson TJ (2013). Arthroscopic capsular plication and labral preservation in borderline hip dysplasia: two-year clinical outcomes of a surgical approach to a challenging problem. Am J Sports Med..

[67] Domb BG, Yuen LC, Ortiz-Declet V, Litrenta J, Perets I, Chen AW (2017). Arthroscopic labral base repair in the hip: 5-year minimum clinical outcomes. Am J Sports Med..

[68] Fabricant PD, Fields KG, Taylor SA, Magennis E, Bedi A, Kelly BT (2015). The effect of femoral and acetabular version on clinical outcomes after arthroscopic femoroacetabular impingement surgery. J Bone Joint Surg Am..

[69] Flores SE, Borak KR, Zhang AL. Hip arthroscopic surgery for femoroacetabular impingement: a prospective analysis of the relationship between surgeon experience and patient outcomes. Orthop J Sports Med. 2018;6(2). 10.1177/2325967118755048 10.1177/2325967118755048 PMC5815420 29468172

[70] Flores SE, Chambers CC, Borak KR, Zhang AL. Arthroscopic treatment of acetabular retroversion with acetabuloplasty and subspine decompression: a matched comparison with patients undergoing arthroscopic treatment for focal pincer-type femoroacetabular impingement. Orthop J Sports Med. 2018;6(7). 10.1177/2325967118783741 10.1177/2325967118783741 PMC6055268 30046631

[71] Frank RM, Lee S, Bush-Joseph CA, Kelly BT, Salata MJ, Nho SJ (2014). Improved outcomes after hip arthroscopic surgery in patients undergoing t-capsulotomy with complete repair versus partial repair for femoroacetabular impingement: a comparative matched-pair analysis. Am J Sports Med..

[72] Frank RM, Lee S, Bush-Joseph CA, Salata MJ, Mather RC, Nho SJ (2016). Outcomes for hip arthroscopy according to sex and age. J Bone Joint Surg..

[73] Frank RM, Ukwuani G, Allison B, Clapp I, Nho SJ (2018). High rate of return to yoga for athletes after hip arthroscopy for femoroacetabular impingement syndrome. Sports Health..

[74] Frank RM, Ukwuani G, Chahla J, Batko B, Bush-Joseph CA, Nho SJ (2018). High rate of return to swimming after hip arthroscopy for femoroacetabular impingement. Arthroscopy..

[75] Frank RM, Ukwuani G, Clapp I, Chahla J, Nho SJ (2017). High rate of return to cycling after hip arthroscopy for femoroacetabular impingement syndrome. Sports Health..

[76] Glaws KR, Ellis TJ, Hewett TE, Di Stasi S (2019). Return to sport rates in physically active individuals six months after arthroscopy for femoroacetabular impingement syndrome. J Sport Rehabil..

[77] Gupta A, Redmond JM, Hammarstedt JE, Stake CE, Domb BG (2015). Does obesity affect outcomes in hip arthroscopy? A matched-pair controlled study with minimum 2-year follow-up. Am J Sports Med..

[78] Gupta A, Redmond JM, Stake CE, Dunne KF, Domb BG (2016). Does primary hip arthroscopy result in improved clinical outcomes? 2-year clinical follow-up on a mixed group of 738 consecutive primary hip arthroscopies performed at a high-volume referral center. Am J Sports Med..

[79] Hartigan DE, Perets I, Chaharbakhshi EO, Walsh JP, Yuen LC, Domb BG (2018). Outcomes of femoral head marrow stimulation techniques at minimum 2-year follow-up. Orthopedics..

[80] Hartigan DE, Perets I, Walsh JP, Chaharbakhshi EO, Yuen LC, Domb BG. Clinical outcomes of hip arthroscopic surgery in patients with femoral retroversion: a matched study to patients with normal femoral anteversion. Orthop J Sports Med. 2017;5(10). 10.1177/2325967117732726 10.1177/2325967117732726 PMC5661671 29124076

[81] Hartigan DE, Perets I, Walsh JP, Close MR, Domb BG (2016). Clinical outcomes of hip arthroscopy in radiographically diagnosed retroverted acetabula. Am J Sports Med..

[82] Hartigan DE, Perets I, Yuen LC, Domb BG (2017). Results of hip arthroscopy in patients with MRI diagnosis of subchondral cysts - a case series. J Hip Preserv Surg..

[83] Hevesi M, Hartigan DE, Wu IT, Levy BA, Domb BG, Krych AJ (2018). Are results of arthroscopic labral repair durable in dysplasia at midterm follow-up? A 2-center matched cohort analysis. Am J Sports Med..

[84] Hevesi M, Krych AJ, Johnson NR, Redmond JM, Hartigan DE, Levy BA (2018). Multicenter analysis of midterm clinical outcomes of arthroscopic labral repair in the hip: minimum 5-year follow-up. Am J Sports Med..

[85] Ibrahim MM, Poitras S, Bunting AC, Sandoval E, Beaulé PE (2018). Does acetabular coverage influence the clinical outcome of arthroscopically treated cam-type femoroacetabular impingement (FAI)?. Bone Joint J..

[86] Jackson TJ, Hammarstedt JE, Vemula SP, Domb BG (2015). Acetabular labral base repair versus circumferential suture repair: a matched-paired comparison of clinical outcomes. Arthroscopy..

[87] Jackson TJ, Lindner D, El-Bitar YF, Domb BG (2015). Effect of femoral anteversion on clinical outcomes after hip arthroscopy. Arthroscopy..

[88] Kang C, Hwang DS, Cha SM (2009). Acetabular labral tears in patients with sports injury. Clin Orthop Surg..

[89] Kemp JL, Makdissi M, Schache AG, Pritchard MG, Pollard TCB, Crossley KM (2014). Hip chondropathy at arthroscopy: prevalence and relationship to labral pathology, femoroacetabular impingement and patient-reported outcomes. Br J Sports Med..

[90] Klingenstein GG, Martin R, Kivlan B, Kelly BT (2012). Hip injuries in the overhead athlete. Clin Orthop Relat Res..

[91] Krych AJ, Thompson M, Knutson Z, Scoon J, Coleman SH (2013). Arthroscopic labral repair versus selective labral debridement in female patients with femoroacetabular impingement: a prospective randomized study. Arthroscopy..

[92] Kuhns BD, Hannon CP, Makhni EC, Alter J, Mather RC, Salata MJ (2017). A comparison of clinical outcomes after unilateral or bilateral hip arthroscopic surgery: age- and sex-matched cohort study. Am J Sports Med..

[93] Lansdown DA, Kunze K, Ukwuani G, Waterman BR, Nho SJ (2018). The importance of comprehensive cam correction: radiographic parameters are predictive of patient-reported outcome measures at 2 years after hip arthroscopy. Am J Sports Med..

[94] Levy DM, Cvetanovich GL, Kuhns BD, Greenberg MJ, Alter JM, Nho SJ (2017). Hip arthroscopy for atypical posterior hip pain: a comparative matched-pair analysis. Am J Sports Med..

[95] Levy DM, Kuhns BD, Frank RM, Grzybowski JS, Campbell KA, Brown S (2017). High rate of return to running for athletes after hip arthroscopy for the treatment of femoroacetabular impingement and capsular plication. Am J Sports Med..

[96] Locks R, Bolia IK, Utsunomiya H, Briggs KK, Philippon MJ (2018). Revision hip arthroscopy after labral reconstruction using iliotibial band autograft: surgical findings and comparison of outcomes with labral reconstructions not requiring revision. Arthroscopy..

[97] Lodhia P, Gui C, Chandrasekaran S, Suarez-Ahedo C, Vemula SP, Domb BG (2015). Microfracture in the hip: a matched-control study with average 3-year follow-up. J Hip Preserv Surg..

[98] Lodhia P, Gui C, Martin TJ, Chandrasekaran S, Suárez-Ahedo C, Walsh JP (2016). Arthroscopic central acetabular decompression: clinical outcomes at minimum 2-year follow-up using a matched-pair analysis. Arthroscopy..

[99] Lund B, Mygind-Klavsen B, Gronbech Nielsen T, Maagaard N, Kraemer O, Holmich P (2017). Danish Hip Arthroscopy Registry (DHAR): the outcome of patients with femoroacetabular impingement (FAI). J Hip Preserv Surg..

[100] Mansell NS, Rhon DI, Meyer J, Slevin JM, Marchant BG (2018). Arthroscopic surgery or physical therapy for patients with femoroacetabular impingement syndrome: a randomized controlled trial with 2-year follow-up. Am J Sports Med..

[101] Más Martínez J, Morales-Santías M (2014). Bustamante Suarez Suarez de Puga D, Sanz-Reig J. Hip arthroscopy in males younger than 40 with femoroacetabular impingement: short-term outcomes. Revista Espanola de Cirugia Ortopedica y Traumatologia..

[102] Michal F, Amar E, Atzmon R, Sharfman Z, Haviv B, Eisenberg G, et al. Subspinal impingement: clinical outcomes of arthroscopic decompression with one year minimum follow up. Knee Surg Sports Traumatol Arthrosc. 2018. 10.1007/s00167-018-4923-5. 10.1007/s00167-018-4923-5 29610973

[103] Nawabi DH, Degen RM, Fields KG, McLawhorn A, Ranawat AS, Sink EL (2016). Outcomes after arthroscopic treatment of femoroacetabular impingement for patients with borderline hip dysplasia. Am J Sports Med..

[104] Newman JT, Briggs KK, McNamara SC, Philippon MJ (2016). Revision hip arthroscopy: a matched-cohort study comparing revision to primary arthroscopy patients. Am J Sports Med..

[105] Nwachukwu BU, Chang B, Fields K, Rebolledo BJ, Nawabi DH, Kelly BT (2017). Defining the “substantial clinical benefit” after arthroscopic treatment of femoroacetabular impingement. Am J Sports Med..

[106] Ortiz-Declet V, Yuen LC, Maldonado DR, Schwarzman G, Laseter JR, Domb BG (2018). Return to play among golfers undergoing hip arthroscopy: short-to mid-term follow-up. Orthopedics..

[107] Perets I, Craig MJ, Mu BH, Maldonado DR, Litrenta JM, Domb BG. Midterm outcomes and return to sports among athletes undergoing hip arthroscopy. Am J Sports Med. 2018;46(7):1661–7. 10.1177/0363546518765969 29726692

[108] Perets I, Hartigan DE, Chaharbakhshi EO, Ashberg L, Mu B, Domb BG (2018). Clinical outcomes and return to sport in competitive athletes undergoing arthroscopic iliopsoas fractional lengthening compared with a matched control group without iliopsoas fractional lengthening. Arthroscopy..

[109] Perets I, Hartigan DE, Chaharbakhshi EO, Ashberg L, Ortiz-Declet V, Domb BG (2017). Outcomes of hip arthroscopy in competitive athletes. Arthroscopy..

[110] Perets I, Hartigan DE, Walsh JP, Chen AW, Mu BH, Domb BG (2018). Excision of labral amorphous calcification as a part of hip arthroscopy-clinical outcomes in a matched-controlled study. Arthroscopy..

[111] Perets I, Rybalko D, Chaharbakhshi EO, Mu BH, Chen AW, Domb BG (2018). Minimum five-year outcomes of hip arthroscopy for the treatment of femoroacetabular impingement and labral tears in patients with obesity: a match-controlled study. J Bone Joint Surg..

[112] Pergaminelis N, Renouf J, Fary C, Tirosh O, Tran P. Outcomes of arthroscopic debridement of isolated ligamentum teres tears using the iHOT-33. BMC Musculoskelet Disord. 2017;18. 10.1186/s12891-017-1905-6. 10.1186/s12891-017-1905-6 PMC5747181 29284482

[113] Redmond JM, El Bitar YF, Gupta A, Stake CE, Vemula SP, Domb BG (2015). Arthroscopic acetabuloplasty and labral refixation without labral detachment.[Erratum appears in Am J Sports Med. 2015;43(2):NP1 Note: Vemula, S Pavan [added]]. Am J Sports Med..

[114] Redmond JM, Gupta A, Stake CE, Hammarstedt JE, Finch NA, Domb BG (2015). Clinical results of hip arthroscopy for labral tears: a comparison between intraoperative platelet-rich plasma and bupivacaine injection. Arthroscopy..

[115] Rhee C, Amar E, Glazebrook M, Coday C, Wong IH. Safety profile and short-term outcomes of bst-cargel as an adjunct to microfracture for the treatment of chondral lesions of the hip. Orthop J Sports Med. 2018;6(8). 10.1177/2325967118789871 10.1177/2325967118789871 PMC6088484 30116764

[116] Riff AJ, Ukwuani G, Clapp I, Movassaghi K, Kelly DM, Nho SJ (2018). High rate of return to high-intensity interval training after arthroscopic management of femoroacetabular impingement syndrome. Am J Sports Med..

[117] Saltzman BM, Kuhns BD, Basques B, Leroux T, Alter J, Mather RC (2017). The influence of body mass index on outcomes after hip arthroscopic surgery with capsular plication for the treatment of femoroacetabular impingement. Am J Sports Med..

[118] Sansone M, Ahlden M, Jonasson P, Thomee C, Sward L, Baranto A, et al. Good results after hip arthroscopy for femoroacetabular impingement in top-level athletes. Orthop J Sports Med. 2015;3(2). 10.1177/2325967115569691 10.1177/2325967115569691 PMC4555608 26535379

[119] Sawyer GA, Briggs KK, Dornan GJ, Ommen ND, Philippon MJ (2015). Clinical outcomes after arthroscopic hip labral repair using looped versus pierced suture techniques. Am J Sports Med..

[120] Shaw KA, Jacobs JM, Evanson JR, Pniewski J, Dickston ML, Mueller T (2017). Functional outcomes of hip arthroscopy in an active duty military population utilizing a criterion-based early weight bearing progression. Int J Sports Phys Ther..

[121] Stake CE, Jackson TJ, Stone JC, Domb BG (2013). Hip arthroscopy for labral tears in workers’ compensation: a matched-pair controlled study. Am J Sports Med..

[122] Suarez-Ahedo C, Gui C, Rabe SM, Walsh JP, Chandrasekaran S, Domb BG. Relationship between age at onset of symptoms and intraoperative findings in hip arthroscopic surgery. Orthop J Sports Med. 2017;5(11). 10.1177/2325967117737480 10.1177/2325967117737480 PMC5714088 29226162

[123] Tahoun MF, Tey M, Mas J, Eid TA-E, Monllau JC (2018). Arthroscopic repair of acetabular cartilage lesions by chitosan-based scaffold: clinical evaluation at minimum 2 years follow-up. Arthroscopy..

[124] Tijssen M, van Cingel R, de Visser E, Nijhuis-van der Sanden M. A clinical observational study on patient-reported outcomes, hip functional performance and return to sports activities in hip arthroscopy patients. Phys Ther Sport. 2016;20:45-55. 10.1016/j.ptsp.2015.12.004 27325539

[125] Vap AR, Mitchell JJ, Briggs KK, McNamara SC, Philippon MJ (2018). Outcomes of arthroscopic management of trochanteric bursitis in patients with femoroacetabular impingement: a comparison of two matched patient groups. Arthroscopy..

[126] Waterman BR, Ukwuani G, Clapp I, Malloy P, Neal WH, Nho SJ (2018). Return to golf after arthroscopic management of femoroacetabular impingement syndrome. Arthroscopy..

[127] Weber AE, Kuhns BD, Cvetanovich GL, Grzybowski JS, Salata MJ, Nho SJ (2017). Amateur and recreational athletes return to sport at a high rate following hip arthroscopy for femoroacetabular impingement. Arthroscopy..

[128] Wu Z, Chen S, Li Y, Li H, Chen J (2016). Effect of centre-edge angle on clinical and quality of life outcomes after arthroscopic acetabular labral debridement. Int Orthop..

[129] Yoo J-I, Lee T-H, Kim J-Y, Kim J-H, Ha Y-C (2018). Outcomes of hip arthroscopy in a military population are similar to those in the civilian population: matched paired analysis at 2 years. Arthroscopy..

[130] Zimmerer A, Bock M, Hoffmann M, Miehlke W, Sobau C (2017). Return to work after arthroscopic surgery for femoroacetabular impingement in patients younger than 30 years. Sports Orthopaedics Traumatology..

[131] Bennell KL, Spiers L, Takla A, O’Donnell J, Kasza J, Hunter DJ, et al. Efficacy of adding a physiotherapy rehabilitation programme to arthroscopic management of femoroacetabular impingement syndrome: a randomised controlled trial (FAIR). BMJ Open. 2017;7(6): https://doi.org/ 10.1136/ bmjopen-2016-014658 10.1136/bmjopen-2016-014658 PMC5623417 28645960

[132] Philippon M, Briggs K, Yen Y-M, Kuppersmith D (2009). Outcomes following hip arthroscopy for femoroacetabular impingement with associated chondrolabral dysfunction: minimum two-year follow-up. J Bone Joint Surg Br..

[133] Basques BA, Waterman BR, Ukwuani G, Beck EC, Neal WH, Friel NA (2019). Preoperative symptom duration is associated with outcomes after hip arthroscopy. Am J Sports Med.

[134] Beck EC, Kunze KN, Friel NA, Neal WH, Fu MC, Giordano BD (2019). Is there a correlation between outcomes after hip arthroscopy for femoroacetabular impingement syndrome and patient cortical bone thickness?. J Hip Preserv Surg..

[135] Beck EC, Nwachukwu BU, Chahla J, Jan K, Keating TC, Suppauksorn S (2019). Patients with borderline hip dysplasia achieve clinically significant outcome after arthroscopic femoroacetabular impingement surgery: a case-control study with minimum 2-year follow-up. Am J Sports Med..

[136] Bolia IK, Fagotti L, Briggs KK, Philippon MJ (2019). Midterm outcomes following repair of capsulotomy versus nonrepair in patients undergoing hip arthroscopy for femoroacetabular impingement with labral repair. Arthroscopy..

[137] Cancienne J, Kunze KN, Beck EC, Chahla J, Suppauksorn S, Nho SJ (2019). Influence of cigarette smoking at the time of surgery on postoperative outcomes in patients with femoroacetabular impingement: a matched-pair cohort analysis. Am J Sports Med..

[138] Chaharbakhshi EO, Hartigan DE, Perets I, Domb BG (2019). Is hip arthroscopy effective in patients with combined excessive femoral anteversion and borderline dysplasia? A match-controlled study. Am J Sports Med..

[139] Chahla J, Beck EC, Nwachukwu BU, Alter T, Harris JD, Nho SJ. Is there an association between preoperative expectations and patient-reported outcome after hip arthroscopy for femoroacetabular impingement syndrome? Arthroscopy. 2019;35(12):3250-8.e1. 10.1016/j.arthro.2019.06.018 31785753

[140] Chahla J, Nwachukwu BU, Beck EC, Neal WH, Cancienne J, Okoroha KR (2019). Influence of acetabular labral tear length on outcomes after hip arthroscopy for femoroacetabular impingement syndrome with capsular plication. Am J Sports Med..

[141] Chambers CC, Monroe EJ, Flores SE, Borak KR, Zhang AL (2019). Periportal capsulotomy: technique and outcomes for a limited capsulotomy during hip arthroscopy. Arthroscopy..

[142] Chandrasekaran S, Darwish N, Darwish AH, Suarez-Ahedo C, Lodhia P, Domb BG (2019). Outcomes of hip arthroscopy in patients with previous lumbar spine surgery: a matched-pair controlled comparative study with minimum two-year follow-up. Arthroscopy..

[143] Clapp IM, Nwachukwu BU, Beck EC, Jan K, Gowd AK, Nho SJ. Comparing outcomes of competitive athletes versus nonathletes undergoing hip arthroscopy for treatment of femoroacetabular impingement syndrome. Am J Sports Med. 2019. 10.1177/0363546519885359. 10.1177/0363546519885359 31743036

[144] Frank RM, Kunze KN, Beck EC, Neal WH, Bush-Joseph CA, Nho SJ (2019). Do female athletes return to sports after hip preservation surgery for femoroacetabular impingement syndrome? A comparative analysis. Orthop. J. Sports Med..

[145] Hassebrock JD, Krych AJ, Domb BG, Levy BA, Neville MR, Hartigan DE (2019). Bilateral hip arthroscopy: can results from initial arthroscopy for femoroacetabular impingement predict future contralateral results?. Arthroscopy..

[146] Hevesi M, Bernard C, Hartigan DE, Levy BA, Domb BG, Krych AJ (2019). Is microfracture necessary? Acetabular chondrolabral debridement/abrasion demonstrates similar outcomes and survival to microfracture in hip arthroscopy: a multicenter analysis. Am J Sports Med..

[147] Krishnamoorthy VP, Kunze KN, Beck EC, Cancienne JM, O'Keefe LS, Ayeni OR (2019). Radiographic prevalence of symphysis pubis abnormalities and clinical outcomes in patients with femoroacetabular impingement syndrome. Am J Sports Med..

[148] Kunze KN, Beck EC, Nwachukwu BU, Ahn J, Nho SJ (2019). Early hip arthroscopy for femoroacetabular impingement syndrome provides superior outcomes when compared with delaying surgical treatment beyond 6 months. Am J Sports Med..

[149] Lee JW, Hwang DS, Kang C, Hwang JM, Chung HJ (2019). Arthroscopic repair of acetabular labral tears associated with femoroacetabular impingement: 7-10 years of long-term follow-up results. Clin Orthop Surg..

[150] Ohlin A, Ahlden M, Lindman I, Jonasson P, Desai N, Baranto A, et al. Good 5-year outcomes after arthroscopic treatment for femoroacetabular impingement syndrome. Knee Surg Sports Traumatol Arthrosc. 2019. 10.1007/s00167-019-05429-y. 10.1007/s00167-019-05429-y PMC7148278 30972465

[151] Stone AV, Beck EC, Malloy P, Chahla J, Nwachukwu BU, Neal WH, et al. Preoperative predictors of achieving clinically significant athletic functional status after hip arthroscopy for femoroacetabular impingement at minimum 2-year follow-up. Arthroscopy. 2019;35(11):3049-56.e1. 10.1016/j.arthro.2019.05.022 31395395

[152] Stone AV, Mehta N, Beck EC, Waterman BR, Chahla J, Ukwuani G (2019). Comparable patient-reported outcomes in females with or without joint hypermobility after hip arthroscopy and capsular plication for femoroacetabular impingement syndrome. J Hip Preserv Surg..

[153] Ukwuani GC, Waterman BR, Nwachukwu BU, Beck EC, Kunze KN, Harris JD, et al. Return to dance and predictors of outcome after hip arthroscopy for femoroacetabular impingement syndrome. Arthroscopy. 2019;35(4):1101-8.e3. 10.1016/j.arthro.2018.10.121 30857899

[154] Kierkegaard S, Dalgas U, Lund B, Lipperts M, Søballe K, Mechlenburg I (2019). Despite patient-reported outcomes improve, patients with femoroacetabular impingement syndrome do not increase their objectively measured sport and physical activity level 1 year after hip arthroscopic surgery.

[155] Landis J, Koch G (1977). Agreement measures for categorical data. Biometrics..

[156] Thompson WR (2017). Worldwide survey of fitness trends for 2018: the CREP edition. ACSM's Health & Fitness Journal..

[157] Bassett DR, Freedson PS, John D (2019). Wearable activity trackers in clinical research and practice. Kinesiol Rev..

[158] Renouf J, Pergaminelis N, Tran P, Tirosh O, Fary C (2019). Prevalence and trends of patient-reported outcome measures used in hip arthroscopy. Orthopedics..

[159] Szucs D, Ioannidis JP. Empirical assessment of published effect sizes and power in the recent cognitive neuroscience and psychology literature. PLoS Biol. 2017;15(3).DOI 10.1371/journal.pbio.2000797 10.1371/journal.pbio.2000797 PMC5333800 28253258

[160] Sansone M, Ahlden M, Jonasson P, Thomee C, Sward L, Baranto A (2014). A Swedish hip arthroscopy registry: demographics and development. Knee Surg Sports Traumatol Arthrosc..

