# Supplementary material for: Physical Activity Following Hip Arthroscopy in Young and Middle-Aged Adults: A Systematic Review
Source: Sports Med Open. 2020 Jan 28;6:7. doi: 10.1186/s40798-020-0234-8 (PMC6987281; doi:10.1186/s40798-020-0234-8)
Supplement: Supplementary file 1 — Additional file 1: Example search strategy. [file 40798_2020_234_MOESM1_ESM.pdf]

Additional File 1. Example search strategy.

Database: Ovid MEDLINE(R) Epub Ahead of Print, In-Process & Other Non-Indexed Citations, Ovid MEDLINE(R) Daily and Ovid MEDLINE(R) <1946 to Present>

Search Strategy:

----

1 Arthroscopy/ (22499)

2 arthroscop*.mp. [mp=title, abstract, original title, name of substance word, subject heading word, keyword heading word, protocol supplementary concept word, rare disease supplementary concept word, unique identifier, synonyms] (32973)

3 Hip/ (11702)

4 Hip Joint/ (26682)

5 hip.mp. [mp=title, abstract, original title, name of substance word, subject heading word, keyword heading word, protocol supplementary concept word, rare disease supplementary concept word, unique identifier, synonyms] (147662)

6 hip joint.mp. [mp=title, abstract, original title, name of substance word, subject heading word, keyword heading word, protocol supplementary concept word, rare disease supplementary concept word, unique identifier, synonyms] (33071)

7 Exercise/ (93177)

8 physical activit*.mp. [mp=title, abstract, original title, name of substance word, subject heading word, keyword heading word, protocol supplementary concept word, rare disease supplementary concept word, unique identifier, synonyms] (94198)

9 exercis*.mp. [mp=title, abstract, original title, name of substance word, subject heading word, keyword heading word, protocol supplementary concept word, rare disease supplementary concept word, unique identifier, synonyms] (345891)

10 sport*.mp. [mp=title, abstract, original title, name of substance word, subject heading word, keyword heading word, protocol supplementary concept word, rare disease supplementary concept word, unique identifier, synonyms] (91469)

11 Sports/ (29427)

12 athlet*.mp. [mp=title, abstract, original title, name of substance word, subject heading word, keyword heading word, protocol supplementary concept word, rare disease supplementary concept word, unique identifier, synonyms] (77683)

13 Adult/ or Young Adult/ (4897986)

14 adult*.mp. [mp=title, abstract, original title, name of substance word, subject heading word, keyword heading word, protocol supplementary concept word, rare disease supplementary concept word, unique identifier, synonyms] (5570417)

15 13 or 14 (5570417)

16 7 or 8 or 9 or 10 or 11 or 12 (493029)

17 1 or 2 (32973)

18 3 or 4 or 5 or 6 (147662)

19 17 and 18 (2755)

20 15 and 16 and 19 (247)

21 "hip arthroscop*".mp. [mp=title, abstract, original title, name of substance word, subject heading word, keyword heading word, protocol supplementary concept word, rare disease supplementary concept word, unique identifier, synonyms] (1481)

22 (hip adj5 arthroscop*).mp. [mp=title, abstract, original title, name of substance word, subject heading word, keyword heading word, protocol supplementary concept word, rare disease supplementary concept word, unique identifier, synonyms] (1823)

23 19 or 21 or 22 (2755)

24 15 and 16 and 23 (247)

25 15 and 23 (1202)
