# Supplementary material for: Physical Activity Following Hip Arthroscopy in Young and Middle-Aged Adults: A Systematic Review
Source: Sports Med Open. 2020 Jan 28;6:7. doi: 10.1186/s40798-020-0234-8 (PMC6987281; doi:10.1186/s40798-020-0234-8)
Supplement: Supplementary file 2 — Additional file 2: Study quality assessment forms. [file 40798_2020_234_MOESM2_ESM.pdf]

Additional File 2. Study quality assessment

A- Single arm study

			Question	Guidance	Response [Delete as appropriate]	Notes
External Validity						
	1	Representative	Is the sample representative of the population from which they were recruited?	To facilitate this, a study needs to identify the source of the population and describe how the participants are selected (explicitly defined inclusion / exclusion criteria).	✓ the sample is representative of the population from which they were recruited.<br>X not representative or insufficient information for this judgement to be made.	
	2	Participation rate	Did the majority of 'recruited and eligible' participants take part in the data collection?	Calculated as the number of potential participants who are eligible to take part in the study that are then included in the initial sport/activity analysis.<br>i.e. Loss of participants between establishing eligibility (having been recruited) and data collection	✓ percent participation was 80% or more<br>X less than 80% recruited and eligible start data collection	
Internal Validity						
Performance Bias	3	Direct observation	Were the data collected directly from the subjects?	As opposed to by proxy - e.g. sport participation data may be reported by coach or from match statistics.	✓ if **all** sports/activity outcomes reported are collected directly from subjects<br>X all data not collected directly from subjects	
Detection Bias	4	PROM – validity / reliability	Are the data collection tools used established as valid and reliable for the population being assessed?	The PROM used has been reported in the paper as having adequate reliability and validity, e.g. test-retest, piloting, validation in a previous study, with references included.	✓ if a this is established **and** reported<br>X not established and reported<br>NA no PROM's used	
	5	Direct observation, (objective measures)	Are the data collection tools used established as valid and reliable for the population being assessed?	The data collection tool used has been reported in the paper as having adequate reliability and validity, e.g. test-retest, piloting, validation in a previous study, with references included.	✓ if a this is established and reported.<br>X not established and reported<br>NA no direct measure of PA used that is not a PROM	
	6	Blinded assessors	Were those responsible for assessing data blinded?		✓ indicates that assessors were blinded.<br>X if no or unable to establish.	
	7	Outcome measure	Was the same outcome measure used for all participants?		✓ indicates same method of ascertainment was used for all participants<br>X same method of ascertainment was not used for all participants	
Attrition	8	Completeness	Do all the participants entering data collection reach the results section?	There is clear accounting for participant numbers within the results.	✓ percentage of participants in the final analysis was 80 or more, or a full description of those lost to follow-up was not suggestive of bias.<br>X <80% in final analysis, unaccounted for or suggestive of bias	

			Question	Guidance	Response [Delete as appropriate]	Notes
Selection bias/control of confounding	9	Age	Are all participants within the age **range** of 18-50?		✓ if **range** within 18-50<br>X if range is outside 18-50 and not adjusted for in analysis or insufficient information for judgement to be made.	
	10	Location	Are participants from a comparable location?	It is identified that all participants are from a location that offers comparable facilities for care	✓ if the location of participants is comparable.<br>X not comparable or unknown	
	11	Gender	Is there a balance of genders in the included cases?	Analysis identifies a balance of men and women participants or is adjusted for in analysis / reporting	✓ if gender is balanced (10% or less difference) or adjusted for in analysis<br>X ->10% difference not adjusted for in analysis or insufficient information for this judgement to be made.	
	12	Severity of joint disease - OA	Is OA identified in the study?	Degree of OA has been screened for, however, some studies did not have sufficient information to enable a decision to be made, these need to be identified.	✓ if severity of OA identified in the study<br>X insufficient information for this judgement to be made/OA severity not reported.	
	13	Follow-up	Are the time points at which the outcome is measured the same for all participants?	Assessment of point at which data is collected in relation to surgery or<br>other pre-defined time point such as symptom onset /unable to participate in sport, if this is the primary end point instead of surgery	✓ where FU is the same for all study participants or lies within 10% i.e. the following acceptable ranges – 1 year follow-up, 1 month each way; 2 years follow-up = 2 months; 3 years follow-up = 3months……10 years = 10 months.<br>X where FU differs by >10% or insufficient information for this judgement to be made	

B- Multiple arm study

			Question	Guidance	Response [delete as appropriate]	Notes
External Validity						
	1	Representative	Is the sample representative of the population from which they were recruited?	To facilitate this, a study needs to identify the source of the population and describe how the participants are selected (explicitly defined inclusion / exclusion criteria).	✓ the sample is representative of the population from which they were recruited.<br>X not representative or insufficient information for this judgement to be made.	
	2	Participation rate	Did the majority of 'recruited and eligible' participants take part in the data collection?	Calculated as the number of potential participants who are eligible to take part in the study that are then included in the initial sport/activity analysis.<br>i.e. Loss of participants between establishing eligibility (having been recruited) and data collection unless accounted for (e.g. loss as part of matching process)	✓ percent participation was 80% or more<br>X less than 80% recruited and eligible start data collection	
Internal Validity						

			Question	Guidance	Response [delete as appropriate]	Notes
Performance Bias	3	Direct observation	Were the data collected directly from the subjects?	As opposed to by proxy - e.g. sport participation data may be reported by coach or from match statistics.	✓ if **all** sports/activity outcomes reported are collected directly from subjects<br>X – all data not collected directly from subjects	
Detection Bias	4	PROM – validity / reliability	Are the data collection tools used established as valid and reliable for the population being assessed?	The PROM used has been reported in the paper as having adequate reliability and validity, e.g. test-retest, piloting, validation in a previous study, with references included.	✓ if a this is established **and** reported<br>X not established and reported<br>NA no PROM's used	
	5	Direct observation, (objective measures)	Are the data collection tools used established as valid and reliable for the population being assessed?	The data collection tool used has been reported in the paper as having adequate reliability and validity, e.g. test-retest, piloting, validation in a previous study, with references included.	✓ if a this is established and reported.<br>X not established and reported<br>NA – no direct measure of PA used that is not a PROM	
	6	Blinded assessors	Were those responsible for assessing data blinded?		✓ indicates that assessors were blinded.<br>X if no, or unable to establish.	
	7	Outcome measure	Was the same outcome measure used for all participants?		✓ indicates same method of ascertainment was used for all participants<br>X same method of ascertainment was not used for all participants	
Attrition	8	Completeness	Do all the participants entering data collection reach the results section?	There is clear accounting for participant numbers within the results.	✓ percentage of participants in the final analysis was 80 or more, or a full description of those lost to follow-up was not suggestive of bias.<br>X <80% in final analysis, unaccounted for or suggestive of bias	
Selection bias/control of confounding	9	Age	Is there a significant difference in the age profile of the compared groups?<br>Are all participants within the age **range** of 18-50?	Analysis identifies that the comparison groups are not statistically different. Sufficient data needs to be available to enable this analysis if not undertaken by authors.	✓ if age is balanced between groups (10% or less difference) or adjusted for in analysis and range lies between 18-50.<br>X if >10% difference or range is outside 18-50 or insufficient information for judgement to be made.	
	10	Location	Are compared groups from comparable location?	It is identified that compared groups are from a location that offers comparable facilities for care.	✓ if the location of comparison groups is comparable.<br>X not comparable or unknown	
	11	Gender	Is there a significant difference in the number of men and women in the compared groups?	Analysis identifies that the comparison groups are not statistically different. Sufficient data needs to be available to enable this analysis if not directly reported	✓ if gender is balanced between groups (10% or less difference) or adjusted for in analysis.<br>X if >10% or insufficient information for this judgement to be made.	
	12	Severity of joint disease - OA	Is OA identified in the study?	Degree of OA has been screened for, however, some studies did not have sufficient information to enable a decision to be made, these need to be identified.	✓ if severity of OA identified in the study<br>X insufficient information for this judgement to be made/OA severity not reported.	

			Question	Guidance	Response [delete as appropriate]	Notes
	13	Follow-up	Are the time points at which the outcome is measured the same for both groups?	Assessment of point at which data is collected in relation to surgery or<br>other pre-defined time point such as symptom onset /unable to participate in sport, if this is the primary end point instead of surgery.	✓ where FU is the same for all study participants or lies within 10% i.e. the following acceptable ranges – 1 year follow-up, 1 month each way; 2 years follow-up = 2 months; 3 years follow-up = 3months……10 years = 10 months.<br>X where FU differs by >10% or insufficient information for this judgement to be made.	
