# Supplementary material for: Physical Activity Following Hip Arthroscopy in Young and Middle-Aged Adults: A Systematic Review
Source: Sports Med Open. 2020 Jan 28;6:7. doi: 10.1186/s40798-020-0234-8 (PMC6987281; doi:10.1186/s40798-020-0234-8)
Supplement: Supplementary file 3 — Additional file 3: Studies excluded at full text screen. [file 40798_2020_234_MOESM3_ESM.pdf]

Additional file 3: Studies excluded at full text screen

Author	Year	Title
**Greater than 10% of cohort with OA**		
Awan, N.;Murray, P.;	2006	Role of hip arthroscopy in the diagnosis and treatment of hip joint pathology
Brunner, Alexander;Horisberger, Monika;Herzog, Richard F.;	2009	Sports and Recreation Activity of Patients With Femoroacetabular Impingement Before and After Arthroscopic Osteoplasty
Byrd, J. W.;Jones, K. S.;	2009	Hip arthroscopy in athletes: 10-year follow-up
Geyer, Mark R.;Philippon, Marc J.;Fagrelius, Theodore S.;Briggs, Karen K.;	2013	Acetabular Labral Reconstruction With an Iliotibial Band Autograft: Outcome and Survivorship Analysis at Minimum 3-Year Follow-up
Menge, Travis J.;Briggs, Karen K.;Dornan, Grant J.;McNamara, Shannen C.;Philippon, Marc J.;	2017	Survivorship and Outcomes 10 Years Following Hip Arthroscopy for Femoroacetabular Impingement: Labral Debridement Compared with Labral Repair
Murata, Y.;Uchida, S.;Utsunomiya, H.;Hatakeyama, A.;Nakamura, E.;Sakai, A.;	2017	A Comparison of Clinical Outcome between Athletes and Nonathletes Undergoing Hip Arthroscopy for Femoroacetabular Impingement
Sansone, M.;Ahlden, M.;Jonasson, P.;Thomee, C.;Sward, L.;Collin, D.;Baranto, A.;Karlsson, J.;Thomee, R.;	2016	Outcome of hip arthroscopy in patients with mild to moderate osteoarthritis-A prospective study
Skendzel, Jack G.;Philippon, Marc J.;Briggs, Karen K.;Goljan, Peter;	2014	The Effect of Joint Space on Midterm Outcomes After Arthroscopic Hip Surgery for Femoroacetabular Impingement
Tjong, V. K.;Gombera, M. M.;Kahlenberg, C. A.;Patel, R. M.;Han, B.;Deshmane, P.;Terry, M. A.;	2017	Isolated Acetabuloplasty and Labral Repair for Combined-Type Femoroacetabular Impingement: Are We Doing Too Much?
**No suitable physical activity outcome available**		
Becker, Lindsay C.;Carter-Kelley, Stephanie;Ellis, Thomas;Cenkus, Kathleen;Di Stasi, Stephanie L.;	2015	Pre-operative low back pain negatively affects self-reported function in individuals undergoing hip arthroscopy
Bretschneider, H.;Trattnig, S.;Landgraeber, S.;Hartmann, A.;Gunther, K. P.;Dienst, M.;Schroder, J.;Fickert, S.;	2019	Arthroscopic matrix-associated, injectable autologous chondrocyte transplantation of the hip: significant improvement in patient-related outcome and good transplant quality in MRI assessment
Byrd, J. W.;Jones, K. S.;Chin, P. C.;	2016	Hip arthroscopy: a report on a cohort of orthopaedic surgeons
Cetinkaya, S.;Toker, B.;Ozden, V. E.;Dikmen, G.;Taser, O.;	2016	Arthroscopic labral repair versus labral debridement in patients with femoroacetabular impingement: a minimum 2.5 year follow-up study
Domb, B. G.;Gupta, A.;Dunne, K. F.;Gui, C.;Chandrasekaran, S.;Lodhia, P.;	2015	Microfracture in the Hip: Results of a Matched-Cohort Controlled Study with 2-Year Follow-up
Farjo, L. A.;Glick, J. M.;Sampson, T. G.;	1999	Hip arthroscopy for acetabular labral tears
Gigi, R.;Rath, E.;Sharfman, Z. T.;Shimonovich, S.;Ronen, I.;Amar, E.;	2016	Hip Arthroscopy for Femoral-Acetabular Impingement: Do Active Claims Affect Outcomes?
Grammatopoulos, George;Davies, Owain L. I.;El-Bakoury, Ahmed;Gill, Harinderjit S.;Pollard, Tom C. B.;Andrade, Antonio J.;	2017	A Traffic Light Grading System of Hip Dysplasia to Predict the Success of Arthroscopic Hip Surgery
Hartig-Andreasen, C.;Nielsen, T. G.;Lund, B.;Soballe, K.;Lind, M.;	2017	Outcome after arthroscopic labral surgery in patients previously treated with periacetabular osteotomy: a follow-up study of 43 patients
Joseph, Roody;Pan, Xueliang;Cenkus, Kathleen;Brown, Lindsey;Ellis, Thomas;Di Stasi, Stephanie;	2016	Sex Differences in Self-Reported Hip Function Up to 2 Years After Arthroscopic Surgery for Femoroacetabular Impingement

Kalisvaart, M. M.;Safran, M. R.;	2017	Hip instability treated with arthroscopic capsular plication
Kamath, A. F.;Componovo, R.;Baldwin, K.;Israelite, C. L.;Nelson, C. L.;	2009	Hip arthroscopy for labral tears: Review of clinical outcomes with 4.8-year Mean follow-up
Kemp, Joanne;Makdissi, Michael;Schache, Anthony;Finch, Caroline;Pritchard, Michael;Crossley, Kay;Kemp, Joanne L.;Schache, Anthony G.;Finch, Caroline F.;Pritchard, Michael G.;Crossley, Kay M.;	2016	Is quality of life following hip arthroscopy in patients with chondrolabral pathology associated with impairments in hip strength or range of motion?
Knapik, D. M.;Sheehan, J.;Nho, S. J.;Voos, J. E.;Salata, M. J.;	2018	Prevalence and Impact of Hip Arthroscopic Surgery on Future Participation in Elite American Football Athletes
Lee, Simon;Frank, Rachel M.;Harris, Joshua;Song, Sang Hoon;Bush-Joseph, Charles A.;Salata, Michael J.;Nho, Shane J.;	2015	Evaluation of Sexual Function Before and After Hip Arthroscopic Surgery for Symptomatic Femoroacetabular Impingement
Matsuda, D. K.;Kivlan, B. R.;Nho, S. J.;Wolff, A. B.;Salvo, J. P., Jr.;Christoforetti, J. J.;Ellis, T. J.;Carreira, D. S.;	2019	Arthroscopic Outcomes as a Function of Acetabular Coverage From a Large Hip Arthroscopy Study Group
Matsuda, D. K.;Wolff, A. B.;Nho, S. J.;Salvo, J. P., Jr.;Christoforetti, J. J.;Kivlan, B. R.;Ellis, T. J.;Carreira, D. S.;	2018	Hip Dysplasia: Prevalence, Associated Findings, and Procedures From Large Multicenter Arthroscopy Study Group
McCarthy, J.;Barsoum, W.;Puri, L.;Lee, J. A.;Murphy, S.;Cooke, P.;	2003	The role of hip arthroscopy in the elite athlete
Mei-Dan, O.;McConkey, M. O.;Knudsen, J. S.;Brick, M. J.;	2014	Bilateral hip arthroscopy under the same anesthetic for patients with symptomatic bilateral femoroacetabular impingement: 1-year outcomes
Mullins, K.;Hanlon, M.;Carton, P.;	2019	Arthroscopic correction of femoroacetabular impingement improves athletic performance in male athletes
Nielsen, T. G.;Miller, L. L.;Lund, B.;Christiansen, S. E.;Lind, M.;	2014	Outcome of arthroscopic treatment for symptomatic femoroacetabular impingement
Pontiff, M.;Ithurburn, M. P.;Ellis, T.;Cenkus, K.;Stasi, S. D.;	2016	Pre- and post-operative self-reported function and quality of life in women with and without generalized joint laxity undergoing hip arthroscopy for femoroacetabular impingement
Renouf, J.;Pergaminelis, N.;Tran, P.;Fary, C.;Tirosh, O.;	2019	The outcome of arthroscopic repair of acetabular labral tears using the iHOT-33
Sanders, Thomas;Reardon, Patrick;Levy, Bruce;Krych, Aaron;Sanders, Thomas L.;Levy, Bruce A.;Krych, Aaron J.;	2017	Arthroscopic treatment of global pincer-type femoroacetabular impingement
Sochacki, K. R.;Jack, R. A., 2nd;Bekhradi, A.;Delgado, D.;McCulloch, P. C.;Harris, J. D.;	2018	Are Self-Reported Medication Allergies Associated With Worse Hip Outcome Scores Prior to Hip Arthroscopy?
Thier, S.;Baumann, F.;Weiss, C.;Fickert, S.;	2017	Feasibility of arthroscopic autologous chondrocyte implantation in the hip using an injectable hydrogel
Thier, S.;Weiss, C.;Fickert, S.;	2017	Arthroscopic autologous chondrocyte implantation in the hip for the treatment of full-thickness cartilage defects - A case series of 29 patients and review of the literature
Zingg, P. O.;Ulbrich, E. J.;Buehler, T. C.;Kalberer, F.;Poutawera, V. R.;Dora, C.;	2013	Surgical hip dislocation versus hip arthroscopy for femoroacetabular impingement: Clinical and morphological short-term results
**Non-English text**		
Bohnsack, M.;Lekkos, K.;Börner, C. E.;Wirth, C. J.;Rühmann, O.;	2006	Results of hip arthroscopy in sports related groin pain
Funakoshi, N.;Yamashita, F.;Nagaoka, T.;Mori, D.;	2011	Surgical Treatment of Acetabular Labral Tears in Athletes
**No comparative pre/post scores**		
Botser, I. B.;Jackson, T. J.;Smith, T. W.;Leonard, J. P.;Stake, C. E.;Domb, B. G.;	2014	Open surgical dislocation versus arthroscopic treatment of femoroacetabular impingement

Briggs, Karen K.;Soares, Eduardo;Bhatia, Sanjeev;Philippon, Marc J.;	2019	Postoperative alpha angle not associated with patient-centered midterm outcomes following hip arthroscopy for FAI
Briggs, Karen K;Soares, Eduardo;Bhatia, Sanjeev;Philippon, Marc J;	2018	Postoperative alpha angle not associated with patient-centered midterm outcomes following hip arthroscopy for FAI
Bryan, Andrew J.;Krych, Aaron J.;Pareek, Ayoosh;Reardon, Patrick J.;Berardelli, Rebecca;Levy, Bruce A.;	2016	Are Short-term Outcomes of Hip Arthroscopy in Patients 55 Years and Older Inferior to Those in Younger Patients?
Filbay, S. R.;Kemp, J. L.;Ackerman, I. N.;Crossley, K. M.;	2016	Quality of life impairments after hip arthroscopy in people with hip chondropathy
Giordano, B. D.;Suarez-Ahedo, C.;Gui, C.;Darwish, N.;Lodhia, P.;Domb, B. G.;	2018	Clinical outcomes of patients with symptomatic acetabular rim fractures after arthroscopic FAI treatment
Ishoi, L.;Thorborg, K.;Kraemer, O.;Lund, B.;Mygind-Klavsen, B.;Holmich, P.;	2019	Demographic and Radiographic Factors Associated With Intra-articular Hip Cartilage Injury: A Cross-sectional Study of 1511 Hip Arthroscopy Procedures
Kivlan, B. R.;Nho, S. J.;Christoforetti, J. J.;Ellis, T. J.;Matsuda, D. K.;Salvo, J. P., Jr.;Wolff, A. B.;Van Thiel, G. S.;Stubbs, A. J.;Carreira, D. S.;	2017	Multicenter Outcomes After Hip Arthroscopy: Epidemiology (MASH Study Group). What Are We Seeing in the Office, and Who Are We Choosing to Treat?
Krych, Aaron J.;King, Alexander H.;Berardelli, Rebecca L.;Sousa, Paul L.;Levy, Bruce A.;	2016	Is Subchondral Acetabular Edema or Cystic Change on MRI a Contraindication for Hip Arthroscopy in Patients With Femoroacetabular Impingement?
Krych, Aaron;Kuzma, Scott;Kovachevich, Rudy;Hudgens, Joshua;Stuart, Michael;Levy, Bruce;	2014	Modest mid-term outcomes after isolated arthroscopic debridement of acetabular labral tears
Maldonado, David R;Krych, Aaron J;Levy, Bruce A;Hartigan, David E;Laseter, Joseph R;Domb, Benjamin G;	2018	Does Iliopsoas Lengthening Adversely Affect Clinical Outcomes After Hip Arthroscopy? A Multicenter Comparative Study
Mygind-Klavsen, B.;Gronbech Nielsen, T.;Maagaard, N.;Kraemer, O.;Holmich, P.;Winge, S.;Lund, B.;Lind, M.;	2016	Danish Hip Arthroscopy Registry: an epidemiologic and perioperative description of the first 2000 procedures
Nawabi, D. H.;Degen, R. M.;Fields, K. G.;Wentzel, C. S.;Adeoye, O.;Kelly, B. T.;	2017	Anterior Inferior Iliac Spine Morphology and Outcomes of Hip Arthroscopy in Soccer Athletes: A Comparison to Nonkicking Athletes
Palmer, A. J. R.;Ayyar Gupta, V.;Fernquest, S.;Rombach, I.;Dutton, S. J.;Mansour, R.;Wood, S.;Khanduja, V.;Pollard, T. C. B.;McCaskie, A. W.;Barker, K. L.;Andrade, Tjmd;Carr, A. J.;Beard, D. J.;Glyn-Jones, S.;	2019	Arthroscopic hip surgery compared with physiotherapy and activity modification for the treatment of symptomatic femoroacetabular impingement: multicentre randomised controlled trial
Sansone, M.;Ahlden, M.;Jonasson, P.;Thomee, C.;Sward, L.;Baranto, A.;Karlsson, J.;Thomee, R.;	2014	A Swedish hip arthroscopy registry: demographics and development
Shibata, Kotaro R.;Matsuda, Shuichi;Safran, Marc R.;	2017	Arthroscopic Hip Surgery in the Elite Athlete: Comparison of Female and Male Competitive Athletes
Sochacki, Kyle R;Brown, Lindsey;Cenkus, Kathleen;Di Stasi, Stephanie;Harris, Joshua D;Ellis, Thomas J;	2018	Preoperative Depression Is Negatively Associated With Function and Predicts Poorer Outcomes After Hip Arthroscopy for Femoroacetabular Impingement
Spiker, A. M.;Rotter, B. Z.;Chang, B.;Mintz, D. N.;Kelly, B. T.;	2018	Clinical presentation of intra-articular osteoid osteoma of the hip and preliminary outcomes after arthroscopic resection: a case series
Tjong, V. K.;Cogan, C. J.;Riederman, B. D.;Terry, M. A.;	2016	A Qualitative Assessment of Return to Sport After Hip Arthroscopy for Femoroacetabular Impingement

Westermann, R. W.;Lynch, T. S.;Jones, M. H.;Spindler, K. P.;Messner, W.;Strnad, G.;Rosneck, J.;	2017	Predictors of Hip Pain and Function in Femoroacetabular Impingement: A Prospective Cohort Analysis
White, B. J.;Patterson, J.;Herzog, M. M.;	2018	Bilateral Hip Arthroscopy: Direct Comparison of Primary Acetabular Labral Repair and Primary Acetabular Labral Reconstruction
**Same cohort/subcohort and outcomes as another publication**		
Ashberg, Lyall;Close, Mary R.;Perets, Itay;Chaharbakhshi, Edwin O.;Walsh, John P.;Mohr, Mitchell R.;Domb, Benjamin G.;	2019	Do Femoral Head Osteochondral Lesions Predict a Poor Outcome in Hip Arthroscopy Patients? A Matched Control Study With Minimum 5-Year Follow-Up
Byrd, JW Thomas;Bardowski, Elizabeth A;Jones, Kay S;	2018	Influence of Tönnis Grade on Outcomes of Arthroscopic Management of Symptomatic Femoroacetabular Impingement
Chaharbakhshi, E. O.;Hartigan, D. E.;Spencer, J. D.;Perets, I.;Lall, A. C.;Domb, B. G.	2019	Do Larger Acetabular Chondral Defects Portend Inferior Outcomes in Patients Undergoing Arthroscopic Acetabular Microfracture? A Matched-Controlled Study
Chandrasekaran, S.;Darwish, N.;Mu, B. H.;Rybalko, D. A.;Perets, I.;Suarez-Ahedo, C.;Chaharbakhshi, E. O.;Lall, A. C.;Domb, B. G.;	2019	Arthroscopic Reconstruction of the Irreparable Acetabular Labrum: A Match-controlled Study
Chen, A. W.;Craig, M. J.;Mu, B. H.;Go, C. C.;Ortiz-Declet, V.;Maldonado, D. R.;Domb, B. G.;	2019	Return to Basketball After Hip Arthroscopy: Minimum 2-Year Follow-up
Chen, A. W.;Craig, M. J.;Yuen, L. C.;Ortiz-Declet, V.;Maldonado, D. R.;Domb, B. G.;	2019	Five-Year Outcomes and Return to Sport of Runners Undergoing Hip Arthroscopy for Labral Tears With or Without Femoroacetabular Impingement
Domb, Benjamin G.;Battaglia, Muriel R.;Perets, Itay;Lall, Ajay C.;Chen, Austin W.;Ortiz-Declet, Victor;Maldonado, David R.;	2019	Minimum 5-Year Outcomes of Arthroscopic Hip Labral Reconstruction With Nested Matched-Pair Benchmarking Against a Labral Repair Control Group
Jackson, T. J.;Hanypsiak, B.;Stake, C. E.;Lindner, D.;El Bitar, Y. F.;Domb, B. G.;	2014	Arthroscopic labral base repair in the hip: Clinical results of a described technique
Ishoi, L.;Thorborg, K.;Kraemer, O.;Holmich, P.;	2019	The association between specific sports activities and sport performance following hip arthroscopy for femoroacetabular impingement syndrome: A secondary analysis of a cross-sectional cohort study including 184 athletes
Lall, Ajay C.;Hammarstedt, Jon E.;Gupta, Asheesh G.;Laseter, Joseph R.;Mohr, Mitchell R.;Perets, Itay;Domb, Benjamin G.;	2019	Effect of Cigarette Smoking on Patient-Reported Outcomes in Hip Arthroscopic Surgery: A Matched-Pair Controlled Study With a Minimum 2-Year Follow-up
Lansdown, Drew A;Ukwuani, Gift;Kuhns, Benjamin;Harris, Joshua D;Nho, Shane J;	2018	Self-reported Mental Disorders Negatively Influence Surgical Outcomes After Arthroscopic Treatment of Femoroacetabular Impingement
Lund, B.;Nielsen, T. G.;Lind, M.;	2017	Cartilage status in FAI patients - results from the Danish Hip Arthroscopy Registry (DHAR)
Maldonado, David R.;Lall, Ajay C.;Laseter, Joseph R.;Kyin, Cynthia;Chen, Jeffrey W.;Go, Cammille C.;Domb, Benjamin G.;	2019	Primary Hip Arthroscopic Surgery With Labral Reconstruction: Is There a Difference Between an Autograft and Allograft?
Maldonado, David R.;Laseter, Joseph R.;Perets, Itay;Ortiz-Declet, Victor;Chen, Austin W.;Lall, Ajay C.;Domb, Benjamin G.;	2019	The Effect of Complete Tearing of the Ligamentum Teres in Patients Undergoing Primary Hip Arthroscopy for Femoroacetabular Impingement and Labral Tears: A Match-Controlled Study
Mygind-Klavsen, Bjarne;Lund, Bent;Nielsen, Torsten Grønbech;Maagaard, Niels;Kraemer, Otto;Hölmich, Per;Winge, Søren;Lind, Martin;	2018	Danish Hip Arthroscopy Registry: predictors of outcome in patients with femoroacetabular impingement (FAI)

Perets, Itay;Chaharbakhshi, Edwin O.;Mansor, Yosif;Ashberg, Lyall J.;Mu, Brian H.;Battaglia, Muriel R.;Lall, Ajay C.;Domb, Benjamin G.;	2019	Midterm Outcomes of Iliopsoas Fractional Lengthening for Internal Snapping as a Part of Hip Arthroscopy for Femoroacetabular Impingement and Labral Tear: A Matched Control Study
Rosinsky, P. J.;Kyin, C.;Lall, A. C.;Shapira, J.;Maldonado, D. R.;Domb, B. G.;	2019	Rate of Return to Sport and Functional Outcomes After Bilateral Hip Arthroscopy in High-Level Athletes
Tahoun, M.;Shehata, T. A.;Ormazabal, I.;Mas, J.;Sanz, J.;Tey Pons, M.;	2017	Results of arthroscopic treatment of chondral delamination in femoroacetabular impingement with bone marrow stimulation and BST-CarGel
Abstract/proceedings only		
Chahal, J.;Thiel, G. S. V.;Mather, R. C.;Lee, S.;Salata, M. J.;Nho, S. J.;	2014	The Minimal Clinical Important Difference (MCID) And Patient Acceptable Symptomatic State (PASS) For The Modified Harris Hip Score And Hip Outcome Score Among Patients Undergoing Surgical Treatment For Femoroacetabular Impingement
Domb, B. G.;Dunne, K. F.;Martin, T.;Gui, C.;Finch, N.;Stake, C. E.;	2015	Return to sports in a general hip arthroscopy cohort: Minimum two-year follow-up
Domb, B. G.;Gupta, A.;Dunne, K. F.;Stake, C. E.;Redmond, J. M.;	2014	Microfracture Of The Hip: A Two-year Follow-up With A Matched-pair Control Group
Domb, B. G.;Stake, C. E.;Finley, Z. J.;Baise, R. A.;Botser, I.;	2013	Two-year outcome of arthroscopic capsular repair of the hip: A prospective matched-pair controlled study
Economopoulos, Kostas John;Kweon, Christopher Y.;	2019	Prospective Randomized Comparison of Capsule Management Techniques During Hip Arthroscopy...AOSSM 2019–American Orthopaedic Society for Sports Medicine Annual Meeting, July 11-14, USA, Boston, MA, USA
Frank, R. M.;Lee, S.;Grzybowski, J. S.;Cvetanovich, G.;Mather, R. C.;Bush-Joseph, C. A.;Salata, M. J.;Nho, S. J.;	2015	Outcomes for hip arthroscopy based on sex and age: A comparative matched-group analysis
Harris, J. D.;	2019	In Symptomatic Femoroacetabular Impingement, Arthroscopic Hip Surgery Improved Outcomes at 8 Months Compared with Physiotherapy and Activity Modification
Jackson, T. J.;Stake, C. E.;El Bitar, Y.;Lindner, D.;Botser, I.;Domb, B. G.;	2013	Surgical dislocation of the hip versus arthroscopic treatment of femoro-acetabular impingment: A prospective comparative study with 2-year follow-up
Krych, A. J.;King, A. H.;Berardelli, R. L.;Sousa, P. L.;Levy, B. A.;	2015	Is MRI subchondral acetabular edema or cystic change a contraindication for hip arthroscopy in patients with FAI?
Lindner, D.;Stake, C. E.;Jackson, T. J.;El Bitar, Y.;Chen, A.;Domb, B. G.;	2013	Two year follow-up of hip arthroscopies: A match-controlled study comparing patients over 50 years to under 30 years
Lodhia, P.;Martin, T.;Gui, C.;Stake, C. E.;Vemula, S. P.;Suarez-Ahedo, C.;Chandrasekaran, S.;Domb, B. G.;	2015	Outcomes of 1038 hip arthroscopies: A two-year follow-up study
Nawabi, D. H.;Bedi, A.;Ranawat, A. S.;Kelly, B. T.;	2015	Outcomes of hip arthroscopy for patients with symptomatic borderline dysplasia: A comparison to a matched cohort of patients with symptomatic FAI
Redmond, J. M.;Schwartz, A. R.;Gupta, A.;Stake, C. E.;Finch, N.;Domb, B. G.;	2015	A matched-pair controlled study of arthroscopic psoas tenotomy with minimum 2-year follow-up: Do patients with psoas tenotomy achieve similar outomes?
Thorey, F.;Malahias, M. A.;Giotis, D.;	2019	Sustained benefit of autologous matrix-induced chondrogenesis for hip cartilage repair in a recreational athletic population
Greater than 10% of cohort with dysplasia or other pathologies		
Cooper, Anthony Philip;Basheer, Sheba Z.;Maheshwari, Rajan;Regan, Laura;Madan, Sanjeev S.;	2013	Outcomes of hip arthroscopy. A prospective analysis and comparison between patients under 25 and over 25 years of age
Larson, C. M.;Pierce, B. R.;Giveans, M. R.;	2011	Treatment of athletes with symptomatic intra-articular hip pathology and athletic pubalgia/sports hernia: A case series

Lee, S.;Cvetanovich, G. L.;Mascarenhas, R.;Wuerz, T. H.;Mather, R. C.;Bush-Joseph, C. A.;Nho, S. J.;	2017	Ability to return to work without restrictions in workers compensation patients undergoing hip arthroscopy
Polesello, G. C.;Keiske Ono, N.;Bellan, D. G.;Honda, E. K.;Guimaraes, R. P.;Junior, W. R.;Do Val Sella, G.;	2009	HIP ARTHROSCOPY IN ATHLETES
Uchida, S.;Hatakeyama, A.;Kanezaki, S.;Utsunomiya, H.;Suzuki, H.;Mori, T.;Chang, A.;Matsuda, D. K.;Sakai, A.;	2017	Endoscopic shelf acetabuloplasty can improve clinical outcomes and achieve return to sports-related activity in active patients with hip dysplasia
Degen, R. M.;Mayer, S. W.;Fields, K. G.;Coleman, S. H.;Kelly, B. T.;Nawabi, D. H.;	2017	Functional Outcomes and Cam Recurrence After Arthroscopic Treatment of Femoroacetabular Impingement in Adolescents
Fabricant, P. D.;Heyworth, B. E.;Kelly, B. T.;Fabricant, Peter D.;Heyworth, Benton E.;Kelly, Bryan T.;	2012	Hip arthroscopy improves symptoms associated with FAI in selected adolescent athletes
Mohan, R.;Johnson, N. R.;Hevesi, M.;Gibbs, C. M.;Levy, B. A.;Krych, A. J.;	2017	Return to Sport and Clinical Outcomes After Hip Arthroscopic Labral Repair in Young Amateur Athletes: Minimum 2-Year Follow-Up
Wylie, J. D.;Beckmann, J. T.;Maak, T. G.;Aoki, S. K.;	2015	Arthroscopic treatment of mild to moderate deformity after slipped capital femoral epiphysis: intra-operative findings and functional outcomes
Greater than 10% of the cohort undergoing revision surgery		
Boykin, Robert E.;Patterson, Diana;Briggs, Karen K.;Dee, Ashley;Philippon, Marc J.;	2013	Results of Arthroscopic Labral Reconstruction of the Hip in Elite Athletes
Domb, Benjamin G.;El Bitar, Youssef F.;Stake, Christine E.;Trenga, Anthony P.;Jackson, Timothy J.;Lindner, Dror;	2014	Arthroscopic Labral Reconstruction Is Superior to Segmental Resection for Irreparable Labral Tears in the Hip: A Matched-Pair Controlled Study With Minimum 2-Year Follow-up
Domb, Benjamin G;Martin, Timothy J;Gui, Chengcheng;Chandrasekaran, Sivashankar;Suarez-Ahedo, Carlos;Lodhia, Parth;	2018	Predictors of Clinical Outcomes After Hip Arthroscopy: A Prospective Analysis of 1038 Patients With 2-Year Follow-up
Hartigan, David E;Perets, Itay;Close, Mary R;Walsh, John P;Chaharbakhshi, Edwin O;Mohr, Mitchell R;Domb, Benjamin G;	2018	Arthroscopic Treatment of Iliopsoas Snapping in Patients With Radiographic Acetabular Dysplasia Using Iliopsoas Fractional Lengthening and Capsular Plication
Locks, Renato;Utsunomiya, Hajime;Briggs, Karen K.;McNamara, Shannen;Chahla, Jorge;Philippon, Marc J.;	2018	Return to Play After Hip Arthroscopic Surgery for Femoroacetabular Impingement in Professional Soccer Players
Philippon, Marc J;Bolia, Ioanna K;Locks, Renato;Briggs, Karen K;	2018	Labral Preservation: Outcomes Following Labrum Augmentation Versus Labrum Reconstruction
Philippon, Marc;Schenker, Mara;Briggs, Karen;Kuppersmith, David;	2007	Femoroacetabular impingement in 45 professional athletes: associated pathologies and return to sport following arthroscopic decompression
Thomas, Darren D.;Bernhardson, Andrew S.;Bernstein, Ethan;Dewing, Christopher B.;	2017	Hip Arthroscopy for Femoroacetabular Impingement in a Military Population
White, B. J.;Stapleford, A. B.;Hawkes, T. K.;Finger, M. J.;Herzog, M. M.;	2016	Allograft use in arthroscopic labral reconstruction of the hip with front-to-back fixation technique: Minimum 2-year follow-up
Mixed arthroscopic and open Surgery		
Fitzgerald Jr, R. H.;	1995	Acetabular labrum tears: Diagnosis and treatment
Mannion, A. F.;Impellizzeri, F. M.;Naal, F. D.;Leunig, M.;	2013	Fulfilment of patient-rated expectations predicts the outcome of surgery for femoroacetabular impingement
