# Supplementary material for: Physical Activity Following Hip Arthroscopy in Young and Middle-Aged Adults: A Systematic Review
Source: Sports Med Open. 2020 Jan 28;6:7. doi: 10.1186/s40798-020-0234-8 (PMC6987281; doi:10.1186/s40798-020-0234-8)
Supplement: Supplementary file 4 — Additional file 4: Characteristics and outcomes of included studies. [file 40798_2020_234_MOESM4_ESM.pdf]

Additional file 4- Characteristics and outcomes of included studies

	n Baseline/Final follow up	Women %(n)	Age in years mean± SD*	Inclusion pathology	Physical activity attributes %(n)	Activity category	Outcome	Reported duration of follow-up	Category (months)	Pre-intervention mean±SD	Final score mean±SD	Effect size[95%CI]	Study conclusions where effect size unable to be calculated
Randomised Controlled Trials													
Bennell et al. [131]	14/11 Group 1 [PT rehab]	14%(2)	31±7	FAI/FAIS	Elite/sub-elite (international /national) 36%(5); State 50%(7); Recreational 13%(2); Never competed (0)	NR	HOS-SS	24 wk	≤ 6	50.9±17.1	85.0±17.8	-1.89[-2.87 to -0.92]	
	16/11 Group 2 [no PT rehab]	25%(2)	29±8	FAI/FAIS	Elite/sub-elite (international /national) 38%(6); State 19%(3); Recreational 31%(5); Never competed 13%(2)	NR	HOS-SS	24 wk	≤ 6	52.1±16.7	86±12.4	-2.17[-3.16 to -1.18]	
	Group 1						HAGOS-SR	24 wk	≤ 6	35.9±16.9	81.5±23.4	-2.21[-3.24 to -1.17]	
	Group 2						HAGOS-SR	24 wk	≤ 6	43.9±19.3	78.4±18.6	-1.76[-2.68 to -0.84]	
	Group 1						Tegner	24 wk	≤ 6	3.9±1.8	5.5±1.6	-0.90[-1.74 to -0.07]	
	Group 2						Tegner	24 wk	≤ 6	4.3±2.2	5.6±1.6	-0.64[-1.43 to 0.15]	
	Group 1						HSAS	24 wk	≤ 6	31.0±18.0	31.0±8.5	0.00[-0.79 to 0.79]	
	Group 2						HSAS	24 wk	≤ 6	31.9±21.6	34.4±17.5	-0.12[-0.89 to 0.65]	
Mansell et al. [100]	66/66 [Surgical only]	41%(27)	30±7	FAI/FAIS	NR	NR	HOS-SS	2 yr	19-24	52.6±17.0	57.3±27.5	-0.21[-0.55 to 0.13]	
Prospective Studies, more than 1 arm (Only groups meeting criteria reported)													
Chaharbakhshi et al [43]	20/20 Group 1 [Lig Teres tear]	90%(18)	30±12	Borderline dysplasia; Lig Teres tear	NR	NR	HOS-SS	54.3±17.3 mo	≥ 25	44.1±22.8	68.1±28.9	-0.90[-1.56 to -0.25]	
	20/20 Group 2 [No tear]	90%(18)	27±12	Boarderline dysplasia	NR	NR	HOS-SS	38.6±13.7 mo	≥ 25	50.4±23.9	75.6±19.6	-1.13[-1.80 to -0.46]	
Domb et al. [58]	62/62 HIPS Group 1	60%(37)	42±12	FAI/FAIS; Labral tear	NR	NR	HOS-SS	69.3±7.8 mo	≥ 25	46.7±22.8	73.6±26.9	-1.07[-1.45 to -0.69]	

	n Baseline/Final follow up	Women %(n)	Age in years mean± SD*	Inclusion pathology	Physical activity attributes %(n)	Activity category	Outcome	Reported duration of follow-up	Category (months)	Pre-intervention mean±SD	Final score mean±SD	Effect size[95%CI]	Study conclusions where effect size unable to be calculated
	[Tönnis grade 1]												
	62/62 HIPS Group 2 [Tönnis grade 0]	60%(37)	42±12	FAI/FAIS	NR	NR	HOS-SS	72.1±7.9 mo	≥ 25	46.4±24.7	78.2±17.8	-1.47[-1.87 to -1.07]	
Flores et al. [69]	30/30 HIPS Group1 [Early career]	50%(15)	37±11.5	FAI/FAIS	NR	NR	HOOS-SR	15.5±4.7 mo	13 to 18	36.3±27.2	65.2±27.0	-1.05[-1.59 to -0.51]	
	30/30 HIPS Group2 [Late career]	43%(13)	35±11	FAI/FAIS	NR	NR	HOOS-SR	13.1±2.7 mo	13 to 18	44.5±25.2	75.6±28.9	-1.13[-1.68 to -0.58]	
Flores et al. [70]	39/39 HIPS Group 1 [Retro-version]	59%(23)	31±11	FAI/FAIS; Acetabular retro-version	NR	NR	HOOS-SR	1 yr	7 to 12	38.9±22.1	78.7±16.6	-2.02[-2.57 to -1.47]	
	39/39 HIPS Group 2 [Pincer]	59%(23)	34±8	FAI/FAIS; Focal pincer	NR	NR	HOOS-SR	1 yr	7 to 12	41.9±25.5	77.9±22.9	-1.47[-1.97 to -0.97]	
Glaws et al. [76]	42/28	54%(15)	25±10	FAI/FAIS	Professional; Recreational; High school; College	NR	HOS-SS	6 mo	≤ 6	50.8±21.7	74.8±22.7	-1.07[-1.63 to -0.50]	
Kemp et al. [89]	100/66	49%(49)	36±10	FAI/FAIS +/- Chondropathy	NR	NR	HOOS-SR	30 mo	≥ 25	72.5±23.6	74.9±26.2	-0.10[-0.44 to -0.24]	
Kierkegaard et al.[154]	60/41	63%(38)	36±9	FAI/FAIS	NR	NR	HAGOS-SR	1 yr	7 to 12	*(Median IQR) 31 (20; 48)*	*59 (41; 78)*		Statistically significant change (P <0.001)
							HAGOS-PA	1 yr	7 to 12	*13(0; 31)*	*25 (13; 56)*		Statistically significant change (P <0.001)
							Self-reported PA hr/week	1 yr	7 to 12	*1 (0; 4)*	*4.0 (2; 6)*		Level of significance not reported

	n Baseline/Final follow up	Women %(n)	Age in years mean± SD*	Inclusion pathology	Physical activity attributes %(n)	Activity category	Outcome	Reported duration of follow-up	Category (months)	Pre-intervention mean±SD	Final score mean±SD	Effect size[95%CI]	Study conclusions where effect size unable to be calculated
							Accelerometer data examples						
							% high activity	1 yr	7 to 12	*4.0 (2; 6)*	*4 (3; 5)*		All activity metrics identified as no significant change.
							Steps running	1 yr	7 to 12	*7 (0; 63)*	*23 (0; 108)*		
							Bicycling rotations	1 yr	7 to 12	*45 (8; 434)*	*123 (11; 762)*		
Krych et al. [91]	18/18 Group 1 [Repair]	100%(18)	38(20-59)	FAI/FAIS; Labral tear	NR	NR	HOS-SS	32(12-48) mo	≥ 25	47.5±NR	88.7±NR		
	18/18 Group 2 [Debridement]	100%(18)	39(19-55)	FAI/FAIS; Labral tear	NR	NR	HOS-SS	32(12-48) mo	≥ 25	40.6±NR	76.3±NR		
Newman et al. [104]	492/492 [Primary surgery]	59%(290)	32±10	Non specified	NR	NR	HOS-SS	2 yr (min)	19 to 24	48.0±24.0	77.1±26.0	-1.16[-1.30 to -1.03]	
Redmond et al. [113]	85/85 HIPS Group 1 [No labral detachment]	71%(60)	33±13	FAI/FAIS; Labral tear	NR	NR	HOS-SS	2 yr	19 to 24	45.0±26.1	75.1±28.0	-1.11[-1.43 to -0.78]	
	105/105 HIPS Group 2 [Labral detachment]	57%(60)	33±12	FAI/FAIS; Labral tear	NR	NR	HOS-SS	2 yr	19 to 24	40.1±23.3	74.1±25.4	-1.39[-1.69 to -1.09]	
Redmond et al. [114]	104/91 Group 1 [+PRP]	70%(73)	36±NR	Labral tear	NR	NR	HOS-SS	2 yr (min)	19 to 24	41.3± NR	67.5±NR		Statistically significant change (P <0.05), both groups.
	202/180 Group 2 [No PRP]	64%(130)	36.5	Labral tear	NR	NR	HOS-SS	2 yr (min)	19 to 24	43.5± NR	69.1±NR		
Thorborg et al. [18]	97/76	58% (56)	37(19-59)	FAI/FAIS +/- Labral tear	NR	NR	HAGOS SR (unadjust	12 mo	7 to 12	39.0±19.7	70.5±23.8	-1.45[-1.79 to -1.11]	

	n Baseline/Final follow up	Women %(n)	Age in years mean± SD*	Inclusion pathology	Physical activity attributes %(n)	Activity category	Outcome	Reported duration of follow-up	Category (months)	Pre-intervention mean±SD	Final score mean±SD	Effect size[95%CI]	Study conclusions where effect size unable to be calculated
							ed scores)						
							HAGOS PA (unadjusted scores)	12 mo	7 to 12	19.8±24.2	54.9±37.3	-1.14[-1.47 to -0.82]	
Zimmerer et al. [130]	20/NR Group 1 [Sitting]	28% (12)	25(19-30)	FAI/FAIS	Work activity -sitting	NR	HOOS-SR	24(18-32) mo [Whole cohort]	19 to 24 [Whole cohort]	45.6± NR	80.0±NR		Statistically significant change for group 1 (p=<0.001) and group 2 (p=0.004). Change not statistically significant for group 3 (p=0.186)
	13/NR Group 2 [Standing]				Work activity - standing	NR				36.1± NR	59.6±NR		
	10/NR Group 3 [Active]				Work activity - physical	NR				34.8± NR	57.1±NR		
**Prospective Studies, single arm**													
Bennett et al. [42]	101/97	26%(26)	33(20-50)	FAI/FAIS	Military personnel	NR	FAA	1 yr	7 to 12	2.8±1.0	2.2±1.1	0.57[0.28 to 0.86]	
Chahal et al [37]	130/130	58%(75)	36±12	FAI/FAIS	NR	NR	HOS-SS	2 yr	8 to 12	43.2±26.2	75.4±19.7	-1.39[-1.66 to -1.11]	
Davis et al. [53]	42/28	54%(15)	26±10	FAI/FAIS	Participants in cutting, jumping, pivoting, or lateral movement activities for at least 50 hours per year prior to the onset of hip symptoms	NR	HOS-SS	180 ±32 days	≤ 6	50.8±21.7	74.7±21.8	-1.09[-1.60 to -0.57]	
Domb et al. [63]	43/43 HIPS	35%(15)	44±10	Acetabular chondral defects	NR	NR	HOS-SS	67.6±8.2 mo	≥ 25	40.2±23.2	62.3±30.5	-0.81[-1.25 to -0.37]	

	n Baseline/Final follow up	Women %(n)	Age in years mean± SD*	Inclusion pathology	Physical activity attributes %(n)	Activity category	Outcome	Reported duration of follow-up	Category (months)	Pre-intervention mean±SD	Final score mean±SD	Effect size[95%CI]	Study conclusions where effect size unable to be calculated
Ishoi et al [17]	189/108 [HAGOS scores]	49%(93)	24±3	FAI/FAIS	Mixed levels athlete (elite, competitive, recreational); Whole cohort 'actively involved in sport'	Contact; Noncontact + pivoting; Noncontact + nonpivoting	HAGOS-SR	33.1±16.3 mo	≥ 25	43.4±24.0	61.1±29.5	-0.66[-0.93 to -0.38]	
							HAGOS-PA	33.1±16.3 mo	≥ 25	21.2±25.1	48.7±27.5	-1.04[-1.33 to -0.76]	
Öhlin et al.[150]	361/184	40%(74)	38±13	FAI/FAIS	NR	NR	HAGOS-SR	60 mo	≥ 25	41.1±22.1	66.4±29.9	-0.96 [-1.18 to -0.74]	
							HAGOS-PA	60 mo	≥ 25	30.8±28.2	60.2±33.1	-0.95 [-1.17 to-0.74]	
Philippon [132]	112/90		41	FAI/FAIS	NR	NR	HOS-SS	2.3(2.0-2.9) yr	≥ 25	43.0±NR	69.0±NR		Change HOS-SS = -24 (95% CI -32 to -16)
Sansone et al. [118]	85/85	55%(62)	25±5	FAI/FAIS	Elite Sub elite National International	Cutting (79%); Flexibility; Contact; Impingement; Asymmetric /overhead; Endurance	HAGOS-SR	12.3±0.6 mo	7 to 12	39.0±21.0	75.0±23.0	-1.63[-1.98 to -1.28]	
							HAGOS-PA	12.3±0.6 mo	7 to 12	27.0±28.0	70.0±30.0	-1.48[-1.82 to -1.14]	
							HSAS			4.3±2.5	5.7±2.2	-0.63[-0.94 to 0.33]	
Sansone et al.[6]	394/289	34%(134)	37±13	FAI/FAIS	NR	NR	HAGOS-SR	25±2 mo	≥ 25	40.0±20.0	65.0±29.0	-1.00[-1.18 to -0.83]	
							HAGOS-PA	25±2 mo	≥ 25	29.0±26.0	57.0±34.0	-0.92[-1.10 to -0.75]	
							HSAS			2.9±2.2	3.6±2.1	-0.33[-0.49 to -0.16]	
Tahoun et al. [123]	23/23	22%(5)	41±7	FAI/FAIS; Chondral defect	Tegner 6±1.5 [range 3 to 10]	NR	HOS-SS	38.4±7 mo	≥ 25	30.9±13.9	70.8±26.2	-1.87[-2.57 to -1.17]	

	n Baseline/Final follow up	Women %(n)	Age in years mean± SD*	Inclusion pathology	Physical activity attributes %(n)	Activity category	Outcome	Reported duration of follow-up	Category (months)	Pre-intervention mean±SD	Final score mean±SD	Effect size[95%CI]	Study conclusions where effect size unable to be calculated
Tijssen et al. [124]	45/37	43%(16)	40.5±9	Not specified	Recreational	Cutting (19%); Endurance (30%); Other (38%); No sport (13%)	iHot – 33 SR	26.8±11.6 mo	≥ 25	NR±NR	60.5±27.5		Statistically significant decrease in sport frequency (p=0.04) **pre-injury** to post-op.
							Tegner			NR±NR	6.2±1.9		
Retrospective Studies, more than 1 arm (Only groups meeting criteria reported)													
Basques et al.[133]	707/624	65%(406)	34±14	FAI/FAIS	"Regular exercise" – not defined 71.6% of cohort	NR	HOS-SS	2 yr	19 to 24	44.9±22.8	71.3±27.1	-1.05 [-1.17 to -0.94]	
Beck et al.[135]	112/112	72%(81)	34 ±13	FAI/FAIS	NR	NR	HOS-SS	32.9±9.3 mo	≥25	41.7±20.5	72.6±27.1	-1.28 [-1.57 to -0.99]	
	224/224 Group 2 [	68%(153)	34± 13	FAI/FAIS	NR	NR	HOS-SS	32.9±9.3 mo	≥25	43.9±22.8	74.7±26.1	-1.25 [-1.46 to -1.05]	
Bolia et al [136]	42/42 Group 1 [no capsular repair]	43%(18)	38±15	FAI/FAIS, Labral tear	NR	NR	HOS-SS	7.3±2.7 yr	≥25	43±25	74±24	-1.25 [-1.72 to -0.78]	
	84/84 Group 2 [capsular repair]	43%(36)	38±15	FAI/FAIS, Labral tear	NR	NR	HOS-SS	6.4±2.3 yr	≥25	48±24	79±21	-1.37 [-1.71 to -1.03]	
Cancienne et al [137]	120/120	61%(73)	37±6	FAI/FAIS	NR	NR	HOS-SS	33.7±3.1 mo	≥25	46±24.2	74.7±22.2	-1.23 [-1.51 to -0.96]	
Chaharbakhshi et al[138]	16/12 Group 1[anteversion and dysplasia]	100%(12)	29±13	Borderline dysplasia	NR	NR	HOS-SS	44.2±23.4 mo	≥25	34.9±23.9	58.3±37.5	-0.72 [-1.55 to 0.11]	

	n Baseline/Final follow up	Women %(n)	Age in years mean± SD*	Inclusion pathology	Physical activity attributes %(n)	Activity category	Outcome	Reported duration of follow-up	Category (months)	Pre-intervention mean±SD	Final score mean±SD	Effect size[95%CI]	Study conclusions where effect size unable to be calculated
	24/24 Group 2 [Control]	100%(24)	28±13	Not specified	NR	NR	HOS-SS	44.2±16.5 mo	≥25	46.1±23.4	78.4±22	-1.40 [-2.04 to -0.76]	
Chahla et al [140]	267/267 Group1 [small tear]	82%(218)	32±12	FAI/FAIS, Labral tear	'Runners'	Endurance	HOS-SS	2 yr	19 to 24	41.6±22.3	76.6±23.5	-1.53 [-1.72 to -1.33]	
	333/333 [large tear]	51%(169)	35±12	FAI/FAIS, Labral tear	'Runners'	Endurance	HOS-SS	2 yr	19 to 24	42.6±22.9	70.5±27.7	-1.10 [-1.26 to -0.93]	
Chandrasekaran et al. [49]	12/10 HIPS Group 1 [Lower index score]	58%(7)	45±8	Labral tear +/- FAI/FAIS	NR	NR	HOS-SS	2 yr	19 to 24	41.8±NR	45.3±NR		No statistically significant change in scores group 1 (p=0.788). Statistically significant change group 2 (p<0.001)
	52/42 HIPS Group 2 [Higher index score]	85%(44)	41±13	Labral tear +/- FAI/FAIS	NR	NR	HOS-SS	2 yr	19 to 24	36.3±NR	67.9±NR		
Chandrasekaran et al. [47]	93/93 Group 1 [Tönnis 1]	48%(45)	41(16-64)	Mild OA (Tönnis grade 1)	NR	NR	HOS-SS	28(23-67.9) mo	≥ 25	40.2±NR	60.9±NR		Statistically significant change for both groups (p<0.001)
	93/93 Group 2 [Tönnis 0]	48%(45)	41(15-63)	Tönnis grade 0	NR	NR	HOS-SS	31.5(23.6-63.5) mo	≥ 25	39.7±NR	61.3±NR		
Chandrasekaran et al. [44]	36/36 Group1 [Over-coverage]	50%(18)	31(16-50)	Labral tear; Acetabular over-coverage	NR	NR	HOS-SS	31.5(21.3-46.2) mo	≥ 25	46.0±26.5	63.2±33.0	-0.57[-1.04 to -0.10]	
	36/36 Group2 [Normal coverage]	50%(18)	32(16-33)	Labral tear; Normal acetabular coverage	NR	NR	HOS-SS	29.3(20.7-46.9) mo	≥ 25	40.5±24.6	69.0±32.0	-0.99[-1.48 to -0.50]	
Chandrasekaran et al [142]	57/57 Group 1 [Lumbar surgery]	56%(32)	46(21-69)	Not specified	NR	NR	HOS-SS	27.6±NR mo	≥ 25	22.8±21.8	50.6±31.5	-1.02 [-1.41 to -0.63]	
	57/57 Group 2 [Control]	56%(32)	46(23-73)	Not specified	NR	NR	HOS-SS	28.5±NR mo	≥ 25	38.1±27	60.9±32.8	-0.75 [-1.13 to -0.37]	

	n Baseline/Final follow up	Women %(n)	Age in years mean± SD*	Inclusion pathology	Physical activity attributes %(n)	Activity category	Outcome	Reported duration of follow-up	Category (months)	Pre-intervention mean±SD	Final score mean±SD	Effect size[95%CI]	Study conclusions where effect size unable to be calculated
Chen et al. [50]	101/69 HIPS Group 1 (SDLP)	74%(75)	44±15	Labral tear	NR	NR	HOS-SS	66.5±8 mo	≥ 25	57.4±19.6	83.6±17.2	-1.41[-1.79 to -1.04]	
Clapp et al [143]	59/59 Group 1 [Athletes]	61%(37)	23±5	FAI/FAIS	Competitive	Cutting (32%); Flexibility (12%); Contact (3%); Asymmetric /overhead (31%); Endurance (10%)	HOS-SS	2 yr	19 to 24	45.7±18.2	84.5±19	-2.07 [-2.52 to -1.62]	
	118/118 Group 2 [Non athletes]	73%(85)	24±3	FAI/FAIS	Non-competitive	NR	HOS-SS	2 yr	19 to 24	41.3±20.7	76.1±23.8	-1.56 [-1.85 to -1.26]	
Cvetanovich et al [51]	36/36 Group 1 [Boarderline dysplasia]	75%(27)	32±12	FAI/FAIS; Borderline dysplasia	NR	Endurance (58%)	HOS-SS	2.6±0.6 yr	≥ 25	44.5±20.9	73.6±26.7	-1.20[-1.70 to -0.70]	
	312/312 Group 2 [Normal coverage]	57%(177)	33±12	FAI/FAIS	NR	Endurance (60%)	HOS-SS	2.6±0.6 yr	≥ 25 mo	42.8±23.3	73.1±27.1	-1.20[-1.37 to -1.03]	
Degen et al. [55]	12/12 Group 1 [Simultaneous bilateral]	42%(5)	21±5	FAI/FAIS	NR	NR	HOS-SS	16.4 mo	13 to 18	62.7±21.7	93.3±10.2	-1.74[-2.71 to -0.78]	
	24/24 Group 2 [Staged bilateral]	42%(10)	21±5	FAI/FAIS	NR	NR	HOS-SS	17.8 mo	13 to 18	54.3±22.2	83.9±20.5	-1.36[-2.00 to -0.73]	
Domb et al. [64]	20/20 [Arhtroscopic]	80%(16)	20	FAI/FAIS	NR	NR	HOS-SS	25.5 mo	≥ 25	44.3±NR	87.1±12.1		
Domb et al. [60]	21/21 Group1 [Non-workcover]	40%(12) [whole cohort]	45	Labral tear; Full thickness cartilage defect	NR	NR	HOS-SS	35(24-50) mo	≥ 25	38.1±NR	69.5±NR		Statistically significant change in both groups (p<0.05).

	n Baseline/Final follow up	Women %(n)	Age in years mean± SD*	Inclusion pathology	Physical activity attributes %(n)	Activity category	Outcome	Reported duration of follow-up	Category (months)	Pre-intervention mean±SD	Final score mean±SD	Effect size[95%CI]	Study conclusions where effect size unable to be calculated
	9/9 Group 2 [Workcover]			Labral tear; Full-thickness cartilage defect	NR	NR	HOS-SS	35(24-50) mo	≥ 25	22.9±NR	54.0±NR		
Domb et al. [62]	52/52 [≤30]	65%(34)	20(13-30)	Not specified	NR	NR	HOS-SS	2.7 yr	≥ 25	42.2±NR	72.7±NR		Statistically significant change (P<0.001).
Domb et al. [65]	235/235 Group 1 [Release]	41%(97)	42±12	Intra-articular	NR	NR	HOS-SS	2.2±0.4 yr	≥ 25	36.9±26.2	67.3±29.4	-1.09[-1.28 to -0.89]	
	168/168 Group 2 [Repair]	81%(136)	29±12	Intra-articular	NR	NR	HOS-SS	2.1±0.3 yr	≥ 25	46.4±23.6	71.3±27.7	-0.96[-1.19 to -0.74]	
Domb et al. [59]	88/88 Group 1 [Returned to sport]	63%(67) [HIPS]	31(13-61)	Not specified	Professional; Recreational; High school; College	Cutting; Contact; Impingement; Asymmetric /overhead; Endurance	HOS-SS	2 yr (min)	19 to 24	44.9± NR	76.0±NR		
	60/60 Group 2 [Not returned to sport]	53%(35) [HIPS]	30(14-59)	Not specified	Professional; Recreational; High school; College		HOS-SS	2 yr (min)	19 to 24	41.9±NR	62.0±NR		
Domb et al. [61]	926/824 HIPS Group 1 [Primary]	58%(540)	37(17-76)	Not specified	NR	NR	HOS-SS	28.8(23.5-76.3) mo	≥ 25	NR±NR	80.0±NR		Pre to post-op mean change HOS-SS = 23.8
Domb et al. [56]	65/65 Group 1 [Capsular release]	72%(47)	38±13	Labral tear	NR	NR	HOS-SS	75.7±8.6 mo	≥ 25	43.6±23.9	76.1±24.4	-1.34[-1.72 to -0.96]	
	65/65 Group 2 [Capsular closure]	72%(47)	37±12	Labral tear	NR	NR	HOS-SS	64.8±4.2 mo	≥ 25	45.0±27.7	68.1±27.4	-0.83[-1.19 to -0.47]	
Fabricant et al [68]	243/210 [Whole cohort, those completing HOS]	51%(123)	28±9	FAI/FAIS	NR	NR	HOS-SS	1 yr (min)	7 to 12	NR±NR	NR±NR		Pre to post-op change HOS-SS =23 (95%CI 19 to 27)
Frank et al. [71]	32/32 Group 1 [Partial closure]	63%(20)	33±10	FAI/FAIS	NR	NR	HOS-SS	30.1±2.9 mo	≥ 25	39.4±23.9	83.6±9.6	-2.40[-3.05 to -1.75]	

	n Baseline/Final follow up	Women %(n)	Age in years mean± SD*	Inclusion pathology	Physical activity attributes %(n)	Activity category	Outcome	Reported duration of follow-up	Category (months)	Pre-intervention mean±SD	Final score mean±SD	Effect size[95%CI]	Study conclusions where effect size unable to be calculated
	32/32 Group 2 [Complete closure]	63%(20)	33±10	FAI/FAIS	NR	NR	HOS-SS	29.7±2.5 mo	≥ 25	39.1±24.2	87.3±8.3	-2.63[-3.31 to -1.95]	
Frank et al. [72]	75/75 Group 1 [Females]	100%(75)	38±14	FAI/FAIS	NR	NR	HOS-SS	33.64 ± 5.7 mo	≥ 25	40.6±22.1	81.2±14.9	-2.14[-2.54 to -1.74]	
	75/75 Group 2 [Males]	0%(0)	37±12	FAI/FAIS	NR	NR	HOS-SS	33.64 ± 5.7 mo	≥ 25	46.7±26.3	86.3±11.6	-1.94[-2.33 to -1.55]	
Frank et al [144]	97/97 Group 1 [Athletes]	100%(97)	36±10	FAI/FAIS	Recreational (88)%; High school (8%); Collegiate (3%); Professional (<1%)	Cutting; Flexibility; Asymmetric /overhead; Endurance	HOS-SS	2.6±1 yr	≥ 25	39.9±20.7	82.2±18.5	-2.15 [-2.50 to -1.79]	
	97/97 Group 2 [Non-athletes]	100%(97)	38±10	FAI/FAIS	Non-athletes	NR			≥ 25	32.3±24.1	49.2±34.1		
Gupta et al. [77]	87/62 Group1 [Obese]	73%(45)	42(17-61)	Not specified	NR	NR	HOS-SS	2.7 yr	≥ 25	25.4±22.3	55.5±32.4	-1.08[-1.45 to -0.70]	
	364/124 Group 2 [Control]	72%(90)	42(17-65)	Not specified	NR	NR	HOS-SS	2.5 yr	≥ 25	42.0±24.2	71.4±27.2	-1.14[-1.41 to -0.87]	
Hartigan et al. [80]	59/59 Group 1 [Retro-version]	61%(36)	36±15	Femoral retro-version	NR	NR	HOS-SS	37.6±14.9 mo	≥ 25	45.7±25.5	69.4±28.0	-0.88[-1.26 to -0.50]	
	59/59 Group 2 [Normal version]	68%(40)	36±13	Not specified	NR	NR	HOS-SS	37.9±13 mo	≥ 25	44.8±23.8	65.3±33.1	-0.71[-1.08 to -0.33]	
Hartigan et al. [79]	15/15 Group 1 [Micro-fracture]	47%(7)	45±9	Femoral head chondral damage (grade IV)	NR	NR	HOS-SS	36.8±16.3 mo	≥ 25	26.7±21.6	57.2±25.9	-1.24[-2.04 to -0.45]	
	45/45 Group 2 [No micro-fracture]	47%(21)	44±8	Not specified	NR	NR	HOS-SS	40.6±18 mo	≥ 25	42.3±26.1	66.7±28.9	-0.88[-1.31 to -0.45]	
Hassebrock et al [145]	133/133 Group 1[First hip]	65%(86)	32(29-34)	FAI/FAIS, Labral tear	NR	NR	HOS-SS	>2 yr	≥ 25	39.3±NR	70.7±NR		

	n Baseline/Final follow up	Women %(n)	Age in years mean± SD*	Inclusion pathology	Physical activity attributes %(n)	Activity category	Outcome	Reported duration of follow-up	Category (months)	Pre-intervention mean±SD	Final score mean±SD	Effect size[95%CI]	Study conclusions where effect size unable to be calculated
	133/133 Group 2 [Second hip]	65%(86)	32(29-34)	FAI/FAIS, Labral tear	NR	NR	HOS-SS	>2 yr	≥ 25	38.5±NR	68.5±NR		
Hevesi et al [83]	96/96 Group 2 [Non-dysplasic	51%(49)	31±12	Labral tear	NR	NR	HOS-SS	5.7 (5.0-7.7) yr	≥ 25	41.1±25.0	71.0±26.6	-1.15[-1.46 to -0.85]	
Hevesi et al [146]	82/82 HIPS Group 1 [	46%(34)	33±11	Labral tear; Chondral damage	NR	NR	HOS-SS	4 (2-8.5) yr	≥ 25	47.1±26.1	75.5±26.4	-0.96 [-1.29 to-0.64]	
	31/31 HIPS Group 2 [	34%(11)	39±9	Labral tear; Chondral damage	NR	NR	HOS-SS	4 (2-8.5) yr	≥ 25	45.6±27.5	66.3±26.5	-0.76 [-1.27 to -0.24]	
Jackson et al. [86]	110/110 Group 1 [Labral base repair]	69%(76)	27	Labral tear	NR	NR	HOS-SS	30(19.2-60) mo	≥ 25	46.0±NR	76.0±NR		Statistically significant change in both groups (p<0.001)
	110/110 Group 2 [Circumferential suture]	69%(76)	27	Labral tear	NR	NR	HOS-SS	30(19.2-67) mo	≥ 25	45.0±NR	76.0±NR		
Jackson et al. [87]	22/22 Group 1 [Femoral retro-version]	77%(17)	38(14-55)	Not specified	NR	NR	HOS-SS	28.4±5.6 mo	≥ 25	46.9±27.4	79.2±17.9	-1.37[-2.03 to -0.71]	
	196/196 Group 2 [Normal ante-version]	62%(121)	38(14-66)	Not specified	NR	NR	HOS-SS	28.3±5.8 mo	≥ 25	42.0±24.6	69.6±28.1	-1.04[-1.25 to -0.83]	
	27/27 Group 3 [Excessive femoral ante-version]	74%(20)	38(15-69)	Not specified	NR	NR	HOS-SS	32.3±6.8 mo	≥ 25	45.8±23.3	72.9±28.7	-1.02[-1.59 to -0.45]	
Krishnamoorthy et al. [147]	21/21 Group 1 [symphysis pubis change]	65%(15)	37±13	FAI/FAIS	NR	NR	HOS-SS	2 yr	19 to 24	41.2±14.7	61.9±37.4	-0.71 [-1.34 to -0.09]	
	42/42 Group 2 [Control]	63%(29)	37±13	FAI/FAIS	NR	NR	HOS-SS	2 yr	19 to 24	46.9±13.9	91.6±14.5	-3.12 [-3.76 to -2.47]	
Kuhns et al. [92]	43/43 Group 1 [Bilateral arthroscopy]	56%(24)	29±11	FAI/FAIS	Recreational or high-level	NR	HOS-SS	2.3±0.37 yr	≥ 25	45.6±24.1	73.4±26.0	-1.10[-1.55 to -0.64]	

	n Baseline/Final follow up	Women %(n)	Age in years mean± SD*	Inclusion pathology	Physical activity attributes %(n)	Activity category	Outcome	Reported duration of follow-up	Category (months)	Pre-intervention mean±SD	Final score mean±SD	Effect size[95%CI]	Study conclusions where effect size unable to be calculated
					amateur sports								
	86/86 Group 2 [Unilateral arthroscopy]	56%(48)	29±11	FAI/FAIS	Recreational or high-level amateur sports	NR	HOS-SS	2.6±0.66 yr	≥ 25	44.9±23.1	71.6±28.1	-1.03[-1.35 to -0.71]	
Kunze et al [148]	1094/1094	66%(721)	32±12	FAI/FAIS	NR	NR	HOS-SS	30.8±6.7 mo	≥ 25	42.5±22.6	74.6±25.5	-1.33 [-1.42 to -1.24]	
Levy et al. [94]	28/28 Group 1 [Atypical presentation]	64%(18)	36±10	FAI/FAIS	NR	NR	HOS-SS	2 yr	19 to 24	42.0±25.5	71.0±26.2	-1.11[-1.67 to -0.54]	
	56/56 Group 2 [Typical presentation]	64%(36)	35±10	FAI/FAIS	NR	NR	HOS-SS	2 yr	19 to 24	44.4±24.9	71.3±27.3	-1.02[-1.42 to -0.63]	
Locks et al. [96]	35/35 Group 2 [Control]	78%(28)	33±14	Labral tear	NR	NR	HOS-SS	3.6±1 yr	≥ 25	38.0±NR	57.0±32.0		Both groups showed significant improvement from pre- to postoperative scores' – values not reported.
Lodhia et al. [97]	35/35 Group 1 [Microfracture]	34%(12)	42(28-53)	FAI/FAIS; &/or Labral tear; Grade IV Outerbridge cartilage defect	NR	NR	HOS-SS	3 yr	≥ 25	42.1±24.2	61.4±26.1	-0.76[-1.24 to -0.27]	
	70/70 Group 2 [Control]	34%(24)	42(24-61)	FAI/FAIS; and/or Labraltear	NR	NR	HOS-SS	3 yr	≥ 25	37.6±25.1	63.7±27.9	-0.98[-1.33 to -0.62]	
Lodhia et al. [98]	49/49 Group 1 [Central acetabular decomp]	43%(21)	49(29-61)	FAI/FAIS; and/or Labral tear	NR	NR	HOS-SS	26.1(23.5-36.5) mo	≥ 25	43.9±22.6	59.1±28.1	-0.59[-1.00 to -0.19]	
	147/147 Group 2 [Control]	43%(63)	48(25-66)	FAI/FAIS; and/or Labral tear	NR	NR	HOS-SS	27.77(23.6-54.9) mo	≥ 25	38.3±24.7	62.3±28.5	-0.90[-1.14 to -0.66]	

	n Baseline/Final follow up	Women %(n)	Age in years mean± SD*	Inclusion pathology	Physical activity attributes %(n)	Activity category	Outcome	Reported duration of follow-up	Category (months)	Pre-intervention mean±SD	Final score mean±SD	Effect size[95%CI]	Study conclusions where effect size unable to be calculated
Nawabi et al. [103]	46/46 Group1 [Borderline dysplasia]	48%(22)	30±9	FAI/FAIS; Borderline dysplasia	NR	NR	HOS-SS	24 mo (min)	19 to 24	54.6±23.0	85.4±22.1	-1.35[-1.81 to -0.90]	
	131/131 Group 2 [Control]	56%(73)	30±10	FAI/FAIS	NR	NR	HOS-SS	23 mo (min)	19 to 24	53.3±23.7	78.8±25.2	-1.04[-1.30 to -0.78]	
Perets et al. [108]	60/41 Group 1 [IFL]	80%(48)	20±4	FAI/FAIS	Professional; High school; Collegiate	NR	HOS-SS	2 yr (min)	19 to 24	44.1±17.7	73.0±24.9	-1.33[-1.81 to -0.85]	
	41/41 Group 2 [Control]	NR	NR	FAI/FAIS	Professional; High school; Collegiate	NR	HOS-SS	NR	NR	NR±NR	NR±NR		
Perets et al. [110]	11/11Group 1 [Calcification]	100%(11)	40±6	FAI/FAIS; Labral tear	NR	NR	HOS-SS	45±19.9 mo	≥ 25	35.4±23.7	62.7±26.1	-1.05[-1.96 to -0.15]	
	11/11 Group 2 [Control]	100%(11)	40±6	FAI/FAIS; Labral tear	NR	NR	HOS-SS	49.8±22.4 mo	≥ 25	45.8±21.5	70.2±24.9	-1.01[-1.91 to -0.11]	
Perets et al. [107]	74/74 Group 1 [Obese BMI ≥30]	61%(45)	44±12	FAI/FAIS; Labral tear	NR	NR	HOS-SS	71.6±10.6 mo	≥ 25	25.2±21.3	62.9±30.8	-1.42[-1.78 to -1.06]	
	74/74 Group 2 [BMI 18.5 to 24.99	61%(45)	44±12	FAI/FAIS; Labral tear	NR	NR	HOS-SS	71.3±9.5 mo	≥ 25	37.7±26.5	70.0±24.7	-1.25[-1.61 to -0.90]	
Saltzman et al. [117]	NR/197 Group 1 [Normal weight]	72%(142)	30±11	FAI/FAIS	78% 'sport activity' - unspecified	NR	HOS-SS	2.6±0.5 yr	≥ 25	43.6±23.2	76.6±24.8	-1.37[-1.59 to 1.15]	
	NR/130 Group 2 [Over-weight]	43%(56)	35±12	FAI/FAIS	69% 'sport activity' - unspecified	NR	HOS-SS	2.6±0.5 yr	≥ 25	43.3±24.7	68.7±29.4	-0.93[-1.19 to -0.68]	
Sawyer et al. [119]	189/189 Group 1 [Looped]	48%(91)	36±11	FAI/FAIS;Labral & chondral damage	NR	NR	HOS-SS	39.6±10.4 mo	≥ 25	50.6±25.3	81.0±20.8	-1.31[-1.53 to -1.09]	
	60/60 Group 2 [Pierced]	48%(29)	36±11	FAI/FAIS; Labral & chondral damage	NR	NR	HOS-SS	36.8±8.9 mo	≥ 25	46.4±22.6	77.1±26.6	-1.24[-1.63 to -0.84]	
	77/77 Group 3 [Combined]	57%(44)	33±11	FAI/FAIS; Labral &	NR	NR	HOS-SS	32.7±7.4 mo	≥ 25	52.4±21.4	79.1±23.5	-1.18[-1.53 to -0.84]	

	n Baseline/Final follow up	Women %(n)	Age in years mean± SD*	Inclusion pathology	Physical activity attributes %(n)	Activity category	Outcome	Reported duration of follow-up	Category (months)	Pre-intervention mean±SD	Final score mean±SD	Effect size[95%CI]	Study conclusions where effect size unable to be calculated
				chondral damage									
Stake et al. [121]	21/21 HIPS Group 1 [Worker's comp]	14%(3)	39(24-55)	Labral tear	NR	NR	HOS-SS	2 yr (min)	19 to 24	15.3±12.8	49.8±28.2	-1.54[-2.24 to -0.85]	Statistically significant change both groups (p<0.001)
	21/21 HIPS Group 2 [No worker's comp]	14%(3)	NR	Labral tear	NR	NR	HOS-SS	2 yr (min)	19 to 24	41.9±21.5	73.8±22.5	-1.42[-2.11 to -0.74]	
Stone et al [152]	100/100 Group 1 [no generalised laxity]	100%(100)	23±9	FAI/FAIS	Routine physical exercise (87%); Running as primary exercise(64%)	NR	HOS-SS	29.3±6 8 mo	≥ 25	NR	NR		Change score 37 ±26.7
	25/25 Group 2 [generalised laxity]	100%(25)	18±6	FAI/FAIS	Routine physical exercise (96%); Running as primary exercise(52%)	NR	HOS-SS	29.3±6 8 mo	≥ 25	NR	NR		Change score 35.1±27.3
Suarez-Ahedo et al. [122]	825/825 Group 1 [<34.6 years]	64%(531)	NR	Not specified	NR	NR	HOS-SS	28.98 mo [whole group]	≥ 25	43.5±25.4	73.4±40.8	-0.88[-0.98 to -0.78]	
	872/872 Group 2 [>34.6 years]	63%(505)	NR	Not specified	NR	NR	HOS-SS			35.6±24.8	61.9±30.8	-0.94[-1.04 to -0.84]	
Vap et al. [125]	72/72 Group 1 [Trochanteric Bursitis]	75%(54)	37	FAI/FAIS	NR	NR	HOS-SS	42±9.9 mo	≥ 25	49.0±24.0	78.0±28.0	-1.11[-1.46 to 0.75]	
	72/72 Group 2 [No trochanteric Bursitis]	75%(54)	37	FAI/FAIS	NR	NR	HOS-SS	42±9.1 mo	≥ 25	45.0±24.0	77.0±27.0	-1.25[-1.60 to -0.89]	

	n Baseline/Final follow up	Women %(n)	Age in years mean± SD*	Inclusion pathology	Physical activity attributes %(n)	Activity category	Outcome	Reported duration of follow-up	Category (months)	Pre-intervention mean±SD	Final score mean±SD	Effect size[95%CI]	Study conclusions where effect size unable to be calculated
Weber et al. [127]	17/17 Group 1 [High level athletes]	53%(9)	18±8	FAI/FAIS	"High level"	Cutting (17%); Flexibility (23%); Contact (12%); Impingement (1%); Asymmetric /overhead (17%); Endurance (30%)	HOS-SS	2 yr (min)	19 to 24	41.9±18.4	83.2±19.6	-2.12[-2.98 to -1.26]	
	49/49 Group 2 [Rec athletes]	63%(31)	30±9	FAI/FAIS	Recreational	NR	HOS-SS	2 yr (min)	19 to 24	41.9±21.6	79.0±23.3	-1.64[-2.10 to -1.18]	
Wu et al. [128]	68/68 Group 1 [No dysplasia]	63%(43)	42±9	Labral tear	NR	NR	HOS-SS	29.1±4.5 mo	≥ 25	50.3±8.9	88.9±5.2	-5.27[-5.98 to -4.55]	
Yoo et al. [129]	28/28 Group 1 [Military]	0%(0)	21±2	FAI/FAIS	Active military service	NR	UCLA	3.5±2.5 yr	≥ 25	6.1±NR	9.4±NR		Statistically significant change in both groups (p <0.001)
	28/28 Group 2 [Non military]	0%(0)	23±3	FAI/FAIS	Active non-military	NR	UCLA	3.7±2 yr	≥ 25	5.4±NR	8.4±NR		
Retrospective Studies, single-arm													
Barastegui et al. [40]	21/21	0%(0)	27±7	FAI/FAIS	Professional	Cutting	HOS-SS	45.4±5.6 mo	≥ 25	37.6±NR	86.7±NR		'Statistically significant differences observed' - values not reported.
Bayley et al. [41]	76/76	67%(51)	20±3	Labral tear	NR	NR	HOOS-SR	1 yr	7 to 12	38.8±27.4	60.5±29.4	-0.76[-1.09 to -0.43]	

	n Baseline/Final follow up	Women %(n)	Age in years mean± SD*	Inclusion pathology	Physical activity attributes %(n)	Activity category	Outcome	Reported duration of follow-up	Category (months)	Pre-intervention mean±SD	Final score mean±SD	Effect size[95%CI]	Study conclusions where effect size unable to be calculated
Beck et al [134]	108/108	NR	41±13	NR	NR	NR	HOS-SS	32.9m±9.3	≥ 25	40.1±26.6	73.4±28.6	-1.20 [-1.49 to -0.91]	
Chahla et al. [139]	189/153	71%(109)	34±13	FAI/FAIS	'Self report - any physical activity'	NR	HOS-SS	2 yr	≥ 25	42.9±21.7	76.3±21.2	-1.55 [-1.81 to -1.30]	
Chambers et al [141]	156/142	49%(70)	36±12	FAI/FAIS	NR	NR	HOOS-SR	1 yr	19 to 24	40.5±27.7	72.9±24.2	-1.41 [-1.67 to -1.15]	
Chandrasekaran et al. [45]	22/22	64%(14)	32±10	Labral tear	NR	NR	HOS-SS	2 yr	19 to 24	42.3±22.3	65.4±28.4	-0.89[-1.51 to -0.27]	
Chandrasekaran et al. [46]	55/52	84%(46)	24(13-38)	Boarderline dysplasia	NR	NR	HOS-SS	25.4(24-30.4) mo	≥ 25	46.6±NR	74.8±NR		Pre to post-op change HOS-SS = 27.6 (95%CI 20.0 to 35.2); p<0.001
Chandrasekaran et al. [48]	1137/1137	74%(840)	37(13-76)	Not specified	NR	NR	HOS-SS	2 yr	19 to 24	41.3±24.2	64.4±30.4	-0.84[-0.93 to -0.75]	
Cvetanovich et al. [52]	474/386	61%(251) [HIPS]	33±12	FAI/FAIS	NR	Endurance (58%);	HOS-SS	2.6±0.6 yr	≥ 25	43.9±23.4	72.2±27.3	-1.11[-1.26 to -0.96]	

	n Baseline/Final follow up	Women %(n)	Age in years mean± SD*	Inclusion pathology	Physical activity attributes %(n)	Activity category	Outcome	Reported duration of follow-up	Category (months)	Pre-intervention mean±SD	Final score mean±SD	Effect size[95%CI]	Study conclusions where effect size unable to be calculated
						'Sport/hobbies' (72%)							
Degen et al. [54]	70/34	0%(0)	22±5	FAI/FAIS	Professional 27.1%(19); College 57.1%)(40) High school 8.6%(6); Club/team 7.1%(5)	Asymmetric /overhead	HOS-SS	2 yr	19 to 24	51.3±24.8	92.3±8.2	-1.95[-2.44 to -1.45]	
Domb et al. [66]	26/22	82%(18)	20(14-39)	Boarderline dysplasia	NR	NR	HOS-SS	27.5±5.5 mo	≥ 25	49.0±15.6	77±21.9	-1.45[-2.12 to -0.78]	
Domb et al. [67]	60/60	73%(47) [HIPS]	29±12	Labral tear	NR	NR	HOS-SS	67.8±7.4 mo	≥ 25	47.1±23.2	76.5±25.9	-1.19[-1.58 to -0.80]	
Domb et al. [57]	24/19	89%(17)	23±8	Boarderline dysplasia	NR	NR	HOS-SS	68.8±6.4 mo	≥ 25 mo	52.1±15.9	70.8±19.5	-1.03[-1.71 to -0.35]	
Flores et al. [35]	122/49	53%(68) [HIPS]	36±11	FAI/FAIS; Labral tear	NR	NR	HOOS-SR	2 yr	19 to 24	39.9±22.4	75.3±23.6	-1.55[-1.92 to -1.18]	
Frank et al. [75]	62/58	62%(36)	30±7	FAI/FAIS	Recreational	Endurance (cycling)	HOS-SS	31.14±0.71 mo	≥ 25	41.5±23.2	85.2±16.0	-2.18[-2.64 to -1.72]	No significant change to average miles cycled/week: 30.3±42.4 [range 2 to 300] pre-op; 23.8±22.9 [range not reported] post-op, (p=0.08).
Frank et al [73]	44/42	90%(38) [HIPS]	35±9	FAI/FAIS	NR	Flexibility	HOS-SS	30.5±12 mo	≥ 25	48.0±23.7	85.9±12.9	-1.96[-2.47 to -1.44]	No significant change to average hours/week of yoga: 2.7±1.9 pre-op; 2.5±1.3 post-op, (p=0.44).

	n Baseline/Final follow up	Women %(n)	Age in years mean± SD*	Inclusion pathology	Physical activity attributes %(n)	Activity category	Outcome	Reported duration of follow-up	Category (months)	Pre-intervention mean±SD	Final score mean±SD	Effect size[95%CI]	Study conclusions where effect size unable to be calculated
Frank et al. [74]	27/26	62%(16)	31±7	FAI/FAIS	Amateur 23%(6) Recreational 73%(20)	Endurance (swimming)	HOS-SS	2 yr	19 to 24	44.0±21.0	85.2±16.0	-2.17[-2.87 to -1.48]	No significant change to average miles/week swimming: 0.4±0.8 [range, 0.2-1.02] pre-op; 0.36±0.74 post-op (p=0.86)
Gupta et al [78]	595/595	62%(367)	38(13-76)	Not specified	NR	NR	HOS-SS	29(24-66.1) mo	≥ 25	41.0±25.0	70.1±28.0	-1.09[-1.22 to -0.97]	
Hartigan et al. [81]	78/78	70%(57)	23(14-39)	Retroverted acetabula	NR	NR	HOS-SS	38.7(22.1–77.6) mo	≥ 25	47.3±NR	76.4±NR		Statistically significant change (p<.0001)
Hartigan et al. [82]	69/65	37%(41)	44(16-63)	Labral pathology; Subchondral cysts	NR	NR	HOS-SS	2 yr (min)	19 to 24	41.0±NR	63.0±NR		Statistically significant change (p<0.001)
Hevesi et al. [84]	303/303	67%(202)	32±12.2	Labral tear	NR	NR	HOS-SS	5 to 7.9 yr	≥ 25	NR±NR	NR±NR		Change score -29.3
Ibrahim et al. [85]	88/88	35%(31)	31(17-48)	Cam FAI	NR	NR	HOOS-SR	2.7 (1 to 8) yr	≥ 25	44.4±27.0	61.7±28.1	-0.63[-0.93 to -0.32]	
Kang et al.[88]	41/41	29%(12)	26(12-65)	FAI/FAIS; Labral tear	Athletic - >4hr/day 5 day/week in specific sport	Cutting (11%); Flexibility (49%); Asymmetric /overhead (20%); Endurance (20%)	HOS-SS	27 (16 to 53) mo	≥ 25	43%±NR	75%±NR		Statistically significant change (p=0.032)
Klingenstein et al. [90]	34/23	15%(5)	21(16-35)	FAI/FAIS	Professional 27% (9); Varsity high school 29%(10); College 44%(15)	Asymmetric /overhead	HOS-SS	25(12 to 41) mo	≥ 25	NR±NR	86.0±18.0		Statistically significant change: mean change=36 (p<0.01)

	n Baseline/Final follow up	Women %(n)	Age in years mean± SD*	Inclusion pathology	Physical activity attributes %(n)	Activity category	Outcome	Reported duration of follow-up	Category (months)	Pre-intervention mean±SD	Final score mean±SD	Effect size[95%CI]	Study conclusions where effect size unable to be calculated
Lansdown et al. [93]	707/585 (HOS-SS)	64%(456)	33±12	FAI/FAIS	NR	NR	HOS-SS	2 yr (min)	19 to 24	43.4±23.1	72.6±27.2	-1.16[-1.28 to -1.03]	
Lee et al [149]	45/41 HIPS	49%(20)	35(16-54)	FAI/FAIS; Labral tear	NR	NR	HOS-SS	92.4 (85 to 117) mo	≥ 25	51.2±NR	82.4±NR		
Levy et al. [95]	51/46	57%(29)	26±8	FAI/FAIS	Competitive runners; Recreational runners	Endurance	HOS-SS	2 yr	19 to 24	47.7±20.6	83.7±18.2	-1.84[-2.33 to -1.35]	significant change to average miles/week running & hours/week running: 9.6±6.5 miles & 4.6±3.1 hours **pre-injury;** 6.4±5.8 miles (p<0.001) & 3.0±2.4 hours post-op (p<0.001).
Lund et al. [99]	1835/1835	53%	38(9-79)	FAI/FAIS	NR	NR	HAGOS-SR	2 yr	20 to 24	36.0±23.0	60.0±28.1	-0.93[-1.00 to -0.87]	
							HAGOS-PA	2 yr	21 to 24	21.0±24.5	47.0±35.7	-0.85[-0.92 to -0.78]	
							HSAS	2 yr	22 to 24	2.5±1.9	3.3±2.0	-0.41[-0.48 to -0.34]	
Más Martínez et al. [101]	41/36	0%(0)	33±7	FAI/FAIS	NR	Cutting; Flexibility; Impingement; Asymmetric /overhead; Endurance	HOS-SS	31.3±12.2 mo	≥ 25	28.6±18.4	95.4±5.9	-4.84[-5.77 to -3.90]	
Michal et al. [102]	39/34	47%(16)	33(18-61)	FAI/FAIS; subspine impingement	NR	NR	HOS-SS	24.8(13 to 37) mo	≥ 25	10.1±32.1	78.4±22.2	-2.45[-3.09 to -1.82]	Significant change in median scores[range] from 20[0–80] to 95[27–100], p<0.0001
Nwachukwu et al. [105]	364/364	57%(208)	33±10	FAI/FAIS	NR	NR	HOS-SS	1 yr	7 to 12	51.7±23.7	78.0±23.9	-1.10 [-1.26 to -0.95]	

	n Baseline/Final follow up	Women %(n)	Age in years mean± SD*	Inclusion pathology	Physical activity attributes %(n)	Activity category	Outcome	Reported duration of follow-up	Category (months)	Pre-intervention mean±SD	Final score mean±SD	Effect size[95%CI]	Study conclusions where effect size unable to be calculated
Ortiz-Declet et al. [106]	49/40	40%(16)	49±12	Not specified	NR	Asymmetric /overhead	HOS-SS	2 yr (min)	19 to 24	47.7±26.5	64.4±28.2	-0.61[-1.03 to -0.18]	
Perets et al. [109]	49/39	80%(31)	19±5	FAI/FAIS; Borderline dysplasia; Labral tear; Hypermobility	Professional; High school; Collegiate	Cutting (15%); Flexibility (15%); Contact (6%); Asymmetric /overhead (32%); Endurance (32%)	HOS-SS	33.6(24 to 64.5) mo	≥ 25	46.8±23.4	80.1±22.9	-1.42[-1.92 to -0.92]	
Perets et al. [111]	62/62	71%(47) [HIPS]	21±8	Not specified	Professional 14%(9 hips) Collegiate 30%(20 hips); High school 56% (37 hips);	Cutting (38%); Flexibility (10%); Contact (6%); Impingement (9%); Asymmetric /overhead (12%); Endurance (25%)	HOS-SS	>5 yr	≥ 25	47.0±22.4	79.1±23.0	-1.41[-1.80 to -1.01]	
Pergaminelis et al. [112]	35/35	89%(31)	38(16-67)	Lig teres tear	NR	*[Only reported for 51%]* Cutting (23%); Flexibility (11%); Impingement (6%); Endurance (11%)	iHOT-33 SR	17.7(6 to 42) mo	13 to 18	15.0±16.1	31.9±22.6	-0.85[-1.34 to -0.36]	
Rhee et al. [115]	37/37	19%(7)	36±8	Chondral lesions	NR	NR	HOS-SS	12.7±7.3 mo	7 to 12	36.9±24.9	51.6±31.0	-0.52[-0.98 to -0.05]	

	n Baseline/Final follow up	Women %(n)	Age in years mean± SD*	Inclusion pathology	Physical activity attributes %(n)	Activity category	Outcome	Reported duration of follow-up	Category (months)	Pre-intervention mean±SD	Final score mean±SD	Effect size[95%CI]	Study conclusions where effect size unable to be calculated
Riff et al. [116]	32/32	59%(19)	35±7	FAI/FAIS	NR	Endurance	HOS-SS	2 yr (min)	19 to 24	49.2±21.2	83.3±21.4	-1.58[-2.15 to -1.02]	No significant change in average hours/week HIIT: 5.3±2.4 **pre-injury**; 5.1±3.6 post-op, (p=0.8).
Shaw et al. [120]	11/11	27%(3)	34(27-43)	Not specified	Active military service	NR	HOS-SS	6 mo	≤ 6	56.7±10.9	93.7±5.0	-4.21[-5.82 to -2.61]	
Stone et al.[151]	780/626	70%(437)	35(16-54)	FAI/FAIS; Labral tear	Recreational (74%); High school (10%); College (7%); Professional (2%)	NR	HOS-SS	92.4(85 to 117) mo	≥ 25	43.9±22.0	77.9±23.5	-2.16 [-2.60 to -1.72]	
Ukwuani et al [153]	69/64	97%(62)	22±9	FAI/FAIS	Competitive (51%); Intermediate (33%); Recreational (6%)	Flexibility	HOS-SS	23±12.2 mo	19 to 24	40.3±20.3	83.5±19.4	-2.16 [-2.60 to -1.72]	
Waterman et al. [126]	29/29	21%(6)	36±12	FAI/FAIS	NR	Asymmetric /overhead		2 yr (min)	19 to 24	38.2±23.5	79.7±28.8	-1.56[-2.15 to -0.97]	No significant change in average number of holes/ week: 49.2±36.8 pre-op; 45.9±38.8 post-op, (p value not reported).

*range is reported where no SD available; n=number of participants; SD=standard deviation; NR=Not reported; FAA=Functional Activity Assessment; HAGOS-PA/SR= The Copenhagen Hip and Groin Outcome Score – Participation in Physical Activities/ Physical Function in Sport and Recreation; HOOS-SR= Hip disability and Osteoarthritis Outcome Score – Function in Sport and Recreation ; HOS-SS= Hip Outcome Score – Sport Scale; HSAS= Hip Sports Activity Scale; iHOT-33 SR= International Hip Outcome Tool –Sports and Recreational activities; Tegner= Tegner Activity Scale; UCLA= The University of California at Los Angeles activity score; PT=physiotherapist; rehab=rehabilitation; Self-reported PA hr/week=self-reported physical activity hours/week; Lig=ligamentum; SDLP=labral preservation group; decomp=decompression; IFL=Iliopsoas fractional lengthening; comp=compensation; Rec=recreational; FAI/FAIS=femoroacetabular impingement/syndrome; min=minimum; op=operative; PRP=platelet rich plasma; wk=week; yr=year; mo=month; d=day; HIIT=High Intensity Interval Training.
