# Supplementary material for: Physical Activity Following Hip Arthroscopy in Young and Middle-Aged Adults: A Systematic Review
Source: Sports Med Open. 2020 Jan 28;6:7. doi: 10.1186/s40798-020-0234-8 (PMC6987281; doi:10.1186/s40798-020-0234-8)
Supplement: Supplementary file 5 — Additional file 5: Overview of patient-reported outcomes identified in the review. [file 40798_2020_234_MOESM5_ESM.pdf]

Additional file 5. Overview of patient-reported outcomes identified in the review.

Patient-reported outcome	Duration of recall	Scale	
HOS-SS<br>*Hip Outcome Score – Sport Scale*<br>(Martin 2006)	Over the last week	Six-point Scale 'No difficulty' to 'Unable'	Because of your hip, how much difficulty do you have with:<br>▪ Running 1 mile<br>▪ Jumping<br>▪ Swinging objects like golf club<br>▪ Landing<br>▪ Start and stop quickly<br>▪ Cutting/lateral movements<br>▪ Low impact like fast walking<br>▪ Perform activity with normal technique<br>▪ Ability to participate in desired sport for as long as you would like.<br>▪ How would you rate your current level of function compared with prior to hip problem (0 to 100%)<br>▪ How would you rate your current level of function (normal, nearly normal, abnormal, severely abnormal)
HAGOS-SR<br>*The Copenhagen Hip and Groin Outcome Score – Physical Function in Sport and Recreation*<br>(Thorborg et al 2011)	Over the last week	Five-point scale 'None' to 'Extreme'	What degree of difficulty have you experienced during the following activities due to problems with your hip and/or groin.<br>▪ Squatting<br>▪ Running<br>▪ Twisting / pivoting on WB leg<br>▪ Walking on uneven surface<br>▪ Running as fast as you can<br>▪ Bringing the leg forward and/or out to the side such as in kicking, skating, etc.<br>▪ Sudden explosive movements that involve quick movements such as acceleration, deceleration, change of direction, etc<br>▪ Situation where the leg is stretched in an outer positions (such as when the leg is placed as far away from the body as possible).
HAGOS-PA<br>*The Copenhagen Hip and Groin Outcome Score – Participation in Physical Activities*<br>(Thorborg et al 2011)	Over the last week	Five-point scale 'Always' to 'Never'	Consider to what degree your ability to participate in physical activities has been affected by your hip and/or groin pain problem.<br>Are you able to participate in your preferred physical activities…<br>▪ for as long as you would like<br>▪ at your normal performance level
HOOS-SS<br>*Hip disability and Osteoarthritis Outcome Score – Function in Sport and Recreation*<br>(Nilsdotter et al 2003)	Over the last week	Five-point scale 'None' to 'Extreme'	What degree of difficulty have you experienced with the following activities due to your hip?<br>▪ Squatting<br>▪ Running<br>▪ Twist/pivot on loaded leg<br>▪ Walking on uneven surface
iHOT-33 SR<br>*International Hip Outcome Tool –Sports and Recreational activities*<br>(Mohtadi et al 2012)	Over the last month	Visual Analogue Scale 'Extreme' to 'none'	▪ How concerned are you about your ability to maintain desired fitness level?<br>▪ How much pain do you experience in your hip after activity?<br>▪ How concerned are you that the pain in your hip will increase if you participate in sports or recreational activities?<br>▪ How much has you quality of life deteriorated because you cannot participate in sport/recreational activities?<br>▪ How concerned are you about cutting/changing direction during your sport and recreational activities?

			▪ How much has your performance level decreased in you sport and recreational activities?
Tegner<br>*Tegner Activity scale*<br>(Tegner at al 1985)	Current level	Classification of level of sport and physical activity (including work)	0=sick leave to<br>10=international elite
HSAS<br>*Hip Sports Activity Scale*<br>(Naal et al 2013)	Current highest level	Classification of level of sport activity	0=no recreational or competitive sports to<br>8=competitive sports (Elite level)
UCLA Activity score<br>*The University of California at Los Angeles activity score*	Current activity level	Classification of level of activity	1=wholly inactive, dependent on others and can not leave residence to<br>10=Regularly participates in impact sports
