# Supplementary material for: Physical Activity Following Hip Arthroscopy in Young and Middle-Aged Adults: A Systematic Review
Source: Sports Med Open. 2020 Jan 28;6:7. doi: 10.1186/s40798-020-0234-8 (PMC6987281; doi:10.1186/s40798-020-0234-8)
Supplement: Supplementary file 6 — Additional file 6: Data from intermediate time points. [file 40798_2020_234_MOESM6_ESM.pdf]

Additional file 6: Data from intermediate time points (not reported in ESM 4)

			Baseline		Time point 1				Time point 2				Time point 3			
Study	Outcome measure	Group (where applicable)	n	Mean±SD or [Range]	Reported duration	Timepoint category	n	Mean±SD or [Range]	Reported duration	Timepoint category	n	Mean±SD or [Range]	Reported duration	Timepoint category	n	Mean±SD or [Range]
**Randomised Controlled Trials**																
Mansell et al. [100]	HOS-SS	Surgical	66	52.6 [78.4 to 56.7]	6 mo	≤ 6 mo	66	47.9 [41.3 to 54.5]	1 yr	7 to 12 mo	66	52.1 [45.3 to 59]		7 to 12 mo	666	
Bennell et al. [131]	HOS-SS	Group 1 (PT rehab)	14	50.9±17.1	14 wk	≤ 6 mo	14	83.6±18.1								
	HOS-SS	Group 2 (no PT rehab)	16	52.1±16.7	14 wk	≤ 6 mo	14	70.8±18.6								
	HAGOS-SR	Group 1 (PT rehab)	14	35.9±16.9	14 wk	≤ 6 mo	14	43.9±19.3								
	HAGOS-SR	Group 2 (no PT rehab)	16	43.9±19.3	14 wk	≤ 6 mo	14	61.6±19.8								
	Tegner	Group 1 (PT rehab)	14	3.9±1.8	14 wk	≤ 6 mo	14	4.8±1.3								
	Tegner	Group 2 (no PT rehab)	16	4.3±2.2	14 wk	≤ 6 mo	14	5.1±2.0								
	HSAS	Group 1 (PT rehab)	14	31±18.0	14 wk	≤ 6 mo	14	39.5±14.2								
	HSAS	Group 2 (no PT rehab)	16	31.9±21.6	14 wk	≤ 6 mo	14	30.4±20.8								
**Prospective Studies, more than 1 arm (Only groups meeting criteria reported)**																
Redmond et al. [114]	HOS-SS	Group 1 (+PRP)	104	41.3±NR	3 mo	≤ 6mo	NR	61.4±NR								
		Group 2 (-PRP)	202	43.5±NR	3 mo	≤ 6mo	NR	61.8±NR								
Thorborg et al. [18]	HAGOS-SR		97	39.0±19.7	3 mo	≤ 6mo	91	58.4±25.1	6 mo	≤ 6mo	85	65.1±23.4				
	HAGOS-PA		97	19.8±24.2	3 mo	≤ 6mo	90	36.5±33.4	6 mo	≤ 6mo	85	47.8±35.4				
**Prospective Studies, single arm**																
Domb et al. [63]	HOS-SS		42	40.2±23.2	2yr	19 to 24mo	42	65.2±32.7								
Tahoun et al. [123]	HOS-SS		23	30.9±13.9	1yr	7 to 12 mo	23	64.8±26.3								
**Retrospective Studies, more than 1 arm (Only groups meeting criteria reported)**																
Frank et al. [71]	HOS-SS	Group 1 [Partial closure]	32	39.4±23.9	6 mo	≤ 6 mo	32	63.8±31.1	12 mo	7 to 12 mo	32	72.7±14.7				
	HOS-SS	Group 2 [Complete closure]	32	39.0±24.2	6 mo	≤ 6 mo	32	72.2±16.1	12 mo	7 to 12 mo	32	82.5±10.7				
Gupta et al. [77]	HOS-SS	Group1 [Obese]	87	25.4±22.3	3 mo	≤ 6 mo	62	51.7±35.4	1 yr	7 to 12 mo	62	43.8±36.6				
	HOS-SS	Group 2 [Control	364	42.0±24.2	3 mo	≤ 6 mo	124	58.1±31.5	1 yr	7 to 12 mo	124	71.5±28.2				

			Baseline		Time point 1				Time point 2				Time point 3			
Study	Outcome measure	Group (where applicable)	n	Mean±SD or [Range]	Reported duration	Timepoint category	n	Mean±SD or [Range]	Reported duration	Timepoint category	n	Mean±SD or [Range]	Reported duration	Timepoint category	n	Mean±SD or [Range]
Hartigan et al. [80]	HOS-SS	Group 1 [Retroversion]	59	45.7±25.5	3 mo	≤ 6 mo	59	66.9±NR	12 mo	7 to 12 mo	59	63.7±NR				
	HOS-SS	Group 2 [Normal version]	59	44.8±23.8	3 mo	≤ 6 mo	59	62.0±NR	12 mo	7 to 12 mo	59	69.4±NR				
Hartigan et al. [79]		Group 1 [Microfracture]	15	26.7±21.6	3 mo	≤ 6 mo	15	39.0±NR	1 yr	7 to 12 mo	15	59.0±NR				
		Group 2 [No microfracture]	45	42.3±26.1	3 mo	≤ 6 mo	45	52.3±NR	1 yr	7 to 12 mo	45	65.4±NR				
Lodhia et al. [97]		Group 1 [Microfracture]	35	42.1±24.18	3 mo	≤ 6 mo	35	51.5±34.5	1 yr	7 to 12 mo	35	58.6±23.4	2 yr	19 to 24mo	35	65.7±26.8
		Group 2 [Control]	70	37.6±25.1	3 mo	≤ 6 mo	70	62.1±28.7	1 yr	7 to 12 mo	70	65.4±27.1	2 yr	19 to 24mo	70	71.5±26.2
Retrospective Studies, single-arm																
Barastegui et al. [40]	HOS-SS		21	37.6±NR	6mo	≤ 6 mo	21	81.1±NR	12 mo	7 to 12 mo	21	88.6±NR	24 mo	19 to 24mo	21	90.3±NR
Chandrasekaran et al [45]	HOS-SS		22	42.3±22.3	3mo	≤ 6 mo	22	48.4±35.1	12 mo	7 to 12 mo	22	55.7±36.5				
Domb et al. [57]	HOS-SS		60	47.1±23.2	2 yr	19 to 24mo	60	76.0±25.5	67.8±7.4mo						60	
Flores et al. [35]	HOOS-SS		128	39.9±22.4	3mo	≤ 6 mo	NR	67.4±22.0	6 mo	≤ 6 mo	NR	73.3±22.8	1 yr	7 to 12 mo	122	74.1±24.0
Lund et al. [99]	HAGOS-SR		1835	36.0±23.0	1 yr	7 to 12 mo	1835	60.0±28.8	2 yr							
	HAGOS-PA		1835	21.0±24.5	1 yr	7 to 12 mo	1835	45.0±34.6								
	HSAS		1835	2.5±1.9	1 yr	7 to 12 mo	1835	3.1±2.0								
Perets et al. [111]	HOS-SS		62	47±22.4	2 yr	19 to 24mo	62	77.3±25.6								

n=number of participants; SD=standard deviation; NR=Not reported; HAGOS-PA/SR= The Copenhagen Hip and Groin Outcome Score – Participation in Physical Activities/ Physical Function in Sport and Recreation; HOS-SS= Hip Outcome Score – Sport Scale; HSAS= Hip Sports Activity Scale; Tegner= Tegner Activity Scale; wk=week; yr=year; mo=month; PT=physiotherapy; PRP=platelet rich plasma
