# Supplementary material for: Physical Activity Following Hip Arthroscopy in Young and Middle-Aged Adults: A Systematic Review
Source: Sports Med Open. 2020 Jan 28;6:7. doi: 10.1186/s40798-020-0234-8 (PMC6987281; doi:10.1186/s40798-020-0234-8)
Supplement: Supplementary file 8 — Additional file 8: Risk of bias assessment. [file 40798_2020_234_MOESM8_ESM.pdf]

Additional file 8: Quality assessment for all included studies.

				Internal Validity												
		External Validity		Performance	Detection				Attrition	Selection bias/control of confounding						
Study	Country	Representative	[1]Participation rate	Direct observation	PROM -validity/ reliability	[2]Direct observation - validity/ reliability	Blinded assessors	[3]Outcome measure	[1]Completeness	[4]Age	Location	[5]Gender	[6]Severity of Joint disease	[7]Follow-up	Single site &/or surgeon	LOE
**RCTs**																
Bennell et al. [131]	Australia	✓	✓	✓	✓	NA	✓	✓	✗	✗	✓	✗	✓	✓		2
Mansell et al. [100]	USA	✓	✓	✓	✓	NA	✓	✓	✗	✓	✓	✗	✓	✗	YES	2
**Prospective Studies, more than 1 arm**																
Chaharbakhshi et al [43]	USA	✗	✓	✓	✗	NA	✗	✓	✓	✗	✓	✓	✓	✗	YES	3
Domb et al. [58]	USA	✓	✓	✓	✗	NA	✗	✓	✓	✗	✓	✓	✓	✗	YES	3
Flores [69]	USA	✓	✓	✓	✓	NA	✗	✓	✓	✗	✓	✓	✓	✗	YES	3
Flores et al. [70]	USA	✓	✓	✓	✓	NA	✗	✓	✓	✗	✓	✗	✓	✗	YES	3
Glaws et al. [76]	USA	✓	✓	✓	✓	NA	✗	✓	✗	✗	✓	✗	✓	✗	YES	3
Kemp et al. [89]	Australia	✓	✓	✓	✓	NA	✓	✓	✗	✗	✓	✗	✗	✗	YES	3
Kierkegaard et al.[154]	Denmark	✓	✓	✓	✓	✓	✗	✓	✓	✗	✓	✓	✓	✗	YES	3
Krych et al. [91]	USA	✓	✓	✓	✓	NA	✗	✓	✓	✗	✓	✓	✓	✗	YES	3
Newman et al. [104]	USA	✗	✓	✓	✓	NA	✗	✓	✓	✓	✓	✓	✓	✗	YES	3
Redmond et al. [113]	USA	✓	✓	✓	✗	NA	✗	✓	✓	✗	✓	✗	✗	✗		3
Redmond et al. [114]	USA	✗	✓	✓	✗	NA	✗	✓	✓	✗	✗	✓	✗	✗	YES	3
Thorborg et al. [18]	Denmark	✓	✓	✓	✓	NA	✗	✓	✓	✗	✓	✓	✓	✓	YES	3
Zimmerer et al. [130]	Germany	✓	✓	✓	✓	NA	✗	✓	✓	✓	✓	✗	✓	✗	YES	3
**Prospective, single arm**																
Bennett et al. [42]	UK	✗	✗	✗	✓	NA	✗	✓	✓	✓	✓	✓	✗	✓	YES	3
Chahal et al [37]	USA	✗	✗	✓	✓	NA	✗	✓	✗	✗	✓	✗	✓	✗	YES	3
Davis et al. [53]	USA	✗	✗	✓	✓	NA	✗	✓	✓	✗	✓	✓	✓	✗	YES	3
Domb et al. [63]	USA	✓	✓	✓	✗	NA	✗	✓	✓	✗	✓	✗	✓	✗	YES	3
Ishoi et al [17]	Denmark	✓	✗	✓	✗	NA	✗	✓	✗	✓	✓	✓	✓	✗		3
Ohlin et al.[150]	Sweden	✓	✓	✓	✓	NA	✗	✓	✗	✗	✓	✗	✓	✗		3
Philippon [132]	USA	✓	✓	✓	✓	NA	✗	✓	✓	✗	✓	✗	✗	✗	YES	4
Sansone et al. [118]	Sweden	✗	✓	✓	✓	NA	✗	✓	✓	✗	✓	✗	✗	✗	YES	3

Study	Country	External Validity		Internal Validity												
				Performance	Detection				Attrition	Selection bias/control of confounding						
		Representative	[1]Participation rate	Direct observation	PROM -validity/ reliability	[2]Direct observation - validity/ reliability	Blinded assessors	[3]Outcome measure	[1]Completeness	[4]Age	Location	[5]Gender	[6]Severity of Joint disease	[7]Follow-up	Single site &/or surgeon	LOE
Sansone et al. [6]	Sweden	✗	✓	✓	✓	NA	✗	✓	✗	✗	✓	✗	✓	✗	YES	3
Tahoun et al. [123]	Spain/Egypt	✗	✗	✓	✗	NA	✗	✓	✓	✗	✓	✗	✓	✗	YES	4
Tijssen et al. [124]	Netherlands	✓	✓	✓	✗	NA	✓	✓	✓	✗	✓	✗	✗	✗	YES	3
**Retrospective, more than1 arm**																
Basques et al.[133]	USA	✓	✓	✓	✓	NA	✗	✓	✓	✗	✓	✗	✓	✗	YES	4
Beck et al. [135]	USA	✓	✓	✓	✗	NA	✗	✓	✓	✗	✓	✓	✓	✗	YES	4
Bolia et al.[136]	USA	✓	✓	✓	✗	NA	✗	✓	✓	✗	✓	✓	✓	✗	YES	4
Cancienne et al [137]	USA	✓	✓	✓	✗	NA	✗	✓	✓	✗	✓	✗	✗	✗	YES	4
Chaharbakhshi et al. [138]	USA	✓	✗	✓	✗	NA	✗	✓	✓	✗	✓	✓	✓	✗	YES	4
Chahla et al. [140]	USA	✓	✓	✓	✗	NA	✗	✓	✓	✗	✓	✗	✓	✗	YES	4
Chandrasekaran et al. [142]	USA	✓	✓	✓	✗	NA	✗	✓	✗	✗	✓	✓	✓	✗	YES	4
Chandrasekaran et al. [47]	USA	✗	✓	✓	✗	NA	✗	✓	✓	✗	✓	✓	✓	✗	YES	4
Chandrasekaran et al. [49]	USA	✗	✓	✓	✗	NA	✗	✓	✗	✗	✓	✗	✓	✗	YES	4
Chandrasekaran et al. [44]	USA	✓	✓	✓	✗	NA	✓	✓	✓	✗	✓	✓	✓	✗	YES	4
Chen et al. [50]	USA	✓	✓	✓	✗	NA	✗	✓	✓	✗	✓	✗	✓	✗	YES	4
Clapp et al.[143]	USA	✗	✓	✓	✗	NA	✗	✓	✗	✗	✓	✓	✓	✗	YES	4
Cvetanovich et al [51]	USA	✓	✓	✓	✓	NA	✗	✓	✓	✗	✓	✗	✓	✗	YES	4
Degen et al. [55]	USA	✓	✓	✓	✗	NA	✗	✓	✓	✓	✓	✓	✗	✗	YES	4
Domb et al. [60]	USA	✓	✓	✓	✗	NA	✗	✓	✗	✗	✓	✗	✓	✗	YES	4
Domb et al. [59]	USA	✓	✓	✓	✗	NA	✗	✓	✓	✗	✓	✓	✓	✗	YES	4
Domb et al. [62]	USA	✓	✓	✓	✗	NA	✗	✓	✓	✗	✓	✓	✓	✗	YES	4
Domb et al. [64]	USA	✓	✓	✓	✗	NA	✗	✓	✓	✗	✓	✓	✓	✗	YES	4
Domb et al. [65]	USA	✓	✓	✓	✗	NA	✗	✓	✓	✗	✓	✗	✓	✗	YES	4
Domb et al. [56]	USA	✓	✓	✓	✗	NA	✗	✓	✓	✗	✓	✓	✓	✗	YES	4
Domb et al. [61]	USA	✓	✓	✓	✗	NA	✗	✓	✓	✗	✓	✓	✓	✗	YES	4
Fabricant et al [68]	USA	✓	✓	✓	✗	NA	✗	✗	✓	✗	✓	✓	✓	✗	YES	4

		External Validity		Internal Validity: Performance	Internal Validity: Detection				Internal Validity: Attrition	Internal Validity: Selection bias/control of confounding						
Study	Country	Representative	[1]Participation rate	Direct observation	PROM -validity/ reliability	[2]Direct observation - validity/ reliability	Blinded assessors	[3]Outcome measure	[1]Completeness	[4]Age	Location	[5]Gender	[6]Severity of Joint disease	[7]Follow-up	Single site &/or surgeon	LOE
Frank et al. [144]	USA	✗	✓	✓	✗	NA	✗	✓	✗	✗	✓	✓	✓	✗	YES	4
Frank et al. [71]	USA	✓	✓	✓	✗	NA	✗	✓	✓	✗	✓	✓	✗	✓	YES	4
Frank et al. [72]	USA	✗	✗	✓	✗	NA	✗	✓	✓	✗	✓	✓	✗	✗	YES	4
Gupta et al. [77]	USA	✓	✓	✓	✗	NA	✗	✓	✓	✗	✓	✓	✓	✗	YES	4
Hartigan et al. [79]	USA	✓	✓	✓	✗	NA	✗	✓	✓	✗	✓	✓	✓	✗	YES	4
Hartigan et al. [80]	USA	✓	✓	✓	✗	NA	✗	✓	✓	✗	✓	✓	✓	✗	YES	4
Hassebrock et al. [145]	USA	✓	✓	✓	✗	NA	✗	✓	✓	✓	✓	✗	✓	✗	YES	4
Hevesi et al [146]	USA	✓	✓	✓	✗	NA	✗	✓	✓	✗	✓	✗	✓	✗		4
Hevesi et al [83]	USA	✓	✓	✓	✗	NA	✗	✓	✓	✗	✓	✓	✓	✗		4
Jackson et al. [86]	USA	✓	✓	✓	✗	NA	✗	✓	✓	✗	✓	✗	✗	✗	YES	4
Jackson et al. [87]	USA	✓	✓	✓	✗	NA	✗	✓	✓	✗	✓	✗	✗	✗	YES	4
Krishnamoorthy et al.[147]	USA	✓	✓	✓	✗	NA	✗	✓	✗	✗	✓	✓	✓	✗	YES	4
Kuhns et al. [92]	USA	✓	✓	✓	✗	NA	✗	✓	✓	✗	✓	✓	✓	✗	YES	4
Kunze et al.[148]	USA	✓	✓	✓	✗	NA	✗	✓	✓	✗	✓	✗	✓	✗	YES	4
Levy et al. [94]	USA	✓	✓	✓	✗	NA	✗	✓	✓	✗	✗	✓	✗	✗	YES	4
Locks et al. [96]	USA	✓	✓	✓	✗	NA	✗	✓	✓	✗	✓	✓	✓	✓	YES	4
Lodhia et al. [97]	USA	✗	✓	✓	✗	NA	✗	✓	✗	✗	✓	✓	✓	✗	YES	4
Lodhia et al. [98]	USA	✓	✗	✓	✗	NA	✗	✓	✓	✗	✓	✓	✓	✗	YES	4
Nawabi et al. [103]	USA	✗	✓	✓	✗	NA	✗	✓	✓	✗	✓	✓	✗	✗	YES	4
Perets et al. [108]	USA	✓	✓	✓	✗	NA	✗	✓	✗	✗	✓	✗	✓	✗	YES	4
Perets et al. [110]	USA	✓	✓	✓	✗	NA	✗	✓	✗	✗	✓	✓	✓	✗	YES	4
Perets et al. [107]	USA	✓	✓	✓	✗	NA	✗	✗	✓	✓	✓	✓	✓	✗	YES	4
Saltzman et al. [117]	USA	✗	✓	✓	✗	NA	✗	✓	✓	✗	✓	✗	✓	✗	YES	4
Sawyer et al. [119]	USA	✓	✓	✓	✗	NA	✗	✓	✓	✗	✓	✓	✓	✗	YES	4
Stake et al. [121]	USA	✗	✓	✓	✗	NA	✗	✗	✗	✗	✓	✓	✓	✗	YES	4

				Internal Validity												
		External Validity		Performance	Detection				Attrition	Selection bias/control of confounding						
Study	Country	Representative	[1]Participation rate	Direct observation	PROM -validity/ reliability	[2]Direct observation - validity/ reliability	Blinded assessors	[3]Outcome measure	[1]Completeness	[4]Age	Location	[5]Gender	[6]Severity of Joint disease	[7]Follow-up	Single site &/or surgeon	LOE
Stone et al. [152]	USA	✗	✓	✓	✗	NA	✗	✓	✓	✗	✓	✓	✓	✗	YES	4
Suarez-Ahedo et al. [122]	USA	✓	✓	✓	✗	NA	✗	✓	✓	✗	✓	✓	✓	✗	YES	4
Vap et al. [125]	USA	✗	✓	✓	✗	NA	✗	✓	✓	✗	✓	✓	✗	✗	YES	4
Weber et al. [127]	USA	✓	✓	✓	✗	NA	✗	✓	✓	✗	✓	✓	✓	✗	YES	4
Wu et al. [128]	China	✗	✗	✓	✗	NA	✗	✓	✗	✗	✓	✓	✓	✗	YES	4
Yoo et al. [129]	Korea	✗	✗	✓	✗	NA	✗	✓	✓	✓	✓	✓	✓	✗	YES	4
**Retrospective, single arm**																
Barastegui et al. [40]	Spain	✗	✓	✓	✗	NA	✗	✓	✓	✗	✓	✗	✗	✗	YES	
Bayley et al. [41]	Canada	✓	✓	✓	✗	NA	✗	✗	✓	✗	✓	✗	✓	✗	YES	4
Beck et al. [134]	USA	✓	✓	✓	✗	NA	✗	✓	✓	✗	✓	✗	✓	✗	YES	4
Chahla et al. [139]	USA	✓	✓	✓	✗	NA	✗	✓	✓	✗	✓	✗	✓	✗	YES	4
Chambers et al. [141]	USA	✓	✓	✓	✓	NA	✗	✓	✓	✗	✓	✓	✓	✗	YES	4
Chandrasekaran et al. [45]	USA	✗	✓	✓	✗	NA	✗	✓	✓	✗	✓	✗	✓	✗	YES	4
Chandrasekaran et al. [46]	USA	✓	✓	✓	✗	NA	✗	✓	✓	✗	✓	✗	✓	✗	YES	4
Chandrasekaran et al. [48]	USA	✗	✓	✓	✗	NA	✗	✓	✓	✗	✓	✗	✓	✗	YES	4
Cvetanovich et al. [52]	USA	✓	✓	✓	✓	NA	✓	✓	✓	✗	✓	✗	✓	✗	YES	4
Degen [54]	USA	✓	✓	✗	✗	NA	✗	✓	✗	✗	✓	✗	✓	✗	YES	4
Domb et al. [57]	USA	✓	✓	✓	✗	NA	✗	✓	✓	✗	✓	✗	✓	✗	YES	4
Domb et al. [66]	USA	✓	✓	✓	✗	NA	✗	✓	✓	✗	✓	✗	✓	✗	YES	4
Domb et al. [67]	USA	✓	✓	✓	✗	NA	✗	✓	✓	✗	✓	✗	✓	✗	YES	4
Flores et al. [35]	USA	✓	✓	✓	✓	NA	✗	✓	✗	✗	✓	✓	✓	✗	YES	4
Frank et al [73]	USA	✓	✓	✓	✗	NA	✗	✓	✓	✗	✓	✗	✓	✗	YES	4
Frank et al.[74]	USA	✓	✓	✓	✗	NA	✗	✓	✓	✗	✓	✗	✓	✗	YES	4
Frank et al. [75]	USA	✓	✓	✓	✗	NA	✗	✓	✓	✗	✓	✗	✓	✗	YES	4
Gupta et al [78]	USA	✓	✓	✓	✗	NA	✗	✓	✓	✗	✓	✗	✓	✗	YES	4
Hartigan et al. [82]	USA	✓	✓	✓	✓	NA	✗	✓	✓	✗	✓	✗	✓	✗	YES	4
Hartigan et al. [81]	USA	✓	✓	✓	✗	NA	✗	✓	✓	✗	✓	✗	✓	✗	YES	4

		External Validity		Internal Validity												
				Performance	Detection				Attrition	Selection bias/control of confounding						
Study	Country	Representative	[1]Participation rate	Direct observation	PROM -validity/ reliability	[2]Direct observation - validity/ reliability	Blinded assessors	[3]Outcome measure	[1]Completeness	[4]Age	Location	[5]Gender	[6]Severity of Joint disease	[7]Follow-up	Single site &/or surgeon	LOE
Hevesi et al. [84]	USA	✓	✓	✓	×	NA	×	✓	✓	×	×	×	✓	×		4
Ibrahim et al. [85]	Canada	✓	✓	✓	×	NA	×	✓	✓	×	✓	×	✓	×	YES	4
Kang et al.[88]	Korea	✓	✓	✓	×	NA	×	✓	✓	×	✓	×	×	×	YES	4
Klingenstein et al. [90]	USA	✓	✓	✓	×	NA	×	✓	×	×	✓	×	✓	×	YES	4
Lansdown et al. [93]	USA	✓	✓	✓	×	NA	×	✓	×	×	✓	×	✓	×	YES	4
Lee et al.[149]	Korea	×	✓	✓	×	NA	×	✓	✓	×	✓	✓	✓	×		4
Levy et al. [95]	USA	✓	✓	✓	×	NA	×	✓	✓	×	✓	✓	✓	×	YES	4
Lund et al. [99]	Denmark	✓	×	✓	✓	NA	×	✓	×	×	✓	✓	✓	×		4
Más Martínez et al. [101]	Spain	✓	✓	✓	×	NA	×	✓	✓	✓	✓	✓	✓	×	YES	4
Michal et al. [102]	Israel	×	✓	✓	×	NA	×	✓	✓	×	✓	✓	✓	×	YES	4
Nwachukwu et al. [105]	USA	✓	✓	✓	×	NA	×	✓	✓	×	✓	×	×	×	YES	4
Ortiz-Declet et al. [106]	USA	×	✓	✓	×	NA	×	✓	✓	×	✓	×	✓	×	YES	4
Perets et al. [109]	USA	×	✓	✓	×	NA	×	✓	✓	×	✓	×	✓	×	YES	4
Perets et al. [111]	USA	×	✓	✓	×	NA	×	✓	✓	×	✓	×	✓	×	YES	4
Pergaminelis et al. [112]	Australia	✓	✓	✓	✓	NA	×	✓	✓	×	✓	×	×	×		4
Rhee et al. [115]	Canada	×	✓	✓	×	NA	×	✓	✓	×	✓	×	×	×	YES	4
Riff et al. [116]	USA	✓	✓	✓	×	NA	×	✓	✓	✓	✓	×	✓	×	YES	4
Shaw et al. [120]	USA	✓	✓	✓	×	NA	×	✓	✓	✓	✓	×	×	×	YES	4
Stone et al. [151]	USA	✓	✓	✓	×	NA	×	✓	✓	×	✓	×	✓	×	YES	4
Ukwuani et al [153]	USA	✓	✓	✓	×	NA	×	✓	×	×	✓	×	✓	×	YES	4
Waterman et al. [126]	USA	✓	✓	✓	×	NA	×	✓	✓	×	✓	×	✓	×	YES	4

✓ indicates the measure was adequately addressed in the study. × indicates the measure was not adequately addressed in the study.

[1]✓ percent participation/ completion was 80% or more

[2]**NA** indicates no direct measure of PA used

[3]✓ indicates same method of ascertainment was used for all participants

[4]✓ if **range** within 18-50. × if range is outside 18-50 and not adjusted for in analysis or insufficient information

[5]✓ if gender is balanced (10% or less difference) or adjusted for in analysis; × >10% difference not adjusted for in analysis or unknown

[6]✓ if severity of OA identified in the study

				Internal Validity												
		External Validity		Performance	Detection				Attrition	Selection bias/control of confounding						
Study	Country	Representative	[1]Participation rate	Direct observation	PROM -validity/ reliability	[2]Direct observation - validity/ reliability	Blinded assessors	[3]Outcome measure	[1]Completeness	[4]Age	Location	[5]Gender	[6]Severity of Joint disease	[7]Follow-up	Single site &/or surgeon	LOE

[7]✓ where FU is the same for all study participants or lies within 10% i.e. the following acceptable ranges – 1 year follow-up, 1 month each way; 2 years follow-up = 2 months; 3 years follow-up = 3months……10 years = 10 months;

✗ differences in follow-up are >10% or unaccounted for in analysis

LOE=Level of evidence (Oxford Centre for Evidence-Based Medicine [29]); PROM=patient-reported outcome measure.
